# NAguideR: performing and prioritizing missing value imputations for consistent bottom-up proteomic analyses

**DOI:** 10.1093/nar/gkaa498

**Published:** 2020-06-11

**Authors:** Shisheng Wang, Wenxue Li, Liqiang Hu, Jingqiu Cheng, Hao Yang, Yansheng Liu

**Affiliations:** West China-Washington Mitochondria and Metabolism Research Center; Key Lab of Transplant Engineering and Immunology, MOH, Regenerative Medicine Research Center, West China Hospital, Sichuan University, Chengdu 610041, China; Yale Cancer Biology Institute, Yale University, West Haven, CT 06516, USA; West China-Washington Mitochondria and Metabolism Research Center; Key Lab of Transplant Engineering and Immunology, MOH, Regenerative Medicine Research Center, West China Hospital, Sichuan University, Chengdu 610041, China; West China-Washington Mitochondria and Metabolism Research Center; Key Lab of Transplant Engineering and Immunology, MOH, Regenerative Medicine Research Center, West China Hospital, Sichuan University, Chengdu 610041, China; West China-Washington Mitochondria and Metabolism Research Center; Key Lab of Transplant Engineering and Immunology, MOH, Regenerative Medicine Research Center, West China Hospital, Sichuan University, Chengdu 610041, China; Yale Cancer Biology Institute, Yale University, West Haven, CT 06516, USA; Department of Pharmacology, Yale University School of Medicine, New Haven, CT 06520, USA

## Abstract

Mass spectrometry (MS)-based quantitative proteomics experiments frequently generate data with missing values, which may profoundly affect downstream analyses. A wide variety of imputation methods have been established to deal with the missing-value issue. To date, however, there is a scarcity of efficient, systematic, and easy-to-handle tools that are tailored for proteomics community. Herein, we developed a user-friendly and powerful stand-alone software, *NAguideR*, to enable implementation and evaluation of different missing value methods offered by 23 widely used missing-value imputation algorithms. *NAguideR* further evaluates data imputation results through classic computational criteria and, unprecedentedly, proteomic empirical criteria, such as quantitative consistency between different charge-states of the same peptide, different peptides belonging to the same proteins, and individual proteins participating protein complexes and functional interactions. We applied *NAguideR* into three label-free proteomic datasets featuring peptide-level, protein-level, and phosphoproteomic variables respectively, all generated by data independent acquisition mass spectrometry (DIA-MS) with substantial biological replicates. The results indicate that *NAguideR* is able to discriminate the optimal imputation methods that are facilitating DIA-MS experiments over those sub-optimal and low-performance algorithms. *NAguideR* further provides downloadable tables and figures supporting flexible data analysis and interpretation. *NAguideR* is freely available at http://www.omicsolution.org/wukong/NAguideR/ and the source code: https://github.com/wangshisheng/NAguideR/.

## INTRODUCTION

Mass spectrometry (MS)-based quantitative proteomics provides a versatile approach for profiling thousands of peptides, proteins and proteoforms between different experimental conditions and disease specimens (1–3). The successful applications of quantitative proteomics, however, has been entangled with the lack of high reproducibility and consistency, which is often manifested as data missing values being generated between different technical replicates, experimental batches, biological replicates, and research groups. These missing values [or, *not available (NA)* data points] also frequently and negatively affect the subsequent analysis of proteomic data (4,5), such as hypothesis testing, principal component analysis and hierarchical clustering analysis, which routinely require complete data matrix as input.

The missing values in proteomic datasets (6) were previously discussed and ascribed to three types of causality: missing completely at random (MCAR), missing at random (MAR) and missing not at random (MNAR), based on NA frequency and signal-to-noise patterns (7,8). The missing value issue can be profound, especially in traditional shotgun proteomics where only a fraction of ionized peptides is selected for identification (9–11). Nevertheless, in the last few years, quantitative proteomics underwent a remarkable evolution, yielding a significant increase of protein detection consistency (12,13). This is due to the development of new MS methods and workflows, such as large-scale SRM/PRM measurement (14,15), retention time or spectral library-based MS1 alignment (16–20), multiplexed tandem mass tag labeling (e.g. TMT) (21), and more recently, data independent acquisition mass spectrometry (DIA-MS) exemplified by SWATH-MS (22). For example, researchers have shown consistent detection of thousands of proteins between multiple clinical samples using TMT (1,2), and samples in even larger cohort sizes (i.e. >100–1000s) using library-based MS1 alignment (23) and DIA-MS (24,25). The increased consistency of sensitivity essentially translates to much fewer missing values in the resultant sample vs. protein (or peptide) data matrix, reducing not only MCAR, MAR, but also certain MNAR occurrences.

Although the protein-level missing values has been significantly reduced with the state-of-the-art methodological developments such as DIA-MS approach, NAs are not eliminated in the data. The reasons include, e.g. (a) the scoring of peptide identification in DIA do not always reach statistical significance in every sample (even if the peptide peak group is present) (26), (b) the retention time alignment between a large number of samples might fail due to the LC variations, spray instability, etc. (27,28) and (c) protein false discovery rate (FDR) becomes much more challenging to be controlled when multiple samples are combined (29). (d) Furthermore, post-translational modifications (PTM) oriented proteomic datasets normally feature much more prevalent missing values than the bulk-protein quantification due to additional analytical difficulties. Taking phosphoproteomics as an example, phosphorylated proteins are often low abundant, biologically dynamic, and their quantitative changes are frequently subtle and site specific. Notably, the localization of phosphosite in a peptide sequence requires fragment ions carrying the particular PTM site to be detected, scored and confidently assigned, which is even more challenging for multiple samples. Therefore, missing value imputation is still indispensable for handling proteomics and phosphoproteomic datasets, even if they are generated by DIA-MS. Unfortunately, studies addressing NA imputation for such DIA datasets are currently lacking.

Herein, we aim to provide an efficient, systematic, and easy-to-handle tool that is tailored for quantitative proteomics to deal with NA imputation. Many imputation methods have been developed for omics datasets (30), such as the global approach (e.g. singular value decomposition based imputation (SVD) (31)), local approach (e.g. *k*-nearest neighbours imputation (KNN) (31)), hybrid (e.g. LinCmb (5)) and knowledge assisted approach (32). Furthermore, relevant software packages such as MSnbase (33), IMDE (34), missMS (35), ANPELA (36,37) have been available. These options being available, the bottom-up proteomic quantification is nevertheless based on the measurement of ionized peptide precursors and their fragments derived from a given protein in a biological sample where the proteins are functionally connected. However, none of the available tools have made the usage of such empirical, uniform principle of proteomics to guide the method selection for NA imputation. Other limitations of these tools may include, e.g. the lack of graphic user-friendly interface, the lack of multiple evaluation criteria for the imputed results (38), and the lack of flexibility of handle data structure of proteomics.

In this study, we present an online tool, *NAguideR*, which integrates up to 23 commonly used missing value imputation methods, namely, zero (8), minimum (3,19), column median (39), row median (39), BPCA (38), SVD (31), KNN (31), Seq-KNN (40), trKNN (41), Mice-norm (42), Mice-cart (42), MLE (43), QR (44), Mindet (45), Minprob (45), LLS (46), Impseq (47), Impseqrob (48), IRM (49), RF (50), PI (51), GRR (52), GMS (53) (see Methods and Supplementary Table S1). Most importantly, *NAguideR* provides two categories of evaluation criteria (four classic computational criteria and four empirical proteomics criteria) to assess the imputation performance of various methods. We processed three DIA-MS datasets extensively as examples to exhibit the originality and utility of this software in analyzing phosphoproteomic, peptide and protein level results. Furthermore, we include sufficient biological replicates (*N* = 10 for each study), so that NA evaluation can be performed by referring to the full datasets, benchmarking the robustness of the imputation results in those stimulated datasets with a limited number of replicates (e.g. *N* = 3). Altogether, we found that *NAguideR* recognizes the uniform knowledge in bottom-up proteomics and is helpful in guiding missing value imputation, filling a gap in the pipeline for automated analysis of massive proteomic datasets.

## MATERIALS AND METHODS

### Data collection and acquisition

Three case-study datasets acquired by DIA-MS (or SWATH-MS) (12,22) were included for testing the availability and capability of *NAguideR*. All the three datasets followed an experimental sampling schema of 10 versus 10 biological replicates. This number of replicates is much more than those in a routine proteomic experiment (e.g. *N* = 3), enabling the estimation reference for experiments with a much smaller number of replicates.

#### Dataset 1

Phosphoproteomic DIA-MS quantitative dataset for nocodazole treated cells (or ‘PhosDIA’ in short). The MS samples injected in a previous study (54) for developing the IPF, an algorithm for identifying post-translationally modified peptides, were herein re-measured by a new, powerful Orbitrap Lumos DIA platform (55) for this study. Briefly, for the experimental condition, U2OS cells (about 3–4 million cells per plate) were treated with nocodazole (Sigma-Aldrich), an anti-mitotic drug, at a final concentration of 100 ng/ml for 18 h, which inhibits microtubule dynamics and thus arrests the cell cycle at G_2_/M phase. Treated and untreated samples (*N* = 10 replicates, respectively) were collected and processed for protein digestion (54). Phosphopeptide enrichment was performed by using TiO_2_ resin (GL Sciences). The final phosphopeptides were desalted using C18 ultramicrospin columns (Nest). Phosphopeptide mixture originating from ∼10% of the starting cell materials per culturing dish was injected for DIA-MS.

The DIA-MS measurements were performed on an established system (55). Briefly, the peptide separation was performed on EASY-nLC 1200 systems (Thermo Scientific) using a self-packed analytical PicoFrit column (New Objective) (75 μm × 30 cm length). The Orbitrap Fusion Lumos Tribrid mass spectrometer (Thermo Scientific) with a NanoFlex ion source was coupled to the LC platform. Spray voltage was set to be 2000 V and heating capillary was kept at 275°C. Using the Xcalibur 4.2.47 (Thermo Scientific), the DIA-MS method consisted of a MS1 survey scan and 40 MS2 scans of variable windows. The MS1 scan range was 350–1650 *m*/*z* and the MS1 resolution was set to be 120k. The MS1 full scan AGC target value was set to be 2.0E5 and the maximum injection time was 100 ms, The MS2 resolution was set to 30 000 at *m*/*z* 200. The MS2 range was set to be 200–1800 *m*/*z* and normalized HCD collision energy was 28%. The MS2 AGC was set to be 5.0E5 and the maximum injection time was 50 ms. The default peptide charge state was set to 2. The same samples were also measured by a shotgun analysis following a previously documented method (56).

To analyze the DIA-MS results of ‘PhosDIA’, Spectronaut software (57,58) was used to generate a spectral library from both shotgun proteomic and DIA acquisitions measuring phosphoproteomic samples. The data was reported by Spectronaut with default settings and a *Q* value cut-off of 1% at both peptide and protein levels. In particular, the PTM localization score were strictly kept at above 0.75 (59) to ensure the phosphosites are localized with a certainty similar to Class I confidence (59–61). The averaged phosphopeptide enrichment efficiency were determined to be 65.07 ± 5.32% among 20 samples. All the peptide level data were quantified by Spectronaut with default settings and were subjected for NA imputation analysis.

#### Dataset 2

Protein-level SWATH-MS quantitative dataset for formaldehyde (FA) treated cells (or ‘ProtSWATH’ in short). This dataset was published in a previous study in which HeLa Kyoto cells were treated with or without 200 μM FA for 5 h (62). The SWATH-MS measurement was performed on a SCIEX 5600 plus TripleTOF instrument. The proteome changes induced by FA treatment was demonstrated to be minimal and specific, with <1% of the detected proteins showed statistically significant reductions (*P* < 0.05, Benjamini–Hochberg adjusted), presenting a challenging case for relative label free quantification at the protein level, for which the proteomic analysis has been already matured (26). To analyze ‘ProtSWATH’ dataset, a spectral library containing mass spectrometric assays for 10 000 human proteins (63) and 1% peptide and 1% protein-FDR (29) were applied to Spectronaut based analysis (57,58). In particular, the MS2 peak area of the top3 most abundant peptides were averaged and summarized for protein quantification. No PTM score was needed.

#### Dataset 3

Peptide-level SWATH-MS quantitative dataset for formaldehyde (FA) treated cells (or, ‘pepSWATH’ in short). This dataset is identical to ‘ProtSWATH’ but were summarized at the peptide precursor level with 1% FDR. However, to compare the imputed missing values by algorithms of *NAguideR* to imputation from the ‘Requantification’ option in OpenSWATH (27,64), the peptide precursor level quantities were reported after OpenSWATH analysis with ‘Requantification’ enabled, which infers the peak boundaries from the closest neighboring run after retention time alignment and quantify the fragment-ion signal within those boundaries (27), as reported before (62).

### Missing value imputation methods

To embrace multiple choices for users, a total of 23 published methods for NA imputation were integrated in *NAguideR*. Based on the implementation algorithm of these methods, they can be classified into three types (8,45): (i) *single value methods (SV methods)*, including zero, minimum, column median, row median, Mindet, Minprob, PI, which features replacing missing values by a constant or a randomly selected value; (ii) *global structure methods (GS methods)*, including SVD, BPCA, MLE, Impseq, Impseqrob, which decompose the data matrix or minimize the determinant of the covariance and then iteratively reconstruct the missing values; (iii) *local similarity methods (LS methods)*, including KNN, Seq-KNN, trKNN, LLS, QR, IRM, GRR, GMS, Mice-norm, Mice-cart, RF, which exploit local similarity structure based on the expression profiles of those objects (etc. peptides, proteins) in the data. Additionally, to facilitate the selection, these methods can be also classified into fast (zero, minimum, column median, row median, Mindet, Minprob, PI, SVD, MLE, Impseq, Impseqrob, KNN, Seq-KNN, LLS, QR, GRR) and slow ones (BPCA, trKNN, IRM, GMS, Mice-norm, Mice-cart, RF), based on the calculation of their practical time cost (Supplementary Figure S1). Detailed descriptions about all these 23 algorithms and their implementation of can be found in Supplementary Table S1.

### Evaluation Criteria

Four classic criteria and four empirical proteomic criteria are available for evaluating the performance of every imputation method that are implemented independently in *NAguideR*.

### Four classic criteria

(a1) Normalized root mean square error (NRMSE) (38). This criterion can evaluate the differences between original values and imputed values and calculated using the following formula:

(1) $$\begin{equation*}{\rm{NRMSE\;}} = \sqrt {\frac{{{\rm mean}{{\left( {{y_o} - {y_i}} \right)}^2}}}{{{\rm variance}\left( {{y_o}} \right)}}} \;\end{equation*}$$

where ${y_o}$ means original values and ${y_i}$ means imputed values. The smaller NRMSE value indicates that the method has better performance for imputation.

(a2) NRMSE based sum of ranks (SOR) (44,52). This criterion is a robust nonparametric measurement, which calculates the rank of NRMSE to compare different imputation methods:

(2) $$\begin{equation*}{\rm{SOR\;}} = \mathop \sum \limits_{i = 1}^n {{\rm Rank}_i}\left( {{\rm NRMSE}} \right)\;\end{equation*}$$

where ${{\rm Rank}_i}( {{\rm NRMSE}} )$ indicates the NRMSE ranks of different imputation methods in *i*th missing variable, n means the total number of missing variables.

(a3) Average correlation coefficient between the original and imputed values (ACC/ACC_OI) (30). By default, the Pearson correlation coefficient is calculated for measuring how strong a relationship is between the original and imputed values.

(a4) Procrustes statistical shape analysis (PSS) (52,65). This criterion is typically used to assess the similarity of two input matrix through the sum of squared differences. Herein the principal component matrix is extracted from principal component analysis (PCA) as the unsupervised input matrix for evaluating the space alteration of the original sample distribution and the imputed sample distribution.

### Four proteomic criteria

(b1) Average correlation coefficient within the different charge states of each peptide (ACC_Charge) (66). This criterion can be deduced by:

(3) $$\begin{equation*}{{\rm Peptide}_k} = \frac{{\mathop \sum \nolimits_{i = 1,\;j = 2,i \ne j}^m {\rm cor}\left( {{{\rm Charge}_i},\;{{\rm Charge}_j}} \right)}}{m}\;\end{equation*}$$

(4) $$\begin{equation*}{\rm{ACC}}\_{\rm{Charge\;}} = \frac{{\mathop \sum \nolimits_{k = 1}^n {{\rm Peptide}_k}}}{n}\;\end{equation*}$$

where the *k*th peptide has m charge states (*m* > 1), then we calculate the average correlation between every two charge states (${{\rm Charge}_i},\;{{\rm Charge}_j}$) of the *k*th peptide, n means the total number of peptides with multiple charges.

(b2) Average correlation coefficient within the different peptides of each protein (ACC_PepProt) (67). This criterion can be calculated as below:

(5) $$\begin{equation*}{{\rm Protein}_k} = \frac{{\mathop \sum \nolimits_{i = 1,\;j = 2,i \ne j}^m {\rm cor}\left( {{{\rm Peptide}_i},\;{{\rm Peptide}_j}} \right)}}{m}\;\end{equation*}$$

(6) $$\begin{equation*}{\rm{ACC}}\_{\rm{PepProt\;}} = \frac{{\mathop \sum \nolimits_{k = 1}^n {{\rm Protein}_k}}}{n}\;\end{equation*}$$

where the *k*th protein has m peptides (*m* > 1), then we calculate the average correlation between every two peptides (${{\rm Peptide}_i},\;{{\rm Peptide}_j}$) of the *k*th protein, n means the total number of proteins with multiple peptides.

(b3) Average correlation coefficient within every protein complex based on CORUM database (ACC_CORUM) (68). This criterion can be calculated as below:

(7) $$\begin{equation*}{{\rm Complex}_k} = \frac{{\mathop \sum \nolimits_{i = 1,\;j = 2,i \ne j}^m {\rm cor}\left( {{{\rm Protein}_i},\;\;{{\rm Protein}_j}} \right)}}{m}\;\end{equation*}$$

(8) $$\begin{equation*}{\rm{ACC}}\_{\rm{CORUM\;}} = \frac{{\mathop \sum \nolimits_{k = 1}^n {{\rm Complex}_k}}}{n}\;\end{equation*}$$

where the *k*th protein complex has m proteins (*m* > 1), then we calculate the average correlation between every two proteins (${{\rm Protein}_i},\;{{\rm Protein}_j}$) of the *k*th protein complex, *n* means the total number of complexes with multiple proteins that can be matched in users’ proteomics data.

(b4) Average correlation coefficient within each cluster of protein-protein interaction network based on hu.MAP database (ACC_PPI) (69). This criterion can be calculated by:

(9) $$\begin{equation*}{{\rm Cluster}_k} = \frac{{\mathop \sum \nolimits_{i = 1,\;j = 2,i \ne j}^m cor\left( {{{\rm Protein}_i},\;\;{{\rm Protein}_j}} \right)}}{m}\;\end{equation*}$$

(10) $$\begin{equation*}{\rm{ACC}}\_{\rm{PPI\;}} = \frac{{\mathop \sum \nolimits_{k = 1}^n {{\rm Cluster}_k}}}{n}\;\end{equation*}$$

where the *k*th cluster has m proteins (*m* > 1), then we calculate the average correlation between every two proteins (${{\rm Protein}_i},\;{{\rm Protein}_j}$) of the *k*th cluster, n means the total number of clusters with multiple proteins that can be matched in users’ proteomics data.

All correlation coefficients in the proteomic criteria were calculated with Pearson method by default. And a larger value, in general, indicates that the imputation method under evaluation has a better performance. After obtaining all values based on each criterion, we divided them by corresponding maximum value and returned their ranks respectively. Four classic criteria can be enabled for different data types (e.g. genomics data, proteomics data, metabolomics data, etc.), while the four proteomic criteria can be particularly applied for proteomics data. Every criterion processes its own distinctive performance evaluation of various imputation methods. Moreover, all imputation results, assessment results and figures are interactively displayed on the web panel, and downloadable for end users. More detailed information can be found in Supplementary Notes.

### Tool Implementation

All functions in *NAguideR* were compiled in R (Version 3.6.1, https://www.r-project.org/) (70), and the graphical user interface (GUI) was developed in Shiny (Version 1.2.0, https://github.com/rstudio/shiny). The web tool was deployed on a server with 64GB RAM and Genuine Intel(R) CPU E2687WV running the CentOS Linux release 7.6.1810 (Core) operating system. Users can access and process their own data freely in *NAguideR* without any login requirement through some popular web browsers, such as Google Chrome, Mozilla Firefox, Safari (Supplementary Table S2). In addition, the source codes of *NAguideR* are available on the GitHub repository: https://github.com/wangshisheng/NAguideR/ under the MIT license. Users can choose to operate this tool on their own computers, where the local GUI is working exactly the same as the online version. The detailed installation and operation manual can be found in Supplementary Notes.

### Differential expression data stimulation and analysis

Differential expression analysis of two sample groups in each dataset was performed using full datasets or randomly selected observations: (i) We used the full data (10 biological replicates in each group) to construct ‘Gold Standard’ of differentially expressed proteins/peptides. Furthermore, we randomly selected (ii) five biological replicates (‘Random 5’) and (iii) three biological replicates (‘Random 3’) in each group to implement differential expression, and then repeated this process 100 times. Then, the total 100 results for every protein/peptide in the (ii) and (iii) situations were used to infer the biological data fidelity after NA imputation, by comparing to ‘Gold Standard’ results. To generate volcano plots we used the median values of these 100 results. The statistical significance was tested by two-tailed Student's *t*-test and the *P* values were corrected for multiple testing with the Benjamini–Hochberg (BH) method (71). Proteins/peptides with BH-adjusted *P* < 0.05 and the absolute value of logarithmic fold changes with base 2 (|Log_2_(FCs)|) > 0.585 (i.e. a relative fold change of 1.5 folds) were considered to be differentially expressed. The PTM motif enrichment analysis of differentially regulated phosphosites was performed with motifeR (72), using the comparison between first three samples (according their actual acquisition time which is random in each group) and the ‘Gold Standard’ result.

### Data availability

The new mass spectrometry data of PhosDIA for this study (40 raw files) and all the spectral libraries used have been deposited to the ProteomeXchange Consortium via the PRIDE (73) partner repository with the dataset identifier PXD017476.

## RESULTS

### Overview of data analysis procedure of *NAguideR*

**Figure 1.**
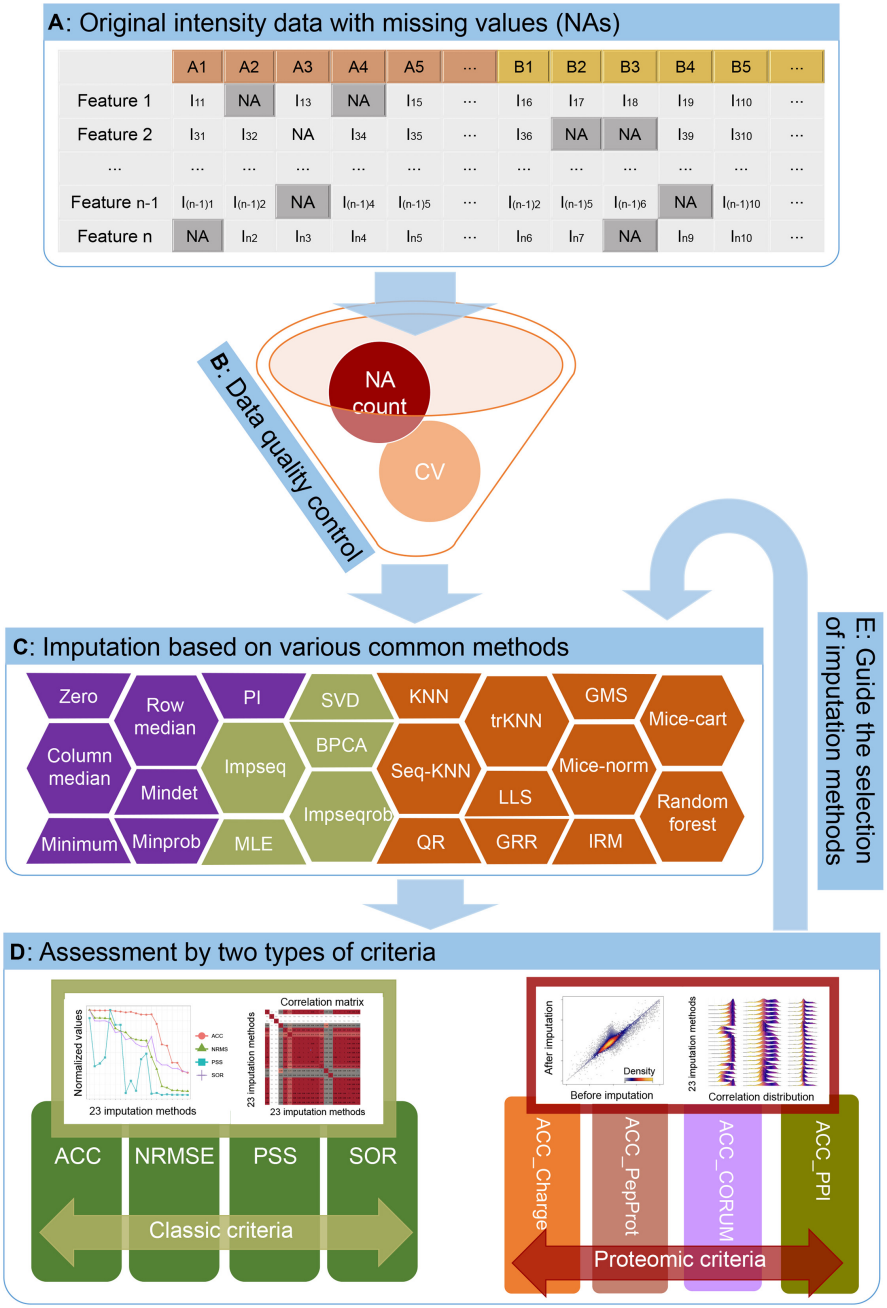
The overall workflow of *NAguideR*. (**A**) Uploading of original proteomics data with missing values (NAs). (**B**) Optional data quality control step for removing proteins/peptides with high proportion of NAs or large CV. (**C**) Missing value imputation based on the embedded methods. (**D**) Performance evaluation by multiple criteria (four classic criteria and four proteomic criteria). (**E**) The selection of well-performed imputation methods guided by the classic criteria and proteomic criteria.

Basically, there are four main steps in the data analysis process of *NAguideR* (Figure 1 and Supplementary Figure S2): (i) Data upload. In this step, users should upload the original intensity data matrix with NAs (peptides, peptides with certain PTMs, or protein identities in the rows, and sample names in the columns, Figure 1A). (ii) Initial data filtration (optional). Based on the user's choice, these proteins/peptides with excessively high proportion of NA and large coefficient of variation (CV) can be discarded in this step (Figure 1B, and see Supplementary Figure S3 for all the three example datasets). Note these criteria can be optimized iteratively upon the user's trial with *NAguideR* so that satisfactory results can be achieved. Here *NAguideR* also provides a summary note of input data quality regarding completeness before and after the filtration step (Supplementary Notes 3.3). (iii) Missing value imputation. Users can execute and obtain the matrix results of 23 imputation methods from this step (Figure 1C) with a few clicks and minimal parameter selections (Supplementary Notes 4). (iv) Result evaluation. The classic criteria and proteomic empirical criteria (see below) are applied to evaluate every result from step 3. Two comprehensive evaluation tables with ranks of each imputation method are provided to help users select suitable algorithm for their own data. Moreover, for this step, *NAguideR* implements three additional optional functions that are all at user's discretion (Supplementary Notes 5): (a) it enables users to customize the criteria and set relative weightings for specific experimental designs (e.g., if a mixture of protein standards is measured in which no *in-vivo* protein complex formation or interactions are expected); (b) it provides warning messages for users to review if the final imputation results end up with indiscriminate scores across each imputation method following classic or proteomic criteria (as a ‘Final check’ report); and (c) it allows users to directly visualize the results of a particular peptide or protein item (i.e. spiked-in standard peptides, proteins, or known housekeeping proteins like beta-actin, etc.) before and after imputation (as a ‘Targeted check’ option, Supplementary Notes 5). All results and figures in all above steps can be downloaded in the format of csv or pdf. Detailed descriptions of each step are shown in Supplementary Figure S2 and Supplementary Notes.

### Display of data completeness in three DIA datasets

To gauge the frequency of NA in DIA-MS dataset, we visualized the number of peptides/proteins quantified and missing values in each dataset using plots generated by *NAguideR* (Supplementary Figure S3). Collectively, >50 000 phosphopeptides and peptides were profiled among 20 samples in the two peptide precursor-level datasets (i.e. PhosDIA and PepSWATH) and ∼5000 proteins in ProtSWATH dataset, respectively (Supplementary Table S3). However, both of PhosDIA and PepSWATH had a large proportion of peptides with missing value in at least one sample (76.3% in PhosDIA and 55.1% in PepSWATH). The reason for high missing values in PepSWATH might be stemmed from the large, human proteome-wide assay library being used for peptide identification (63). The highest prevalence of NAs in PhosDIA could be ascribed to the significantly rewired phosphoproteome after nocodazole treatment (54) as well as the extra scoring step of phosphosite localization after peptide identification (59), presenting a most challenging case for NA imputation among the three DIA-MS datasets. In the protein-level example dataset (i.e. ProtSWATH), 4797 proteins were detected and quantified in any of the 20 samples, of which 20.4% contained at least one missing value, suggesting that the missing value problem is partially compromised by protein assignment process (e.g. Top3 summarization, see Methods), Altogether, these results from biological replicates demonstrate a pressing need for missing value imputation for proteomic experiments, even when DIA-MS is used.

### Evaluation of imputation methods by correlation-centric analysis

According to our test runs on ProtSWATH dataset, we estimated the time consumption of each NA method and thus chose 16 out of 23 methods that requires less computational procession as default methods in *NAguideR*. The seven methods left, namely BPCA, trKNN, IRM, Mice-norm, Mice-cart, GMS and RF, can be enabled upon the small lists and fast internet speed (Supplementary Figure S1, and Supplementary Notes).

**Figure 2.**
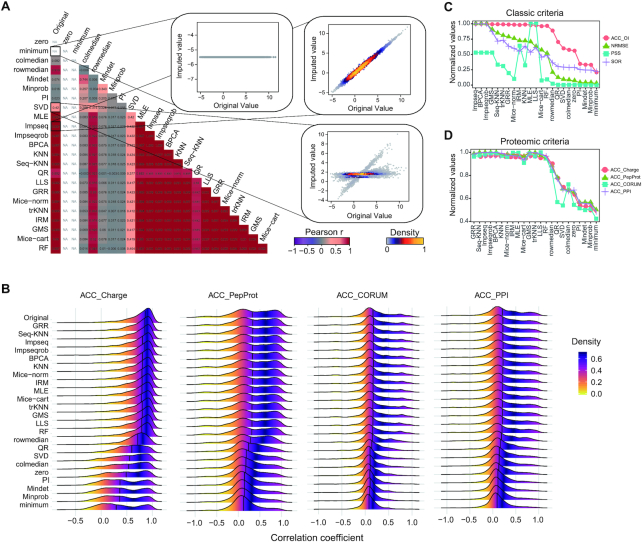
Systematic evaluation analysis of PhosDIA dataset. (**A**) Pearson correlation analysis of the original intensities and imputed intensities based on 23 methods. Density plots illustrate the correlation in detail between the original values and imputed values from minimum, SVD, and Impseq respectively as examples. NA in the correlation matrix means ‘No Result’ because the standard deviations of imputed values from zero and minimum method are equal to 0, and hence the cor function returns NA. (**B**) Comparison of the distribution of the correlation coefficient among original values and 23 imputation methods under the four proteomic criteria. The comprehensive scores distribution of 23 imputation methods under the four classic criteria (**C**) and four proteomic criteria (**D**). ‘Normalized values’ here means every score is divided by the corresponding maximum value.

To assess the NA imputation result following each method, for each of the three datasets, we firstly only extracted the complete data matrix from the original datasets and generated random missing values on it with a similar proportion of missing values existed in the original data matrix. This strategy ensured that every imputed data point will have a real reference (i.e. the original value), facilitating the comparison between imputation methods as well as the comparison between evaluation standards. All 23 imputation methods (Supplementary Table S1) were conducted on all datasets. After imputations, we compared the original values and imputed values with Pearson correlation analysis and density plots (Figure 2A and Supplementary Figure S4 for PhosDIA data, Supplementary Figure S5A for PepSWATH data, Supplementary Figure S6A for ProtSWATH data). We found that certain imputation algorithms (e.g. Impseq, BPCA, Seq-KNN and GRR) could obtain higher correlation coefficients than others (e.g. Minprob, minimum, zero and PI) across all three datasets, suggesting that certain NA methods maybe preferable for proteomic datasets, based on the classic correlation profiling between original and imputed values.

### Empirical proteomic principles facilitate the selection of NA algorithm

Following, we asked if the empirical bottom-up proteomic principles can be employed to inspect the imputation outcome. We extracted and tested correlations between peptide or protein entries based on quantitative consistency between different charge-states of the same peptide, different peptides belonging to the same proteins, and individual proteins participating functional complexes and interactions (see the example of correlation between charge states in Supplementary Figure S7 and Methods). Similar but more discriminative results compared to the correlation matrix above were obtained, which was based on the correlation coefficient distribution following proteomic criteria, supporting that the distributions from such as GRR, Seq-KNN, Impseq, BPCA were more similar to the original results (Figure 2B for PhosDIA data, Supplementary Figure S5B for PepSWATH data and Supplementary Figure S6B for ProtSWATH data). We then compared the proteomic criteria to the four classic computational criteria for NA imputation, namely NRMSE, SOR, ACC_OI and PSS, using the ranked normalized scores for each method (Figure 2C, D for PhosDIA data, Supplementary Figure S5C-S5D for PepSWATH data, and Supplementary Figure S6C, D for ProtSWATH data). Table 1 lists the corresponding ranking results in all three datasets, showing a consistent result that Impseq, Impseqrob, BPCA, trKNN, Seq-KNN and GRR were top-ranked. Interestingly, although both criteria were able to recognize a few favorable NA imputation algorithms in each dataset, the proteomic criteria based on varied mass spectrometric or biological rules generated more consistent evaluation between criteria than the classic criteria metrics. Considering the additional benefits of applying proteomic criteria, such as direct application, easy biological interpretation, and the facilitated communication between researchers, we deem the proteomic criteria efficiently help the user to select NA imputation method. Therefore, we have included both classic criteria and the above four proteomic criteria results in *NAguideR*.

**Table 1.** Evaluation ranks of 23 imputation methods on the basis of the classic criteria and the proteomic criteria applied to the three example datasets (i.e. PhosDIA, PepSWATH, and ProtSWATH)

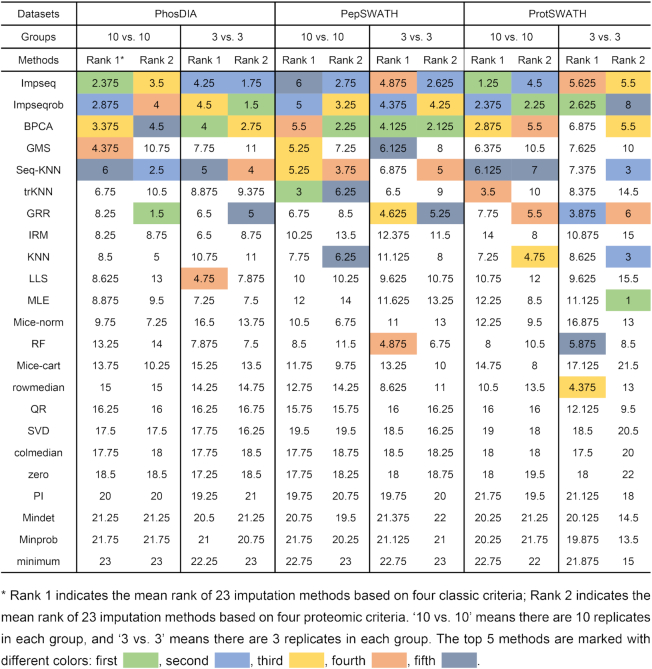

Furthermore, we assessed the robustness of proteomic criteria in evaluating the outcome of NA imputation. To do this, we randomly produced missing values on the three datasets from the proportion of 5–70% at the whole data matrix level, and in step of 5%. At each step, we repeated the imputation and evaluation process. The results (Supplementary Figure S8A) suggested that the top-ranked methods performed well and consistently across all varying missing proportions in PhosDIA data, and that the differences of scores resulted from different methods under the four proteomic criteria became larger as missing proportions increased. Similar results were obtained from PepSWATH (Supplementary Figure S8B) and ProtSWATH datasets (Supplementary Figure S8C), which are both deemed less challenging than PhosDIA dataset considering their NA prevalence (Supplementary Figure S3). Thus, proteomic criteria are robust to datasets with different extent of NA prevalence.

**Figure 3.**
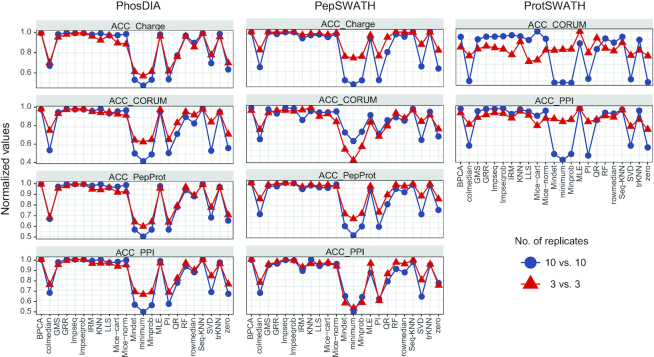
The score distribution of every imputation method based on the proteomic criteria in the three proteomics datasets with different biological replicates. Left panel: PhosDIA, middle: PepSWATH, right: ProtSWATH. ‘Normalized values’ denotes that every score is divided by corresponding maximum value. ’10 versus 10’ means that there are 10 replicates in each group (marked with darkblue color), and ‘3 versus 3’ means that there are three replicates in each group (marked with red color).

In a typical proteomic experimental design, a limited number of biological replicates such as *N* = 3 is frequently used. Hence, in all three datasets, we evaluated the robustness of *NAguideR* results by simply using the first three samples of each group (injected with a random order, thus presenting a stopping point for a routine *N* = 3 proteomic investigation). As shown in Figure 3, we found the four proteomic criteria used in *NAguideR* can provide a discriminant score estimation for each imputation method in the ‘3 versus 3’ datasets. And the resultant score distribution between different NA methods largely agrees to the results from ‘10 versus 10’ datasets. Additionally, both classic and proteomic criteria yielded similar ranking results (Table 1, Supplementary Figure S9). Altogether, these results support the feasibility of *NAguideR* and its proteomic criteria in dealing with experiments with limited biological replicates.

In summary, we introduced effective, proteomic principle-derived criteria for estimating the performance of different NA imputation methods, which shows robustness in datasets of varied NA prevalence and limited biological replicates.

### Direct application of *NAguideR* facilitates relative proteomic quantification

**Figure 4.**
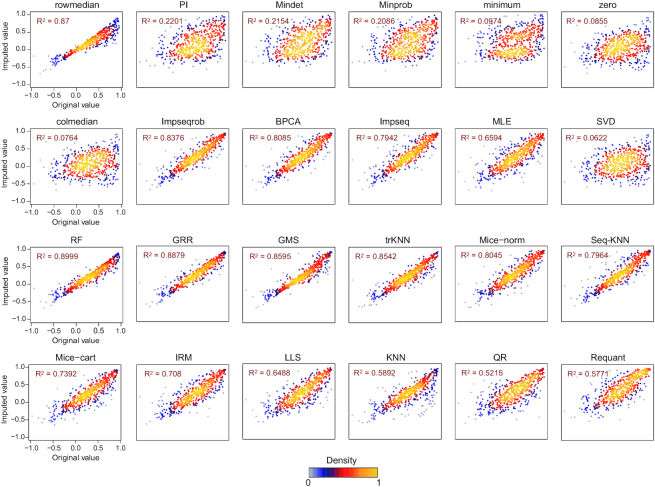
Across sample, quantitative correlation coefficients obtained by different NA imputation methods. Comparisons of original values and imputed values of the quantitative correlation coefficients are shown which are derived under ACC_Charge criterion by the 23 imputation methods and ‘Requantification’ method for the pepSWATH dataset. The adjusted *R* squared (*R*^2^) of each result was also obtained by ‘lm’ function and shown for every imputation method.‘Requant’ denotes ‘Requantification’ method in OpenSWATH software.

The above analysis based on referencing the *in-silico* deleted original values demonstrated the usage of *NAguideR* and its proteomic criteria, paving the way to address technical and biological questions in label-free quantification. Previously, the ‘Requantification’ step from OpenSWATH (27,64) and TRIC algorithms was used to impute the missing data at MS2 level for DIA-MS. ‘Requantification’ essentially infers the MS2 peak boundaries from the closest neighboring run after retention time alignment and quantifies the fragment-ion signal within those boundaries (27). We therefore compared ‘Requantification’ to the 23 NA imputation methods supported in *NAguideR*. To survey whether the imputed data points added more variation to the quantification, we plotted intra charge-state correlation of a given peptide precursor quantified across samples before and after NA imputation, following the direct application of different imputation methods including ‘Requantification’ (Figure 4). This across sample correlation can be also assessed by its average variability for all peptides (Supplementary Figure S10). The results interestingly indicate that the ‘Requantification’ method only ranked in the middle among the established 23 imputation methods (Figure 4 and Supplementary Figure S10). Thus, alternative NA imputation algorithms should be considered for DIA-MS, such as those provided by *NAguideR* under the four proteomic criteria.

**Figure 5.**
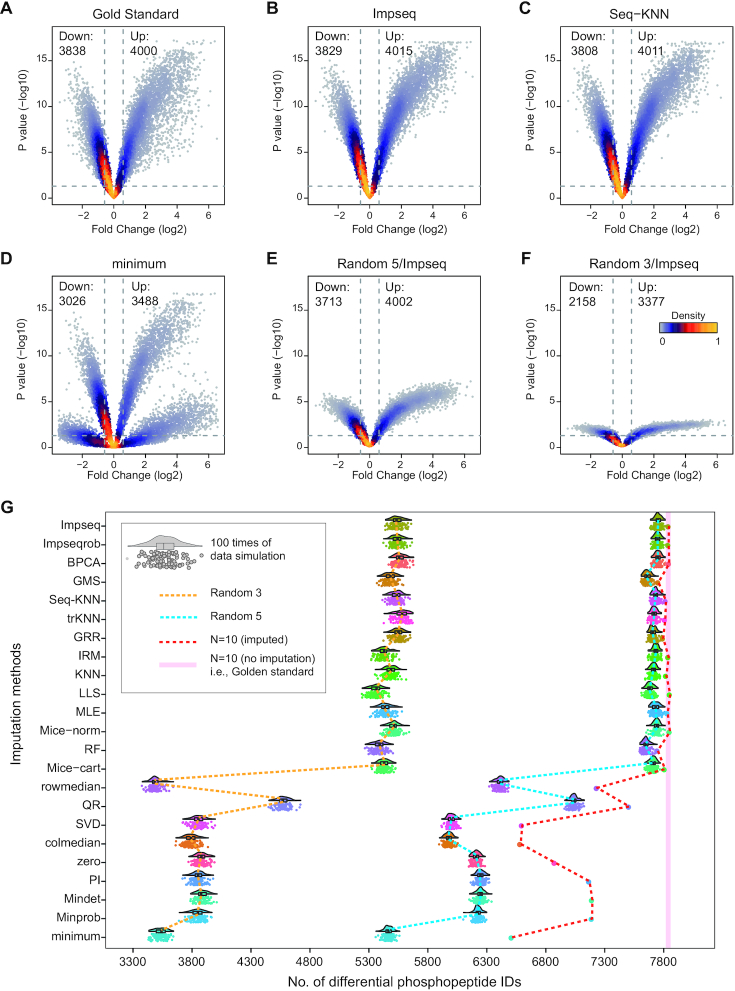
Differential expression and simulation analysis of PhosDIA dataset. Volcano plots of original full data (labelled as ‘Gold Standard’) (**A**), imputed data from Impseq method (**B**), Seq-KNN method (**C**), minimum method (**D**), imputed data of randomly selected five biological replicates (labelled as ‘Random 5’) (**E**) and 3 biological replicates (labeled as ‘Random 3’) (**F**) in each group from Impseq method. (‘Down’ means down-regulated phosphopeptides, ‘Up’ means up-regulated phosphopeptides). (**G**) Cloud-rain plots indicating the number of differentially expressed peptides for the 100 randomly selected datasets by ‘Random 5’ and ‘Random 3’. Solid pink line means the number of differentially expressed peptides from gold standard samples. Dashed lines of red, blue and yellow indicate the distribution of the numbers of differentially expressed peptides from each imputation method with all, Random 5 and Random 3 samples, respectively.

We next address how different imputation methods impact differential expression analysis. Applying *NAguideR* on all three DIA-MS datasets, we obtained completed data matrixes which can be then analyzed by the standard student t-tests between experimental and control groups. Herein we focused on the PhosDIA dataset, which profiled the phosphoproteome following the cell cycle arrest that was well-studied (54,74). Volcano plots in Figure 5A–F and Supplementary Figure S11 illustrated that, the *P*-values between groups for the same original dataset (i.e. PhosDIA) but imputed by different methods can be distinctive. Accordingly, the volcano shapes derived from top-ranked imputation methods (e.g. Impseq, Seq-KNN) were similar to that from ‘Gold Standard’ (i.e. the original full dataset without NA imputed). In stark contrast, the shapes from low-ranked methods (e.g. minimum) revealed significantly skewed *P*-values and therefore reduced efficiency in determining differential expression. Subsequently, from *N* = 10 replicates per group in PhosDIA, we randomly selected five or three observations by 100 times per group (i.e. ‘Random 5’ and ‘Random 3’) and performed the same statistical test. As expected, less-individuals per group reduce statistical significance (Figure 5A–F, median from 100 selections). We noticed that the worst NA imputations (such as minimum) often presented bifurcate and applanate volcano patterns. To further depict the differences in the number of differentially expressed peptides we stimulated the differential phosphopeptide lists based on all ‘Random 5’ and ‘Random 3’ selections respectively (Figure 5G). The results intriguingly indicate (a) both the inefficient NA imputation methods and the low biological replicates could weaken the capacity and power of detecting differential expression peptides; (b) the non-suitable imputation methods can significantly impair the differential expression analysis, even with *N* = 10 replicates (e.g. by reporting <15% significant phosphopeptide identities between groups). (c) With an ideal NA imputation, five biological replicates (*N* = 5 per group) may be already sufficient in this PhosDIA dataset tested, because the *N* = 5 comparison reported similar number of significant identities compared to the *N* = 10 scenario. Finally, because of the extensively investigated phosphoproteomic change following nocodazole treatment, we compared the motif enriched in the lists of differential phosphopeptides. Similar motifs to a previous study (74) were identified. Herein, this motif enrichment analysis is helpful to discern if those best NA methods are too aggressive in reporting regulated phosphopeptides. We found that ‘Seq-KNN’ (an example of the best NA methods) essentially identified 1494 (i.e. >35%) more phosphopeptide as the significantly regulated hits than ‘zero’ identified (an example of the worst NA methods), if we use the data of first three biological replicate samples based on MS injection time for both methods. Nevertheless, the separated motif analysis of these additional 35% phosphospeptides by ‘Seq-KNN’ yielded 13 motifs, 12 of which can be identified by *N* = 10 replicates in ‘Golden standard’ dataset (Supplementary Figure S12). This result thus suggests that those better NA methods supported by *NAguideR* can efficiently facilitate differential expression analysis and biological research.

In summary, the direct application of *NAguideR* promotes prioritizing both NA imputation method (such as ‘Requantification’) and protein/peptide candidates with real biological regulations.

## DISCUSSION

Reproducibility is a cornerstone for scientific research. The MS-based proteomics, however, often generates missing-value datasets between samples and conditions. Despite of the recent technical developments such as DIA-MS, missing values still present a major problem especially in MS datasets profiling protein PTMs. To date, efficient tools that are tailored for proteomics community are rather limited in this regard. In this study, we developed an open source and user-friendly toolkit, *NAguideR*, which implemented 23 missing value imputation methods that are frequently used and eight evaluation criteria, aiming to help scientists select the most appropriate imputation methods during data analysis. We made *NAguideR* to be conveniently accessed through both web tool and stand-alone software version, depending on the data size and internet speed.

There are two main aspects that we consider when choosing these imputation methods: First, all methods should be commonly applied and implemented in many peer-reviewed packages, e.g. MSnbase (33), impute (31), GMSimpute (53); second, as the missing values in proteomics data are generated following different complex mechanisms, these methods should include various families of imputation procedures, which can be potentially used for diverse types of missing values. For example, kNN (40), MLE (43) were proposed to be functional in imputing MCAR/MAR values; MinDet (45) and MinProb (45) were designed initially for handling MNAR values, while GMS (53) does not require specific designation of missing values pattern. In addition, different methods may have their advantages and disadvantages. For example, *SV methods* are relatively simple and fast for large-cohort experiments, but they may introduce severe bias in data and fail to meet certain hypotheses of statistical tests. On the other hand, *GS methods* and *LS methods* generally perform better, but *GS methods* assume the existence of a global covariance structure among all samples or objects (i.e. proteins/peptides/genes) and *LS methods* assume that a strong local correlation exists between objects in the expression matrix. Thus, when the assumptions are not appropriate, their imputation may become less accurate (Supplementary Table S1). Users of *NAguideR* can adjust the method selection based on these advantages and disadvantages. Of note, the practical proteomic datasets could be highly heterogenous between users due to the different sample types, quality, experimental designs, mass spectrometers used and etc. There is unlikely a one-fits-all solution for imputing NAs in all variable datasets. Thus, besides a simple ‘Input data check’ of data quality such as NA prevalence and data variation as well as a ‘Final check’ about result heterogeneity, *NAguideR* implements the 23 NA imputation methods without preference. Users can therefore compare the imputation results of different algorithm through global correlation scores as well as individual data inspection (e.g. through ‘Targeted check’ option) for selecting a method preferable to their own data.

Besides algorithm integration and flexible implementation, the evaluation step of *NAguideR* is a significant added value compared other solutions such as the individual R-packages, because this step uniquely guides users to select NA imputation method using common rules of bottom-up proteomics by visualizing their own data before and after imputation. The four proteomic criteria were found to generate moderate correlation coefficients that are potentially more discriminative than the extremely skewed correlations between original and imputed results (e.g. those in Figure 2A). The four proteomic criteria can be directly applied and inspected to an entire dataset, avoiding the potential bias of those computational evaluations focusing on those peptide/protein entries (with no NAs at all) whose concentrations tend to be more abundant in the human proteome. Moreover, two peptide-level criteria (i.e. correlation between different charge-states of the same peptide and between different peptides belonging to the same proteins) generated quite consistent results to the two protein-level metrics (i.e., correlation coefficient within each protein complex and within cluster of protein–protein interaction network) in our tested datasets, suggesting *NAguideR* could generate reliable results in selecting NA methods for both peptide- and protein-level data (Table 1). In addition, the usage of *NAguideR* was evaluated to be robust in data with high NA prevalence and with limited numbers of biological replicates (Figure 3 and Supplementary Figure S8), and may facilitate the biological investigation involving differential proteomic and phosphoproteomic measurements (Figure 5G and Supplementary Figure S12). Interestingly, several NA imputation methods such as Seq-KNN, Impseqrob, and Impseq offered better results than those sub-optimal and low-performance algorithms in all DIA datasets (including the simulated datasets with limited biological replicates), underscoring their value in future proteomic analysis. It should be stressed that, because of the multiple NA method integration, NAguideR provides the opportunity to reveal if there are certain methods that are mutually comparable, but significantly better than others. This might implicate a fact that applying one of these favorable NA methods could be sufficient for many datasets. Finally, *NAguideR* reserves the potential of updating current methods, integrating additional methods and assessment criteria in the future, and can be useful in aiding the bioinformatic efforts developing new NA algorithms.

We anticipate that *NAguideR* could greatly facilitate the multi-omics studies especially the proteomic research in dealing with NA issue and assist biologists or clinicians with less computational background in analyzing samples at a high throughput.

## DATA AVAILABILITY

*NAguideR* is an open source platform, which initiative available from: https://github.com/wangshisheng/NAguideR under the MIT license. The detailed tutorial about this tool can also be found here: https://github.com/wangshisheng/NAguideR/blob/master/NAguideR_Manual.pdf.

## Supplementary Material

gkaa498_Supplemental_File
