# Supplementary material for: NAguideR: performing and prioritizing missing value imputations for consistent bottom-up proteomic analyses
Source: Nucleic Acids Res. 2020 Jun 11;48(14):e83. doi: 10.1093/nar/gkaa498 (PMC7641313; doi:10.1093/nar/gkaa498)
Supplement: gkaa498_Supplemental_File [file gkaa498_supplemental_file.pdf]

~Supplementary material~

NAguideR: performing and prioritizing missing value imputations for consistent bottom-up proteomic analyses

Shisheng Wang[1], Wenxue Li[2], Liqiang Hu[1], Jingqiu Cheng[1], Hao Yang[1,*] and Yansheng Liu[2,3,*]

[1] West China-Washington Mitochondria and Metabolism Research Center; Key Lab of Transplant Engineering and Immunology, MOH, West China Hospital, Sichuan University, Chengdu, 610041, China

[2] Yale Cancer Biology Institute, Yale University, West Haven, CT, 06516, USA

[3] Department of Pharmacology, Yale University School of Medicine, New Haven, CT, 06520, USA

Corresponding Author

*Email address: yanghao@scu.edu.cn; yansheng.liu@yale.edu.

Table of Contents

I. Supplementary notes

1. Data Preparation

2. Import Data

3. NA Overview

4. Methods

5. Results and Assessments

6. Help

7. How to run this tool locally?

II. Supplementary tables and figures

Table S1. Description of 23 missing value imputation methods.

Table S2. The summary of NAguideR tested on different operation systems and browsers.

Table S3. The number of detected peptides/proteins and the proportion of missing values in each data set.

Figure S1. Distribution of the time consumption of each imputation method.

Figure S2. Illustration of major steps of the data analysis process in NAguideR.

Figure S3. Distribution of missing values in all the three example datasets.

Figure S4. Comparisons of original values and imputed values of every peptide from every imputation method on the extracted complete data matrix from PhosDIA.

Figure S5. Systematic evaluation analysis of the pepSWATH dataset.

Figure S6. Systematic evaluation analysis of the ProtSWATH dataset.

Figure S7. Comparisons of original values and imputed values of the correlation coefficients among peptides that are derived under ACC_Charge criterion across every imputation method that was directly applied on the full PhosDIA dataset.

Figure S8. Evaluation of every imputation method across different missing proportions on the three proteomics datasets under the proteomic criteria (A: PhosDIA, B: PepSWATH, C: ProtSWATH).

Figure S9. The score distribution of every imputation methods based on the classic criteria in the three proteomics datasets with different biological replicates (Left: PhosDIA, middle: PepSWATH, right: ProtSWATH).

Figure S10. Comparison of the root mean square error (RMSE) of the average correlation coefficients across sample among each method on the pepSWATH data set.

Figure S11. Volcano plots examples for differential expression analysis in PhosDIA (following Figure 5).

Figure S12. Motif analysis of the differentially expressed peptides in the PhosDIA dataset.

III. References

I. Supplementary notes

NAguideR integrates up to 23 commonly used missing value imputation methods (described in Table S1) and provides two categories of evaluation criteria (four classic computational criteria and four empirical proteomics criteria) to assess the imputation performance of various methods. Here we present the detailed introduction and operation of *NAguideR*, by which users can follow to analyze their own data freely and conveniently.

Users can visit this site: http://www.omicsolution.org/wukong/NAguideR. Then the website homepage can be shown like this:

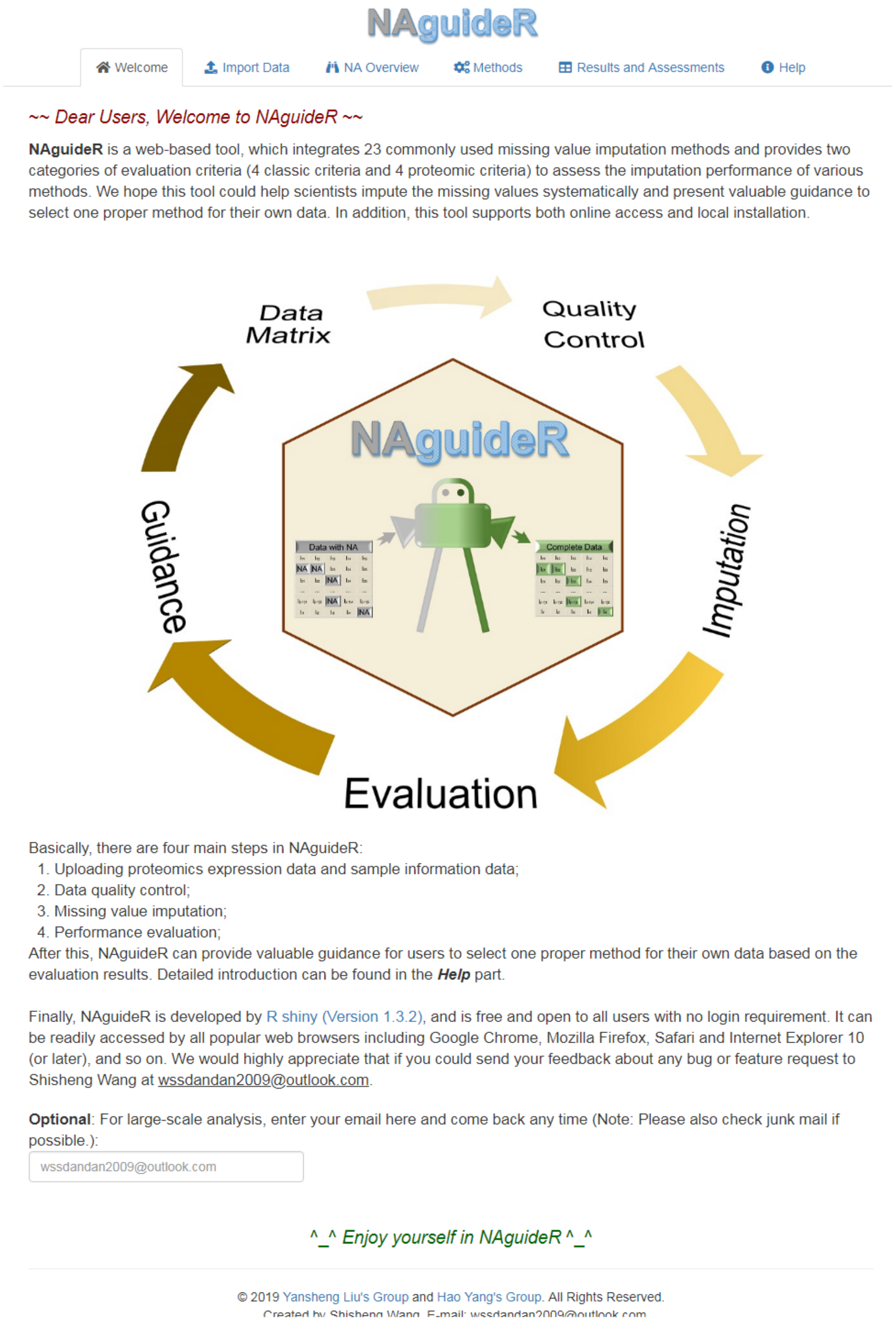

1. Data Preparation

NAguideR supports four basic file formats (.csv, .txt, .xlsx, .xls). Before analysis, users should prepare two required data: (i) Proteomics expression table for quantification; (ii) Sample information. The data required here could be readily generated based on results of several popular tools such as MaxQuant (20), PEAKS (21), Spectronaut (22), DIA-NN (23), OpenSWATH (24), and so on. The users then can upload the two data into *NAguideR* with right formats respectively and start subsequent analysis.

1.1 Expression data

There are currently four types of proteomics expression data supported in *NAguideR* (i.e., 'Peptides+Charges+Proteins', 'Peptides+Charges', 'Peptides+Proteins', 'Proteins'), among which the main differences are the first few columns. In addition, users may upload other kinds of omics data (e.g., genomics, metabolomics), for which they can just need to choose the fifth type ('Others'). Please note, the fifth type cannot generate the results based on the proteomic criteria.

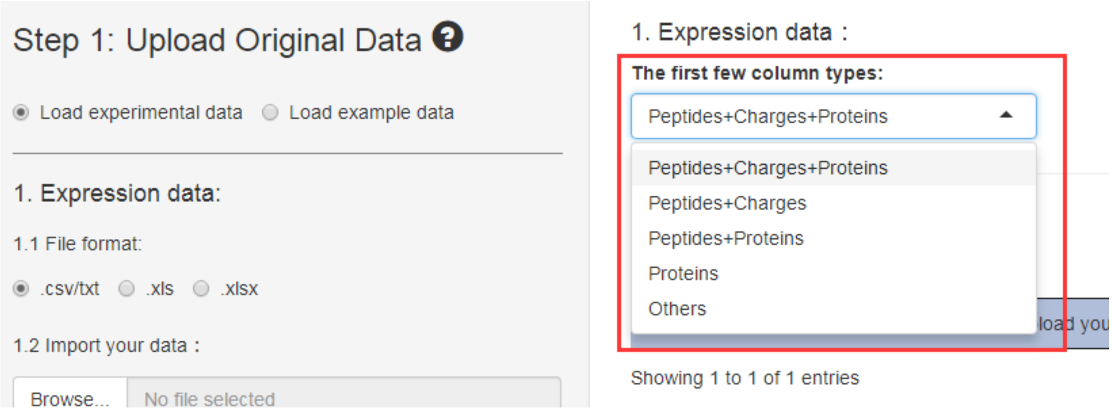

1.1.1 Expression data with peptide sequences, peptide charge states, and protein ids

In this situation, peptide sequences, peptide charge states, and protein ids are sequentially provided in the first three columns of input file. Peptide sequences in the first column can be peptides with any post-translational modification (PTM, written in any routine format) or stripped peptides (sequences without PTM). The second column is peptide charge status. The protein ids in the third column should be UniProt ids. From the fourth column, peptides/proteins expression intensity or signal abundance in every sample should be listed. The data structure is shown as below:

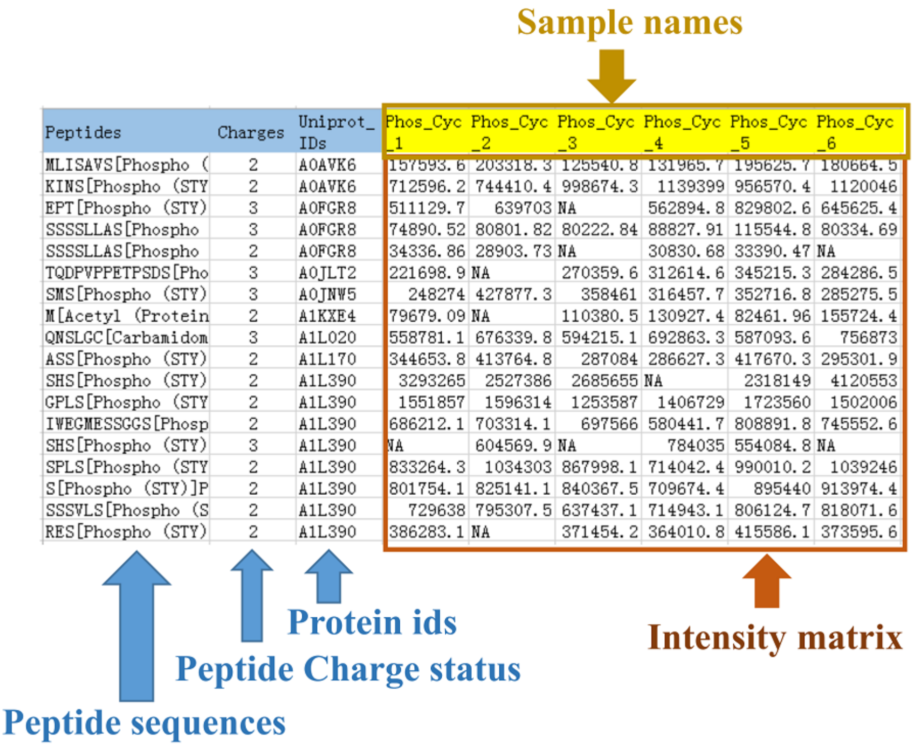

Peptides	Charges	Uniprot_IDs	Phos_Cyc_1	Phos_Cyc_2	Phos_Cyc_3	Phos_Cyc_4	Phos_Cyc_5	Phos_Cyc_6
MLISAVS[Phospho (	2	A0AVK6	157593.6	203318.3	125540.8	131965.7	195625.7	180664.5
KINS[Phospho (STY	2	A0AVK6	712596.2	744410.4	998674.3	1139399	956570.4	1120046
EPT[Phospho (STY)	3	A0FGR8	511129.7	639703	NA	562894.8	829802.6	645625.4
SSSSLLAS[Phospho	3	A0FGR8	74890.52	80801.82	80222.84	88827.91	115544.8	80334.69
SSSSLLAS[Phospho	2	A0FGR8	34336.86	28903.73	NA	30830.68	33390.47	NA
TQDPVPPETPSDS[Pho	3	A0JLT2	221698.9	NA	270359.6	312614.6	345215.3	284286.5
SMS[Phospho (STY)	3	A0JNW5	248274	427877.3	358461	316457.7	352716.8	285275.5
M[Acetyl (Protein	2	A1KXE4	79679.09	NA	110380.5	130927.4	82461.96	155724.4
QNSLGC[Carbamidom	3	A1L020	558781.1	676339.8	594215.1	692863.3	587093.6	756873
ASS[Phospho (STY)	2	A1L170	344653.8	413764.8	287084	286627.3	417670.3	295301.9
SHS[Phospho (STY)	2	A1L390	3293265	2527386	2685655	NA	2318149	4120553
GPLS[Phospho (STY	2	A1L390	1551857	1596314	1253587	1406729	1723560	1502006
IWEGMESSGGS[Phosp	2	A1L390	686212.1	703314.1	697566	580441.7	808891.8	745552.6
SHS[Phospho (STY)	3	A1L390	NA	604569.9	NA	784035	554084.8	NA
SPLS[Phospho (STY	2	A1L390	833264.3	1034303	867998.1	714042.4	990010.2	1039246
S[Phospho (STY)]P	2	A1L390	801754.1	825141.1	840367.5	709674.4	895440	913974.4
SSSVLS[Phospho (S	2	A1L390	729638	795307.5	637437.1	714943.1	806124.7	818071.6
RES[Phospho (STY)	2	A1L390	386283.1	NA	371454.2	364010.8	415586.1	373595.6

1.1.2 Expression data with peptide sequences and peptide charge states

Similar to the above situation, peptide sequences and peptide charge status are sequentially provided in the first two columns of input file. Peptide sequences in the first column can be peptides with post-translational modification (PTM, written in any routine format) or stripped peptides (without PTM). The second column is peptide charge states. From the third column, peptides/proteins expression intensity or signal abundance in every sample should be listed. The data structure is shown as below:

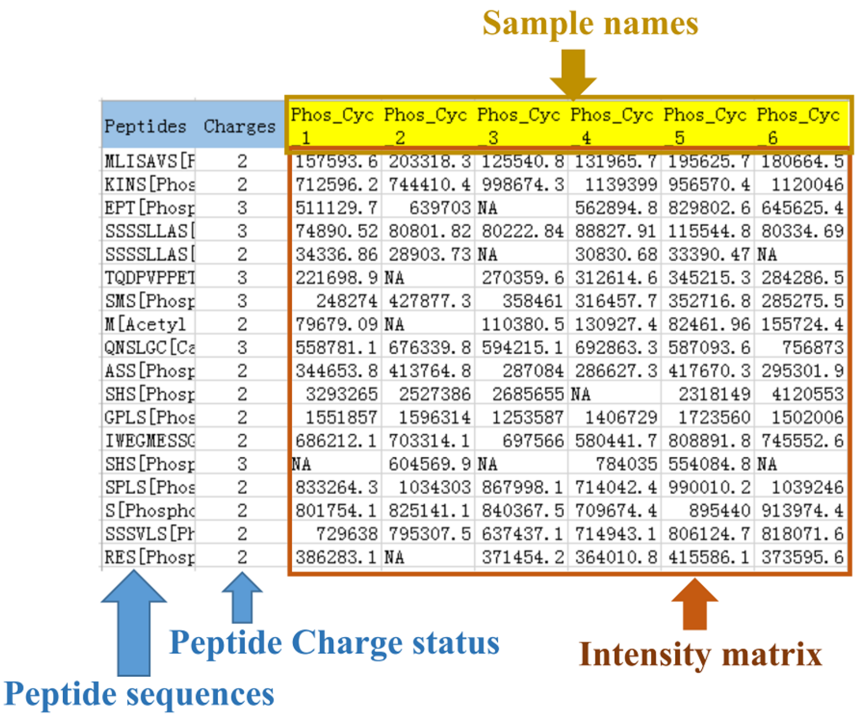

Peptides	Charges	Phos_Cyc_1	Phos_Cyc_2	Phos_Cyc_3	Phos_Cyc_4	Phos_Cyc_5	Phos_Cyc_6
MLISAVS[F	2	157593.6	203318.3	125540.8	131965.7	195625.7	180664.5
KINS[Phos	2	712596.2	744410.4	998674.3	1139399	956570.4	1120046
EPT[Phosp	3	511129.7	639703	NA	562894.8	829802.6	645625.4
SSSSLLAS[	3	74890.52	80801.82	80222.84	88827.91	115544.8	80334.69
SSSSLLAS[	2	34336.86	28903.73	NA	30830.68	33390.47	NA
TQDPVPPET	3	221698.9	NA	270359.6	312614.6	345215.3	284286.5
SMS[Phosp	3	248274	427877.3	358461	316457.7	352716.8	285275.5
M[Acetyl	2	79679.09	NA	110380.5	130927.4	82461.96	155724.4
QNSLGC[Ca	3	558781.1	676339.8	594215.1	692863.3	587093.6	756873
ASS[Phosp	2	344653.8	413764.8	287084	286627.3	417670.3	295301.9
SHS[Phosp	2	3293265	2527386	2685655	NA	2318149	4120553
GPLS[Phos	2	1551857	1596314	1253587	1406729	1723560	1502006
IWEGMESSG	2	686212.1	703314.1	697566	580441.7	808891.8	745552.6
SHS[Phosp	3	NA	604569.9	NA	784035	554084.8	NA
SPLS[Phos	2	833264.3	1034303	867998.1	714042.4	990010.2	1039246
S[Phospho	2	801754.1	825141.1	840367.5	709674.4	895440	913974.4
SSSVLS[Ph	2	729638	795307.5	637437.1	714943.1	806124.7	818071.6
RES[Phosp	2	386283.1	NA	371454.2	364010.8	415586.1	373595.6

1.1.3 Expression data with peptide sequences, and protein ids

Under this circumstance, peptide sequences, and protein ids are sequentially provided in the first two columns of input file. Peptide sequences in the first column can be peptides with post-translational modification (PTM, written in any routine format) or stripped peptides (without PTM). The protein ids in the second column should be UniProt ids. From the third column, peptides/proteins expression intensity or signal abundance in every sample should be listed. The data structure is shown as below:

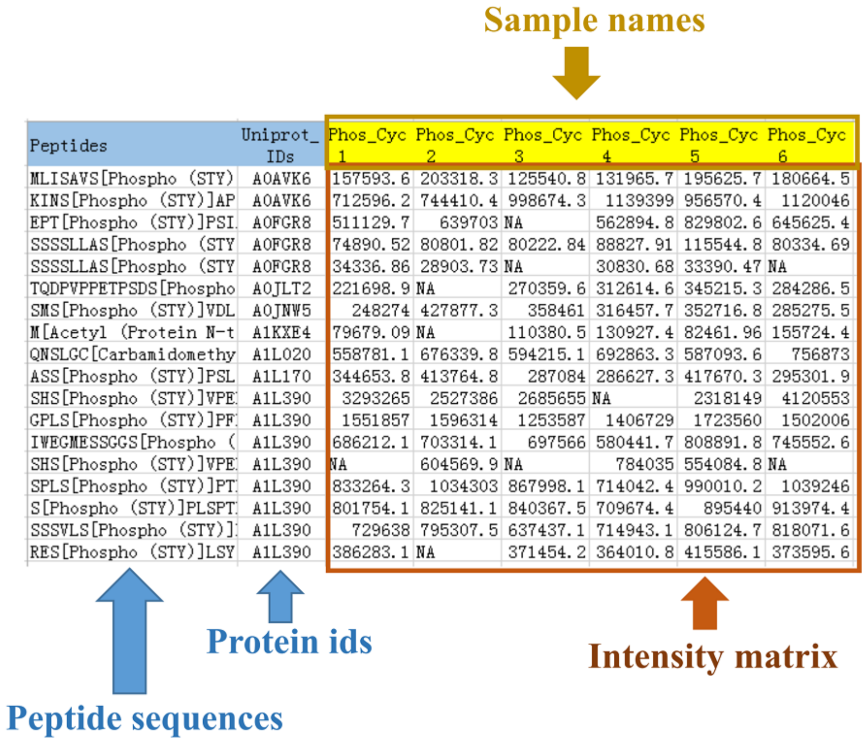

Peptides	Uniprot_IDs	Phos_Cyc 1	Phos_Cyc 2	Phos_Cyc 3	Phos_Cyc 4	Phos_Cyc 5	Phos_Cyc 6
MLISAVS[Phospho (STY)	A0AVK6	157593.6	203318.3	125540.8	131965.7	195625.7	180664.5
KINS[Phospho (STY)]AP	A0AVK6	712596.2	744410.4	998674.3	1139399	956570.4	1120046
EPT[Phospho (STY)]PSI	A0FGR8	511129.7	639703	NA	562894.8	829802.6	645625.4
SSSSLLAS[Phospho (STY	A0FGR8	74890.52	80801.82	80222.84	88827.91	115544.8	80334.69
SSSSLLAS[Phospho (STY	A0FGR8	34336.86	28903.73	NA	30830.68	33390.47	NA
TQDPVPPETPSDS[Phospho	A0JLT2	221698.9	NA	270359.6	312614.6	345215.3	284286.5
SMS[Phospho (STY)]VDL	A0JNW5	248274	427877.3	358461	316457.7	352716.8	285275.5
M[Acetyl (Protein N-t	A1KXE4	79679.09	NA	110380.5	130927.4	82461.96	155724.4
QNSLGC[Carbamidomethy	A1L020	558781.1	676339.8	594215.1	692863.3	587093.6	756873
ASS[Phospho (STY)]PSL	A1L170	344653.8	413764.8	287084	286627.3	417670.3	295301.9
SHS[Phospho (STY)]VPE	A1L390	3293265	2527386	2685655	NA	2318149	4120553
GPLS[Phospho (STY)]PF	A1L390	1551857	1596314	1253587	1406729	1723560	1502006
IWEGMESSGGS[Phospho (	A1L390	686212.1	703314.1	697566	580441.7	808891.8	745552.6
SHS[Phospho (STY)]VPE	A1L390	NA	604569.9	NA	784035	554084.8	NA
SPLS[Phospho (STY)]PT	A1L390	833264.3	1034303	867998.1	714042.4	990010.2	1039246
S[Phospho (STY)]PLSPT	A1L390	801754.1	825141.1	840367.5	709674.4	895440	913974.4
SSSVLS[Phospho (STY)]	A1L390	729638	795307.5	637437.1	714943.1	806124.7	818071.6
RES[Phospho (STY)]LSY	A1L390	386283.1	NA	371454.2	364010.8	415586.1	373595.6

1.1.4 Expression data with protein ids

In this situation, protein ids are provided in the first two columns of input file. The protein ids here should be UniProt ids. From the second column, peptides/proteins expression intensity or signal abundance in every sample should be listed. The data structure is shown as below:

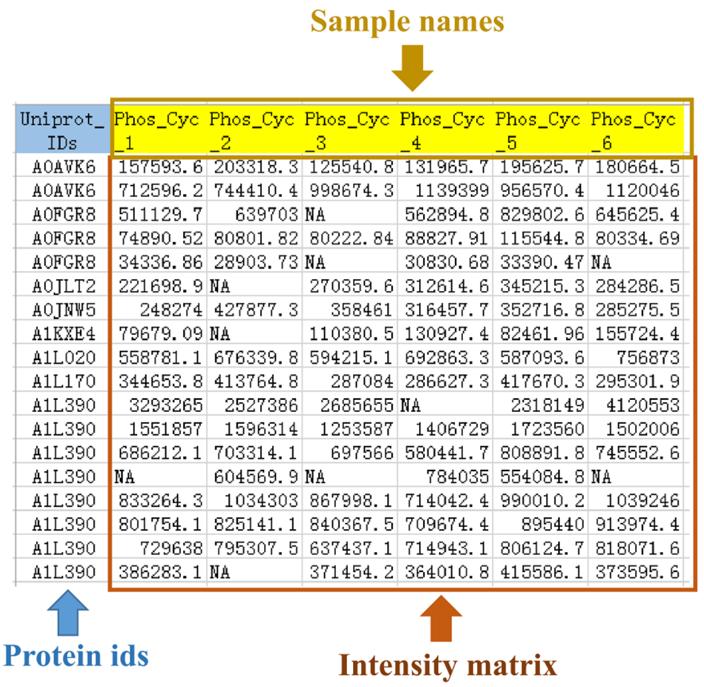

Uniprot_IDs	Phos_Cyc_1	Phos_Cyc_2	Phos_Cyc_3	Phos_Cyc_4	Phos_Cyc_5	Phos_Cyc_6
A0AVK6	157593.6	203318.3	125540.8	131965.7	195625.7	180664.5
A0AVK6	712596.2	744410.4	998674.3	1139399	956570.4	1120046
A0FGR8	511129.7	639703	NA	562894.8	829802.6	645625.4
A0FGR8	74890.52	80801.82	80222.84	88827.91	115544.8	80334.69
A0FGR8	34336.86	28903.73	NA	30830.68	33390.47	NA
A0JLT2	221698.9	NA	270359.6	312614.6	345215.3	284286.5
A0JNW5	248274	427877.3	358461	316457.7	352716.8	285275.5
A1KXE4	79679.09	NA	110380.5	130927.4	82461.96	155724.4
A1L020	558781.1	676339.8	594215.1	692863.3	587093.6	756873
A1L170	344653.8	413764.8	287084	286627.3	417670.3	295301.9
A1L390	3293265	2527386	2685655	NA	2318149	4120553
A1L390	1551857	1596314	1253587	1406729	1723560	1502006
A1L390	686212.1	703314.1	697566	580441.7	808891.8	745552.6
A1L390	NA	604569.9	NA	784035	554084.8	NA
A1L390	833264.3	1034303	867998.1	714042.4	990010.2	1039246
A1L390	801754.1	825141.1	840367.5	709674.4	895440	913974.4
A1L390	729638	795307.5	637437.1	714943.1	806124.7	818071.6
A1L390	386283.1	NA	371454.2	364010.8	415586.1	373595.6

1.1.5 Other kinds of omics data

If users want to use *NAguideR* for other omics data (i.e. genomics, metabolomics), gene/metabolite ids/names should be provided in the first columns of input file. From the second column, genes/metabolites expression intensity or signal abundance in every sample should be listed. The data structure may be shown as below:

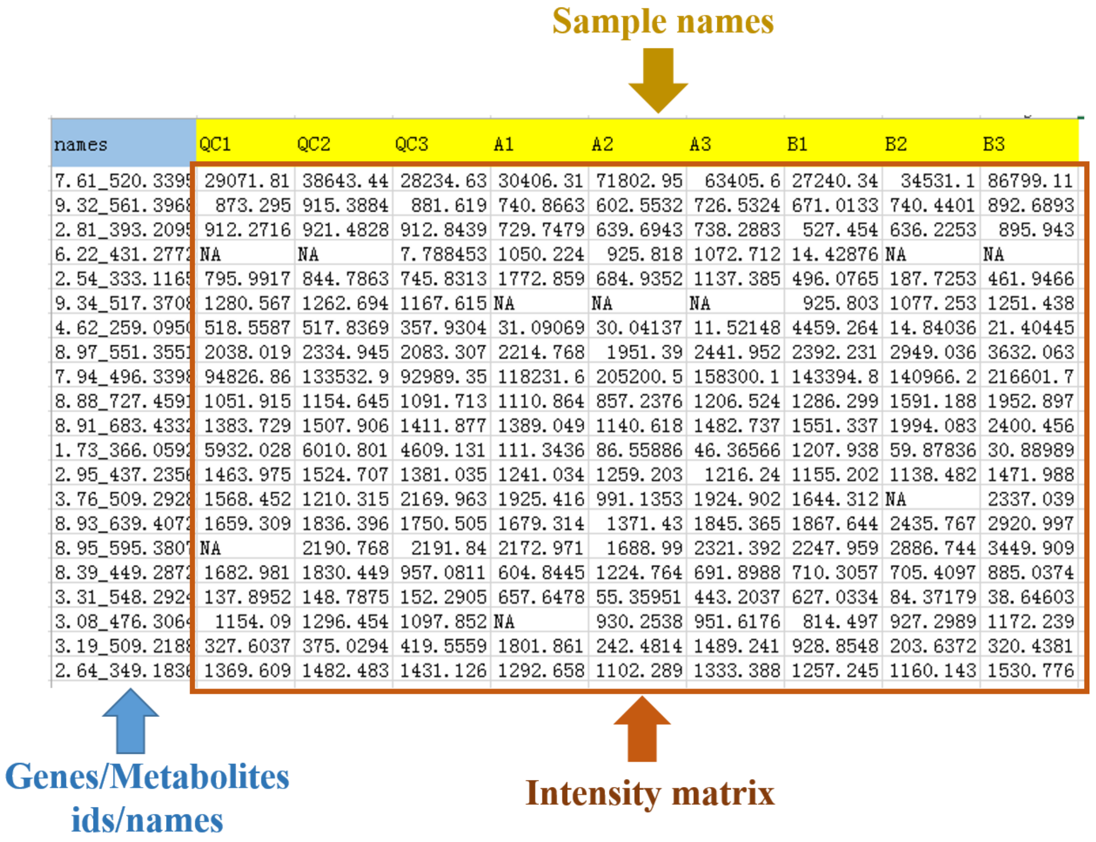

names	QC1	QC2	QC3	A1	A2	A3	B1	B2	B3
7.61_520.3395	29071.81	38643.44	28234.63	30406.31	71802.95	63405.6	27240.34	34531.1	86799.11
9.32_561.3968	873.295	915.3884	881.619	740.8663	602.5532	726.5324	671.0133	740.4401	892.6893
2.81_393.2095	912.2716	921.4828	912.8439	729.7479	639.6943	738.2883	527.454	636.2253	895.943
6.22_431.2772	NA	NA	7.788453	1050.224	925.818	1072.712	14.42876	NA	NA
2.54_333.1165	795.9917	844.7863	745.8313	1772.859	684.9352	1137.385	496.0765	187.7253	461.9466
9.34_517.3708	1280.567	1262.694	1167.615	NA	NA	NA	925.803	1077.253	1251.438
4.62_259.0950	518.5587	517.8369	357.9304	31.09069	30.04137	11.52148	4459.264	14.84036	21.40445
8.97_551.3551	2038.019	2334.945	2083.307	2214.768	1951.39	2441.952	2392.231	2949.036	3632.063
7.94_496.3398	94826.86	133532.9	92989.35	118231.6	205200.5	158300.1	143394.8	140966.2	216601.7
8.88_727.4591	1051.915	1154.645	1091.713	1110.864	857.2376	1206.524	1286.299	1591.188	1952.897
8.91_683.4332	1383.729	1507.906	1411.877	1389.049	1140.618	1482.737	1551.337	1994.083	2400.456
1.73_366.0592	5932.028	6010.801	4609.131	111.3436	86.55886	46.36566	1207.938	59.87836	30.88989
2.95_437.2356	1463.975	1524.707	1381.035	1241.034	1259.203	1216.24	1155.202	1138.482	1471.988
3.76_509.2928	1568.452	1210.315	2169.963	1925.416	991.1353	1924.902	1644.312	NA	2337.039
8.93_639.4072	1659.309	1836.396	1750.505	1679.314	1371.43	1845.365	1867.644	2435.767	2920.997
8.95_595.3807	NA	2190.768	2191.84	2172.971	1688.99	2321.392	2247.959	2886.744	3449.909
8.39_449.2872	1682.981	1830.449	957.0811	604.8445	1224.764	691.8988	710.3057	705.4097	885.0374
3.31_548.2924	137.8952	148.7875	152.2905	657.6478	55.35951	443.2037	627.0334	84.37179	38.64603
3.08_476.3064	1154.09	1296.454	1097.852	NA	930.2538	951.6176	814.497	927.2989	1172.239
3.19_509.2188	327.6037	375.0294	419.5559	1801.861	242.4814	1489.241	928.8548	203.6372	320.4381
2.64_349.1836	1369.609	1482.483	1431.126	1292.658	1102.289	1333.388	1257.245	1160.143	1530.776

1.2 Samples information data

Sample information here means that users should provide sample group identity information. This information could e.g., enable filtration strategy for different group respectively in a later step (see below). The sample names are in the first column and their orders are same as those in the expression data. Group information is in the second column. The data structure is shown as below:

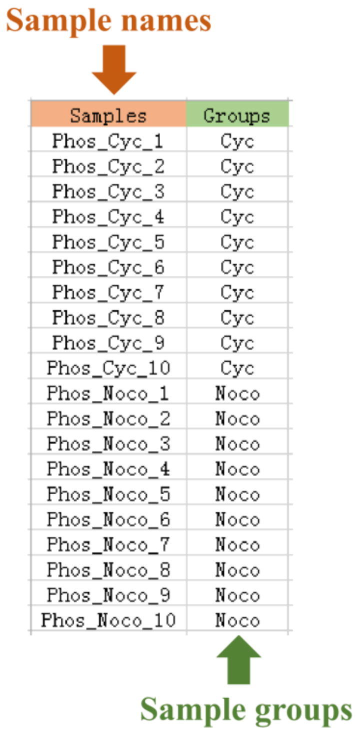

Samples	Groups
Phos_Cyc_1	Cyc
Phos_Cyc_2	Cyc
Phos_Cyc_3	Cyc
Phos_Cyc_4	Cyc
Phos_Cyc_5	Cyc
Phos_Cyc_6	Cyc
Phos_Cyc_7	Cyc
Phos_Cyc_8	Cyc
Phos_Cyc_9	Cyc
Phos_Cyc_10	Cyc
Phos_Noco_1	Noco
Phos_Noco_2	Noco
Phos_Noco_3	Noco
Phos_Noco_4	Noco
Phos_Noco_5	Noco
Phos_Noco_6	Noco
Phos_Noco_7	Noco
Phos_Noco_8	Noco
Phos_Noco_9	Noco
Phos_Noco_10	Noco

1.3 Download example datasets

If users want to download the example datasets to their own computer and check the data format locally, they can download them from here:

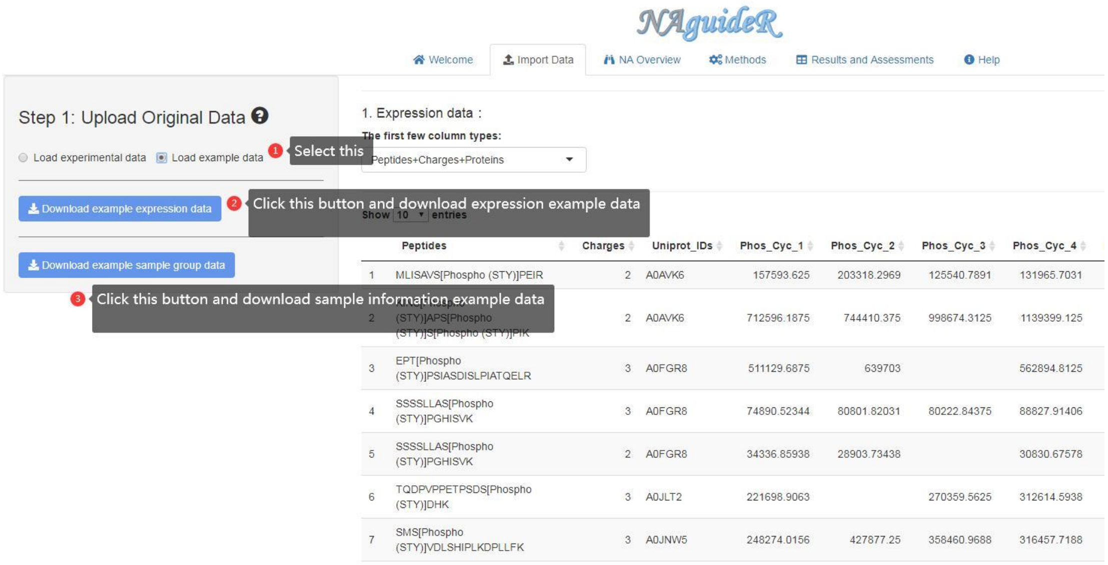

First, select "Load example data" and the example data will be shown on the right panel interactively. Users can visually observe what the data looks like.

Second, users can download the example data (expression data and sample information data) by clicking the corresponding button. The data are saved as .csv format and users can open them in other software, such as Excel.

2. Import Data

This is the first step, in which users should upload data here or load the example data with the above data formats. By default, we use the example data to show result of every step.

2.1 Uploading data. When users prepare their data (expression and sample information data set), they can upload these data from here:

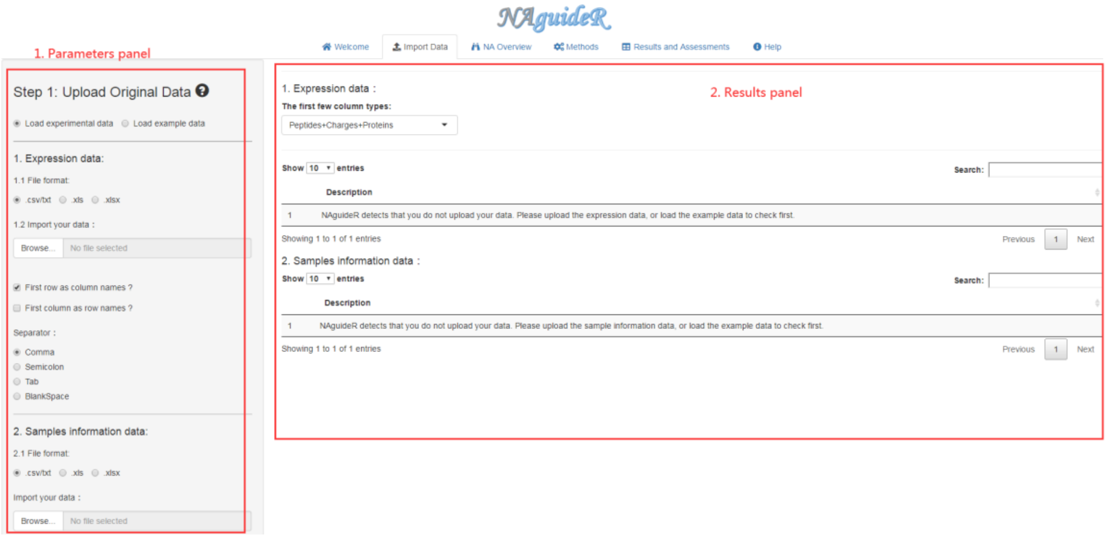

There are two main panels: first, *parameters panel*, users can adjust parameters here; second, *results panel*, many results after users set the parameters will be shown here and users can also download these results.

In the *parameters panel* of “Import Data”, there are two choices for users:

a. Load experimental data. When users choose this option, they can upload their own data here. Users should select the right format based on their data and then click “Browse” button to import the data;

First row as column names: this means whether the first row is column names. If true, you should choose this parameter.

First column as row names: this means whether the first column is row names. If true, you should choose this parameter.

b. Load example data. As described in part 1.3, users can choose this option and download the example data to check them locally.

In the *results panel* of “Import Data”, if users don’t upload their data, here will show “*NAguideR* detects that you did not upload your data. Please upload the expression data (or sample information data), or load the example data to check first” to warn users.

Before uploading expression data, users should also recognize which type their data belongs to and choose the right parameter by adjusting the “*The first few column types*”. The instruction of the column types can be found above (*Data Preparation* part).

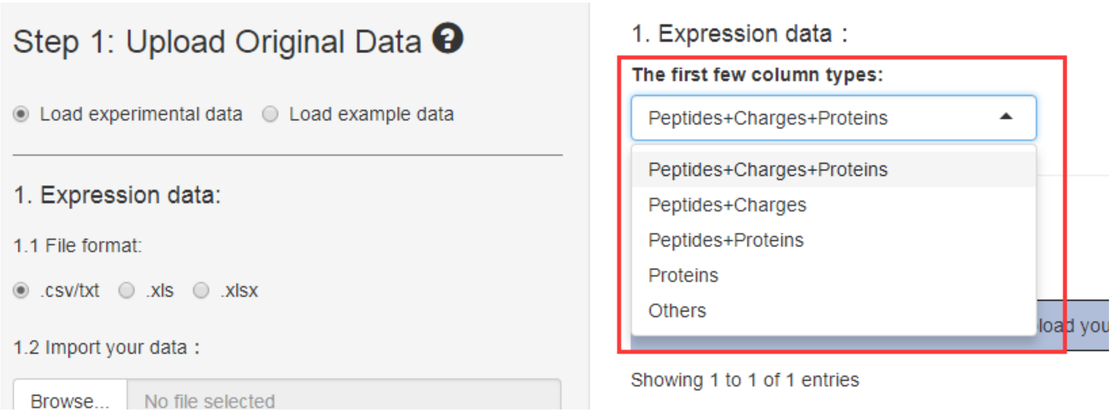
Step 1: Upload Original Data
Load experimental data
Load example data
1. Expression data:
1.1 File format:
.csv/txt
.xls
.xlsx
1.2 Import your data：
Browse...
No file selected
1. Expression data：
The first few column types:
Peptides+Charges+Proteins
Peptides+Charges+Proteins
Peptides+Charges
Peptides+Proteins
Proteins
Others
Showing 1 to 1 of 1 entries

3. NA Overview

Users can check the missing value situation of their own data and filter those data with a high proportion of missing value in this step. Note, "NA" is short for Not Available, which means missing value here (see below).

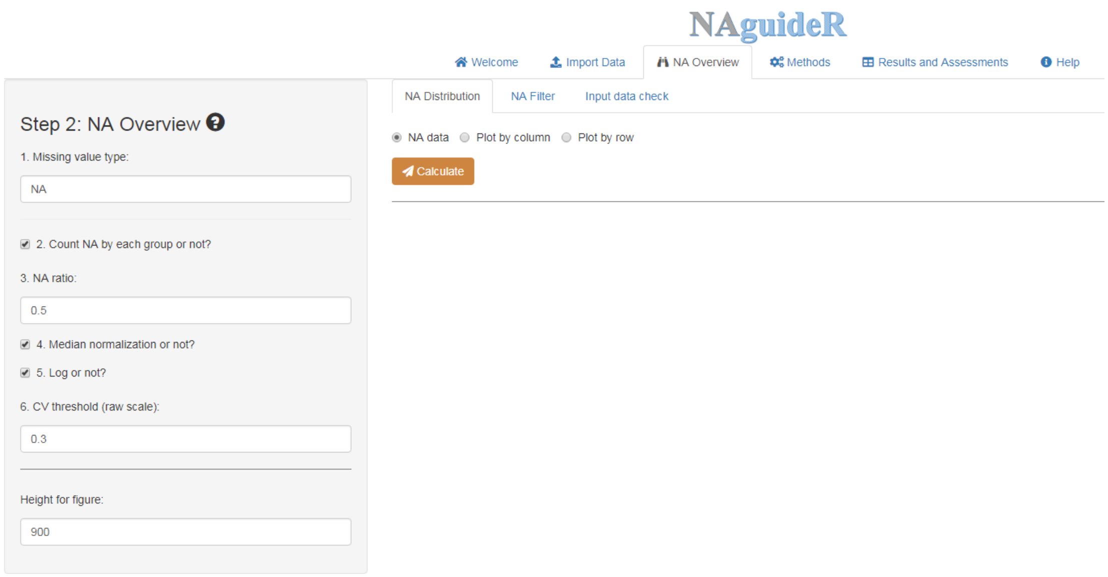

3.1 Parameters

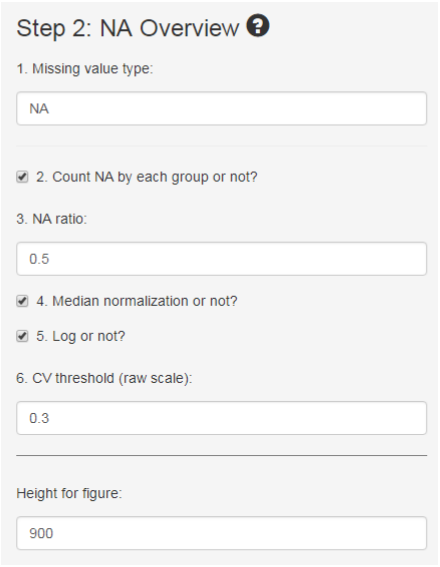

1. Missing value type: what the missing values look like in the expression data, for example, Spectronaut (25,26) software usually export "Filtered" as missing values, so users should change this parameter to "Filtered" if their data contain "Filtered". *NAguideR* will recognize these characters and replace them with NAs. Any other characters indicating a missing value can be similarly defined.

2. Count NA by each group or not: if true, *NAguideR* will count the number of missing values in each group and calculate the NA ratio. Otherwise, it calculates the NA ratio across all groups, for example, as below:

Peptides	Charges	Uniprot_IDs	Phos_Cyc_1	Phos_Cyc_2	Phos_Cyc_3	Phos_Cyc_4	Phos_Cyc_5	Phos_Cyc_6	Phos_Cyc_7	Phos_Cyc_8	Phos_Cyc_9	Phos_Cyc_10	Phos_Noco_1	Phos_Noco_2	Phos_Noco_3	Phos_Noco_4	Phos_Noco_5	Phos_Noco_6	Phos_Noco_7	Phos_Noco_8	Phos_Noco_9	Phos_Noco_10
M[Acetyl (Protein N-te	2	A1KXE4	79679.09	NA	110380.5	130927.4	82461.96	155724.4	113495.3	136404.3	56171.31	98299.7	NA	NA	151027.6	NA	210179.9	182829.7	151424.3	NA	NA	181321.2

There are 2 groups (10 biological replicates in each group) here, if users select this parameter, *NAguideR* will calculate 2 NA ratios for this peptide (first group: 1/10=0.1, second group: 5/10=0.5), otherwise, only one NA ratio: 6/20=0.3.

3. NA ratio: the threshold of NA ratio. Those peptides/proteins with NA ratio above this threshold will be removed.

4. Median normalization or not: if true, *NAguideR* will process median normalization for original data. (Note, *NAguideR* was not designed to perform sophisticated normalization analysis. Any normalized datasets with NA can be accepted for analysis).

5. Log or not: if true, the data will be transformed to the logarithmic scale with base 2.

6. CV threshold (raw scale): the threshold of coefficient of variation. Those peptides/proteins with NA ratio above this threshold will be removed. “raw scale” here means the CV of each peptide/protein is calculate using the data before logarithm transformation.

7. Height for figure: users can adjust the height of figures by changing this parameter.

If users set these parameters well, then click “calculate” button, the results will appear on the right panel.

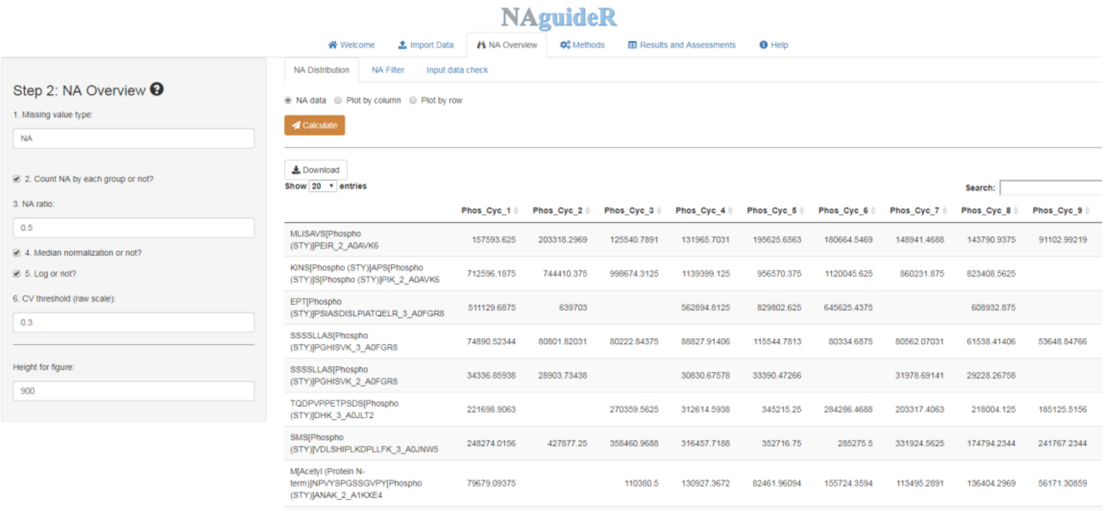

3.2 results of NA overview

a. NA Distribution. This part contains three sub-parts:

a.1 NA data. Here shows the result where the “Missing value type” defined by “NA” will be shown with a blank cell and users can click “Download” button to download this result to their own computer:

Download
Show 20 entries   Search:

	Phos_Cyc_1	Phos_Cyc_2	Phos_Cyc_3	Phos_Cyc_4	Phos_Cyc_5	Phos_Cyc_6	Phos_Cyc_7	Phos_Cyc_8	Phos_Cyc_9	Phos_Cyc_10	Phos_Noco_1	Phos_Noco_2
MLISAVS[Phospho (STY)]PEIR_2_A0AVK6	157593.625	203318.2969	125540.7891	131965.7031	195625.6563	180664.5469	148941.4688	143790.9375	91102.99219	140345.125	59488.09766	92400.46094
KINS[Phospho (STY)]APS[Phospho (STY)]S[Phospho (STY)]PIK_2_A0AVK6	712596.1875	744410.375	998674.3125	1139399.125	956570.375	1120045.625	860231.875	823408.5625		888177.75	509135.625	595305.5
EPT[Phospho (STY)]PSIASDISLPIATQELR_3_A0FGR8	511129.6875	639703		562894.8125	829802.625	645625.4375		608932.875		620510.4375	323346.5313	334969.9063
SSSSLLAS[Phospho (STY)]PGHISVK_3_A0FGR8	74890.52344	80801.82031	80222.84375	88827.91406	115544.7813	80334.6875	80562.07031	61538.41406	53648.84766	65030.57031	516738.8125	782993.875
SSSSLLAS[Phospho (STY)]PGHISVK_2_A0FGR8	34336.85938	28903.73438		30830.67578	33390.47266		31978.69141	29228.26758		26532.99219	333476.9688	297875.2188
TQDPVPPETPSDS[Phospho (STY)]DHK_3_A0JLT2	221698.9063		270359.5625	312614.5938	345215.25	284286.4688	203317.4063	218004.125	185125.5156	245305	81982.05469	81776.67188
SMS[Phospho (STY)]VDLSHIPLKDPLLFK_3_A0JNW5	248274.0156	427877.25	358460.9688	316457.7188	352716.75	285275.5	331924.5625	174794.2344	241767.2344	284089.4375	170259.6094	207056.5
M[Acetyl (Protein N-term)]NPVYSPGSSGVPY[Phospho (STY)]ANAK_2_A1KXE4	79679.09375		110380.5	130927.3672	82461.96094	155724.3594	113495.2891	136404.2969	56171.30859	98299.69531		
QNSLGC[Carbamidomethyl (C)]IGEC[Carbamidomethyl (C)]GVDS[Phospho (STY)]GFEAPR_3_A1L020	558781.125	676339.75	594215.0625	692863.25	587093.5625	756873	569292.25	648059.3125		626625.4375	379149.5625	361978.75

a.2 Plot by column. Here shows the result of the NA distribution of every sample.

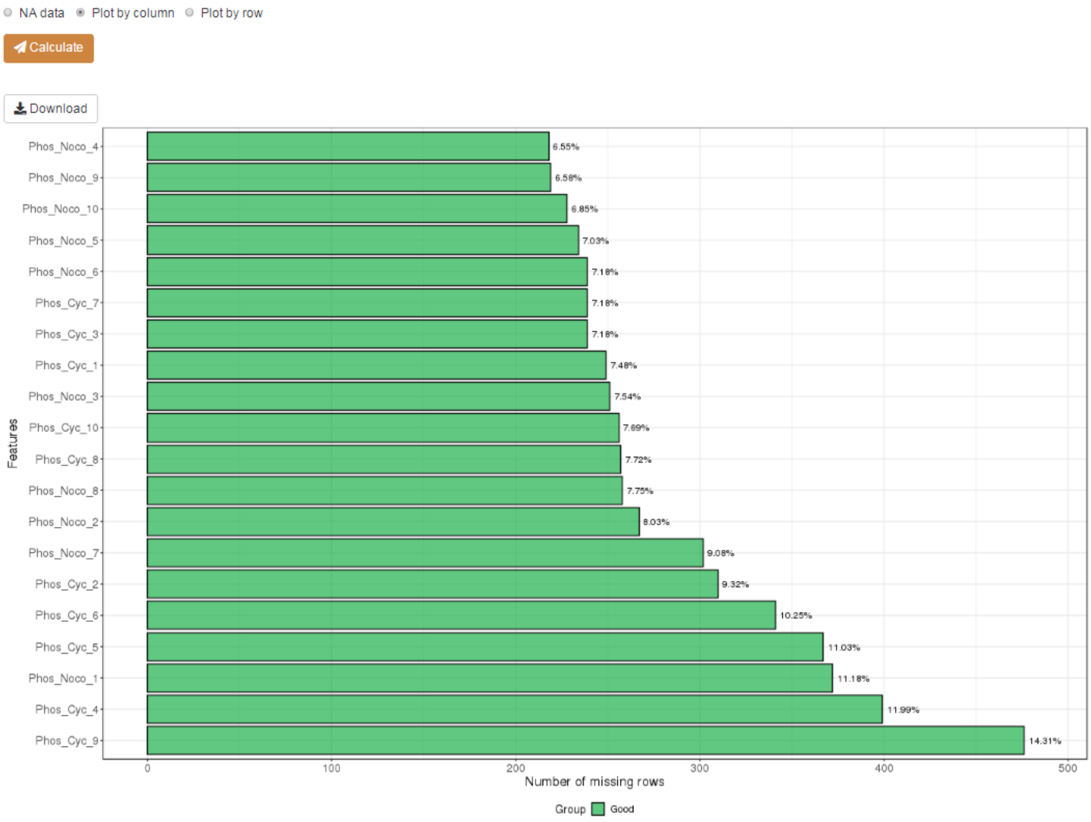

a.2 Plot by row. Here shows the result of the NA distribution of every peptide/protein.

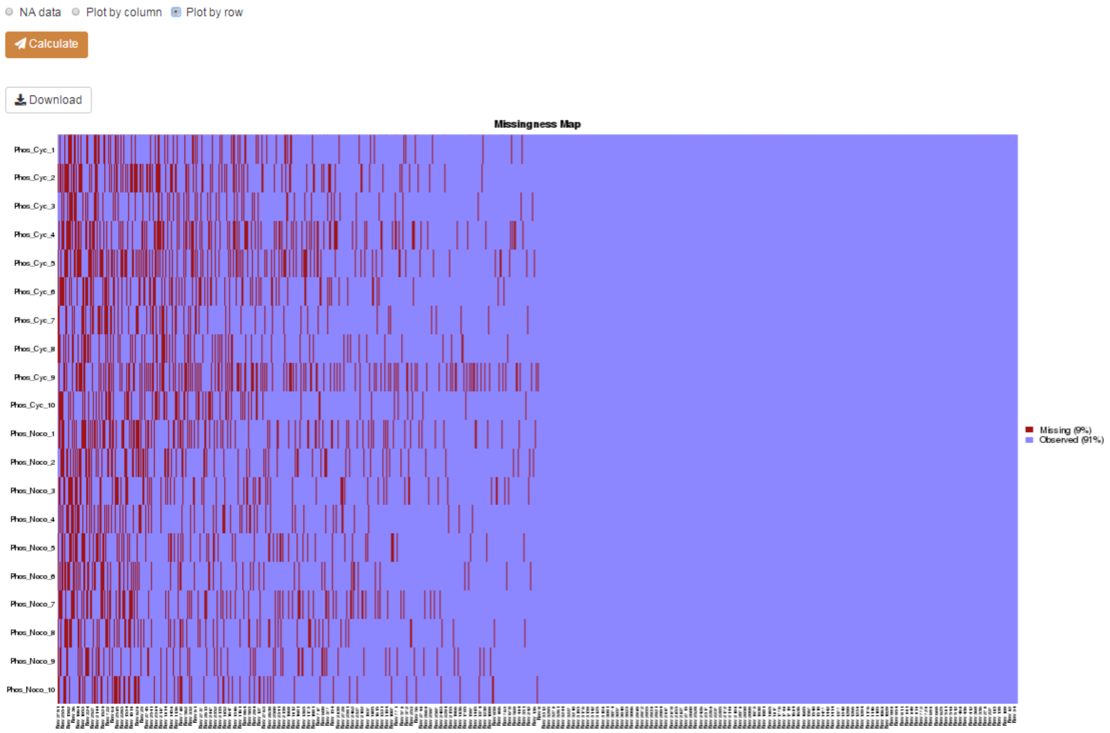

b. NA filter. This part will show the filtered result. That means, on the basis of the preset parameters

(i.e. NA ratio, CV threshold), those objects (peptides/proteins/genes/metabolites) without meeting these requirements would be removed.

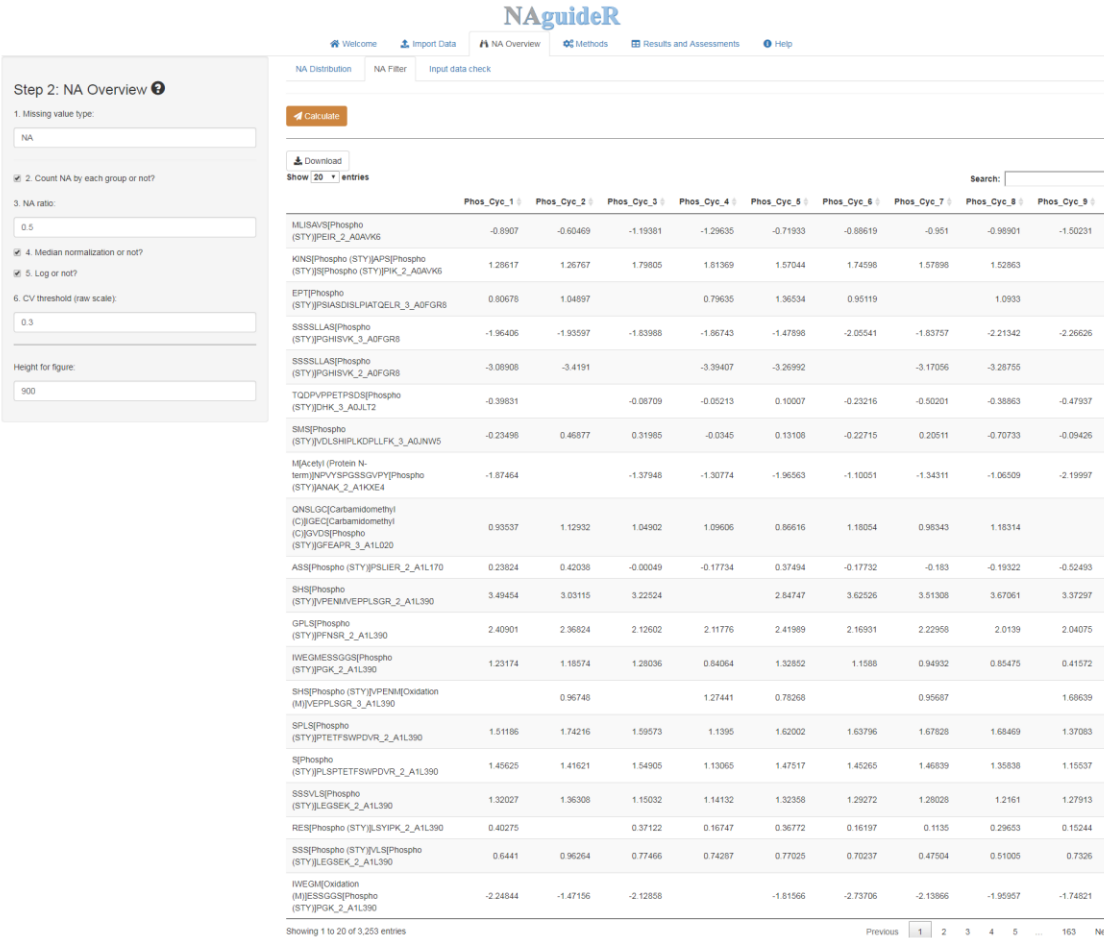

	Phos_Cyc_1	Phos_Cyc_2	Phos_Cyc_3	Phos_Cyc_4	Phos_Cyc_5	Phos_Cyc_6	Phos_Cyc_7	Phos_Cyc_8	Phos_Cyc_9
MLISAVS[Phospho (STY)]PEIR_2_A0AVK6	-0.8907	-0.60469	-1.19381	-1.29635	-0.71933	-0.88619	-0.951	-0.98901	-1.50231
KINS[Phospho (STY)]APS[Phospho (STY)]S[Phospho (STY)]PIK_2_A0AVK6	1.28617	1.26767	1.79805	1.81369	1.57044	1.74598	1.57898	1.52863	
EPT[Phospho (STY)]PSIASDISLPIATQELR_3_A0FGR8	0.80678	1.04897		0.79635	1.36534	0.95119		1.0933	
SSSSLLAS[Phospho (STY)]PGHISVK_3_A0FGR8	-1.96406	-1.93597	-1.83988	-1.86743	-1.47898	-2.05541	-1.83757	-2.21342	-2.26626
SSSSLLAS[Phospho (STY)]PGHISVK_2_A0FGR8	-3.08908	-3.4191		-3.39407	-3.26992		-3.17056	-3.28755	
TQDPVPPETPSDS[Phospho (STY)]DHK_3_A0JLT2	-0.39831		-0.08709	-0.05213	0.10007	-0.23216	-0.50201	-0.38863	-0.47937
SMS[Phospho (STY)]VDLSHIPLKDPLLFK_3_A0JNW5	-0.23498	0.46877	0.31985	-0.0345	0.13108	-0.22715	0.20511	-0.70733	-0.09426
M[Acetyl (Protein N-term)]NPVYSPGSSGVPY[Phospho (STY)]ANAK_2_A1KXE4	-1.87464		-1.37948	-1.30774	-1.96563	-1.10051	-1.34311	-1.06509	-2.19997
QNSLGC[Carbamidomethyl (C)]IGEC[Carbamidomethyl (C)]GVDS[Phospho (STY)]GFEAPR_3_A1L020	0.93537	1.12932	1.04902	1.09606	0.86616	1.18054	0.98343	1.18314	
ASS[Phospho (STY)]PSLIER_2_A1L170	0.23824	0.42038	-0.00049	-0.17734	0.37494	-0.17732	-0.183	-0.19322	-0.52493
SHS[Phospho (STY)]VPENMVEPPLSGR_2_A1L390	3.49454	3.03115	3.22524		2.84747	3.62526	3.51308	3.67061	3.37297
GPLS[Phospho (STY)]PFNSR_2_A1L390	2.40901	2.36824	2.12602	2.11776	2.41989	2.16931	2.22958	2.0139	2.04075
IWEGMESSGGS[Phospho (STY)]PGK_2_A1L390	1.23174	1.18574	1.28036	0.84064	1.32852	1.1588	0.94932	0.85475	0.41572
SHS[Phospho (STY)]VPENM[Oxidation (M)]VEPPLSGR_3_A1L390		0.96748		1.27441	0.78268		0.95687		1.68639
SPLS[Phospho (STY)]PTETFSWPDVR_2_A1L390	1.51186	1.74216	1.59573	1.1395	1.62002	1.63796	1.67828	1.68469	1.37083
S[Phospho (STY)]PLSPTETFSWPDVR_2_A1L390	1.45625	1.41621	1.54905	1.13065	1.47517	1.45265	1.46839	1.35838	1.15537
SSSVLS[Phospho (STY)]LEGSEK_2_A1L390	1.32027	1.36308	1.15032	1.14132	1.32358	1.29272	1.28028	1.2161	1.27913
RES[Phospho (STY)]LSYIPK_2_A1L390	0.40275		0.37122	0.16747	0.36772	0.16197	0.1135	0.29653	0.15244
SSS[Phospho (STY)]VLS[Phospho (STY)]LEGSEK_2_A1L390	0.6441	0.96264	0.77466	0.74287	0.77025	0.70237	0.47504	0.51005	0.7326
IWEGM[Oxidation (M)]ESSGGS[Phospho (STY)]PGK_2_A1L390	-2.24844	-1.47156	-2.12858		-1.81566	-2.73706	-2.13866	-1.95957	-1.74821

c. Input data check. This part will show the checking information as a summary note for input data. By default, if there still remain more than half (>50%) objects in the filtered data, *NAguideR* would think that this is acceptable, and will give users a message like below:

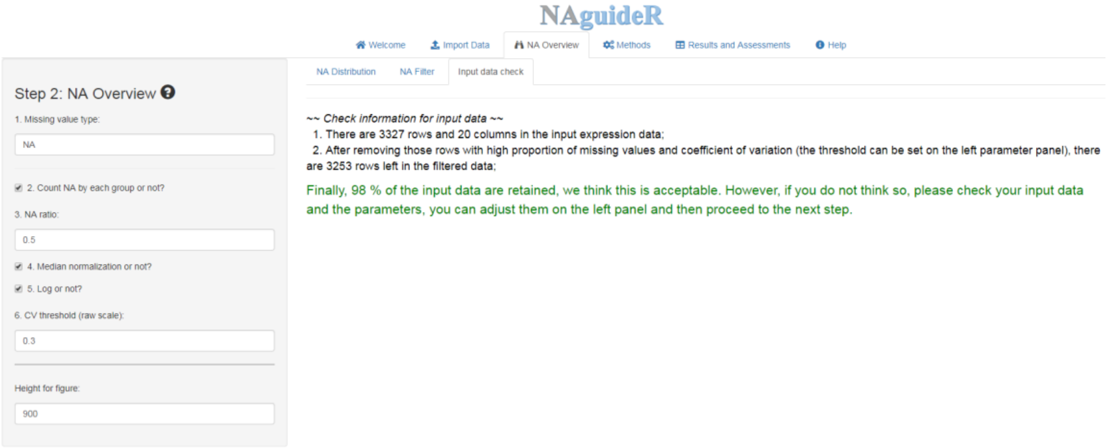

Otherwise, *NAguideR* will give some warnings to users, which means users should pay more attention to their own data and those preset parameters. It is recommended that the users should then make sure that there are no problems before they can proceed to the next step:

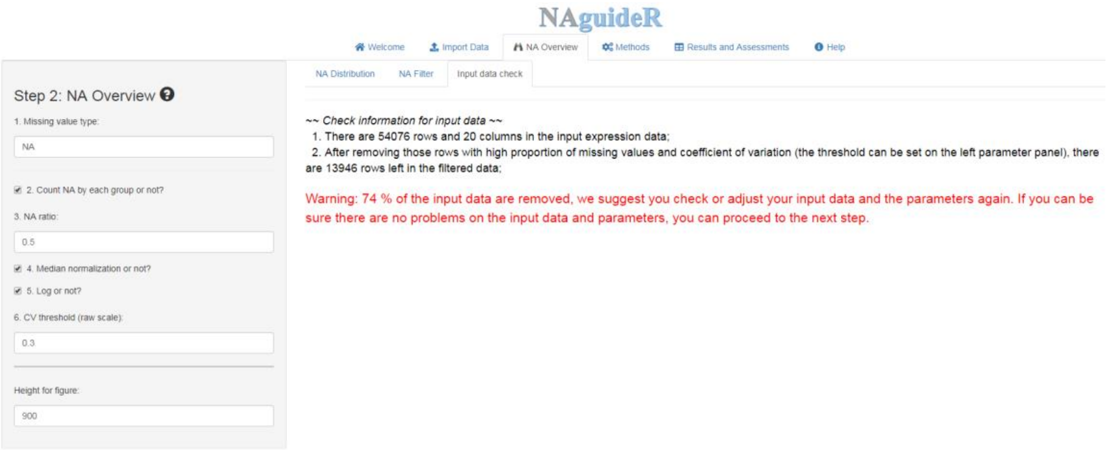
NAguideR
Welcome
Import Data
NA Overview
Methods
Results and Assessments
Help
Step 2: NA Overview
1. Missing value type:
NA
2. Count NA by each group or not?
3. NA ratio:
0.5
4. Median normalization or not?
5. Log or not?
6. CV threshold (raw scale):
0.3
Height for figure:
900
NA Distribution
NA Filter
Input data check
~~ Check information for input data ~~
1. There are 54076 rows and 20 columns in the input expression data;
2. After removing those rows with high proportion of missing values and coefficient of variation (the threshold can be set on the left parameter panel), there are 13946 rows left in the filtered data;
Warning: 74 % of the input data are removed, we suggest you check or adjust your input data and the parameters again. If you can be sure there are no problems on the input data and parameters, you can proceed to the next step.

4. Methods

In this step, users can select any of 23 missing value imputation methods that are currently supported. All methods have been classified into three categories based on their algorithm (Single value approaches, global structure approaches and local similarity approaches). In order to control the running time, we set these fast methods (17 methods) chosen by default. If users choose those slow methods (6 methods), that means the running time will be longer. If users want to try these slow methods, they just need to select the corresponding methods. The detailed information about each method can be found in Table S1. In addition, we also provide the reference for every method just blow each option on the web:

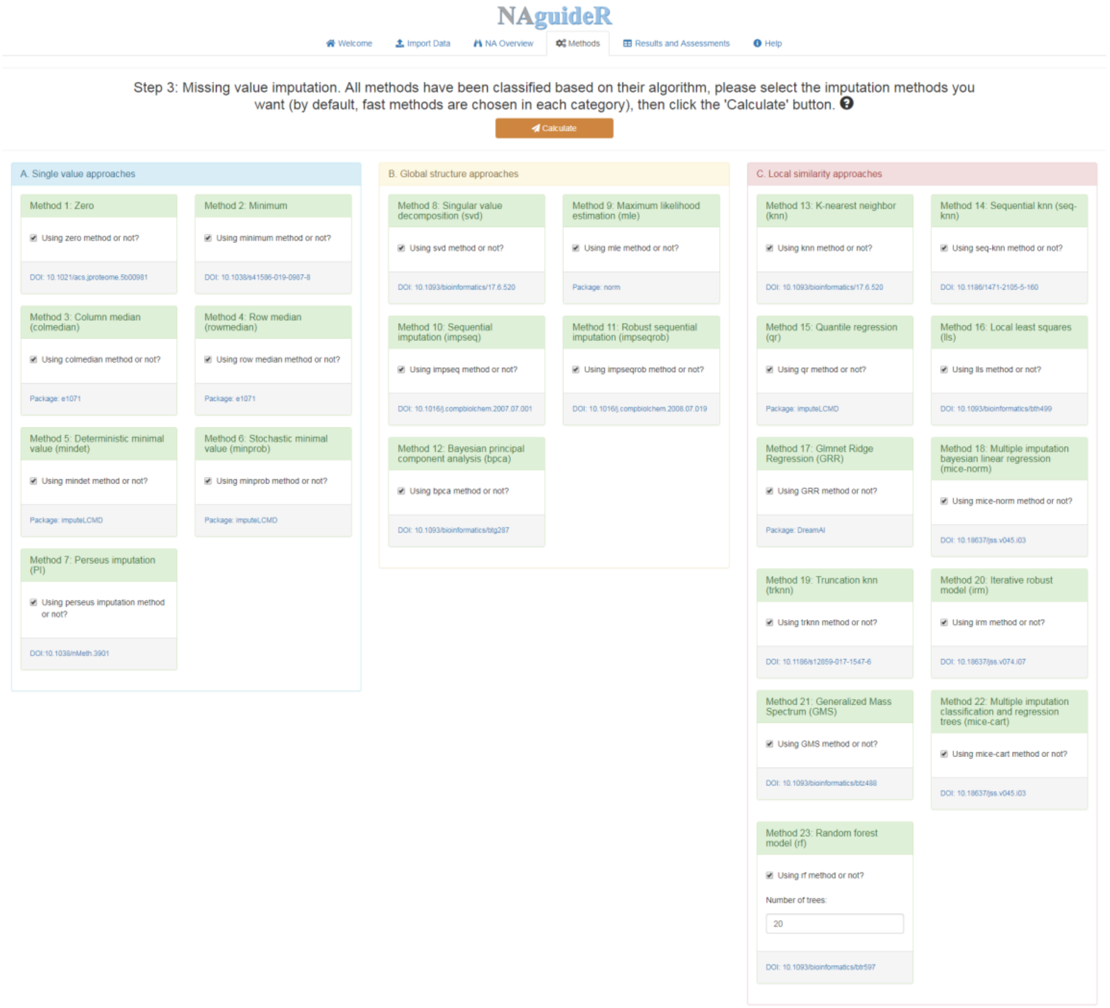

After selecting suitable methods, users need to click 'Calculate' button, and a popup window will be jumped out to show the selected methods, then click 'OK' button and continue:

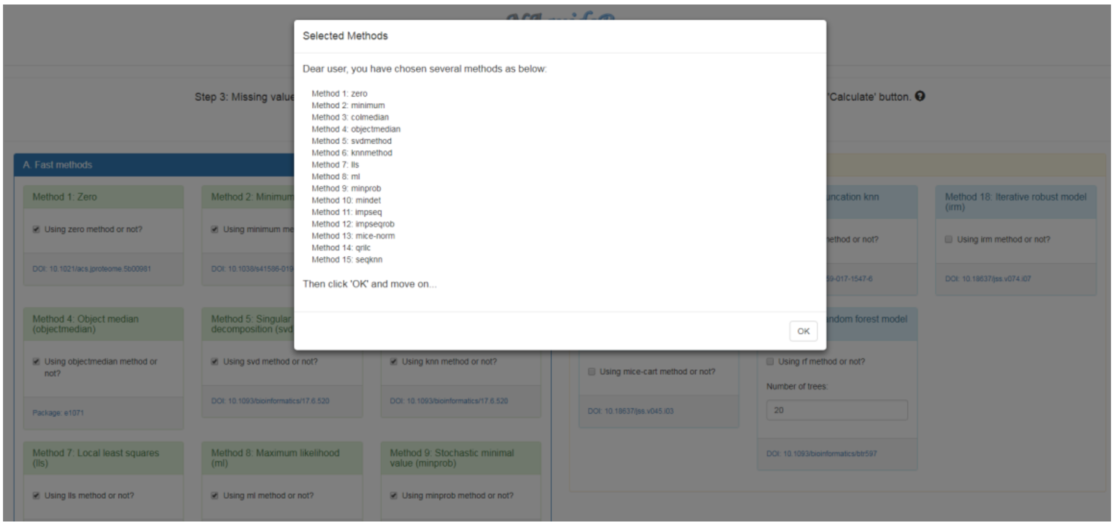
Selected Methods
Dear user, you have chosen several methods as below:
Method 1: zero
Method 2: minimum
Method 3: colmedian
Method 4: objectmedian
Method 5: svdmethod
Method 6: knnmethod
Method 7: lls
Method 8: ml
Method 9: minprob
Method 10: mindet
Method 11: impseq
Method 12: impseqrob
Method 13: mice-norm
Method 14: qrilc
Method 15: seqknn
Then click 'OK' and move on...
OK
A. Fast methods
Method 1: Zero
Using zero method or not?
Method 4: Object median (objectmedian)
Using objectmedian method or not?
Package: e1071
Method 5: Singular decomposition (svd
Using svd method or not?
DOI: 10.1093/bioinformatics/17.6.520
Using knn method or not?
DOI: 10.1093/bioinformatics/17.6.520
Method 7: Local least squares (lls)
Using lls method or not?
Method 8: Maximum likelihood (ml)
Using ml method or not?
Method 9: Stochastic minimal value (minprob)
Using minprob method or not?
Method 18: Iterative robust model (irm)
Using irm method or not?
DOI: 10.18637/jss.v074.i07
Using mice-cart method or not?
DOI: 10.18637/jss.v045.i03
Using rf method or not?
Number of trees:
20
DOI: 10.1093/bioinformatics/btr597
'Calculate' button.

5. Results and Assessments

This step will process missing value imputation and performance evaluation of every method that users select in “Methods” step. Click “Results and Assessments”, *NAguideR* will start to impute these missing value items, a process bar will appear in the bottom right corner to tell users where it goes:

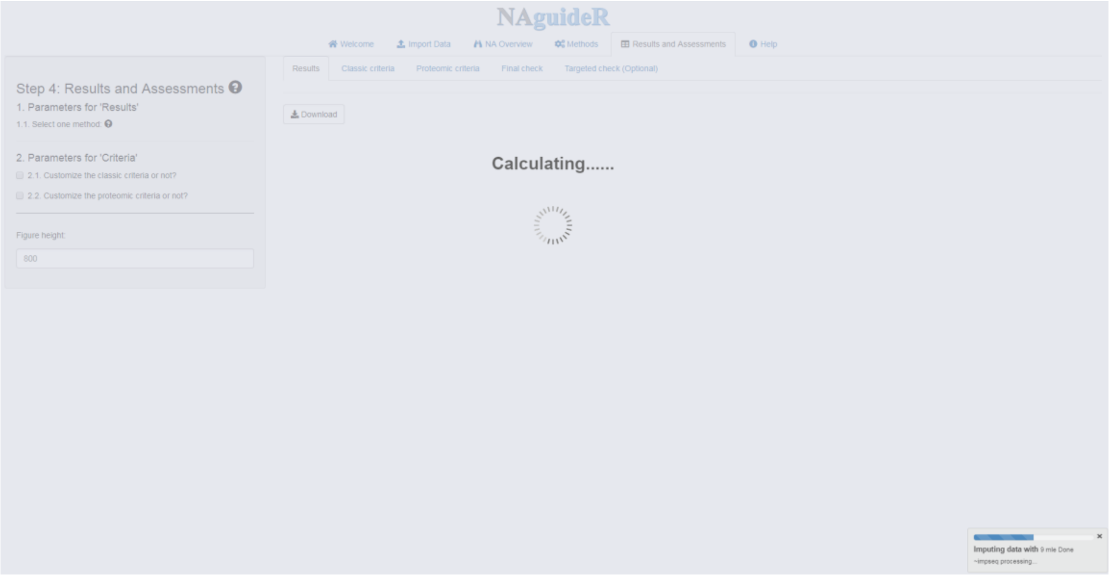

The result from every imputation method will be shown on the “Results” panel:

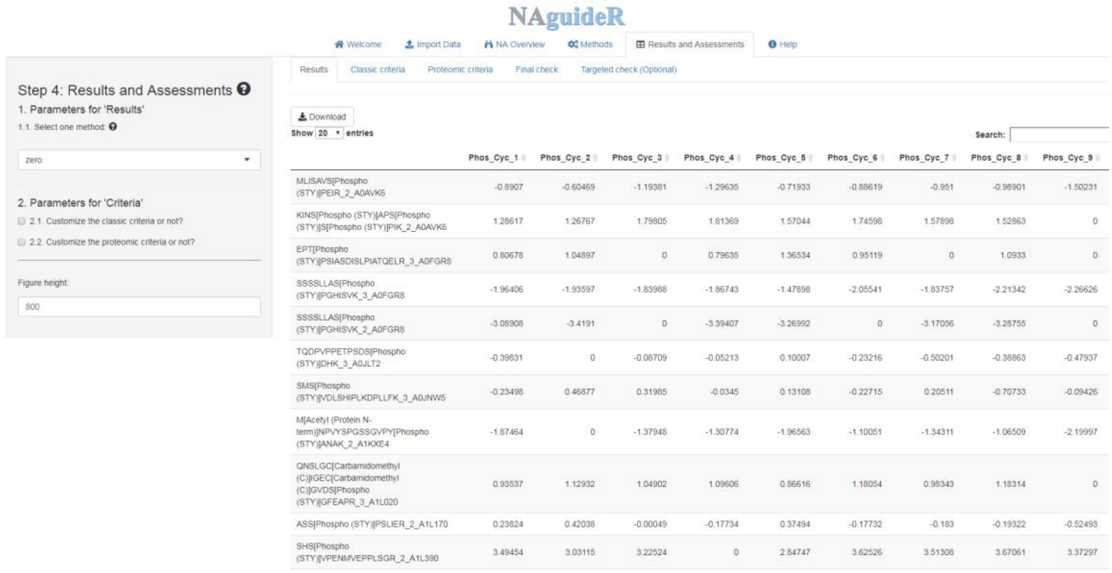

a. Parameters for ‘Results’. Herein users can change the parameter “*Select one method*” on the left panel to check relative result, for example, if users select “zero”, it will show the result derived from zero method:

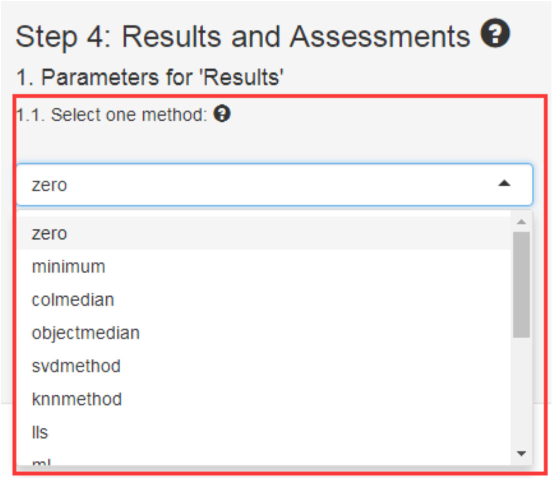

b. *Parameters for 'Criteria'*. Users can customize the criteria and relative weighting for specific experimental designs and aims. By default, these parameters are not selected and all criteria weights are equal.

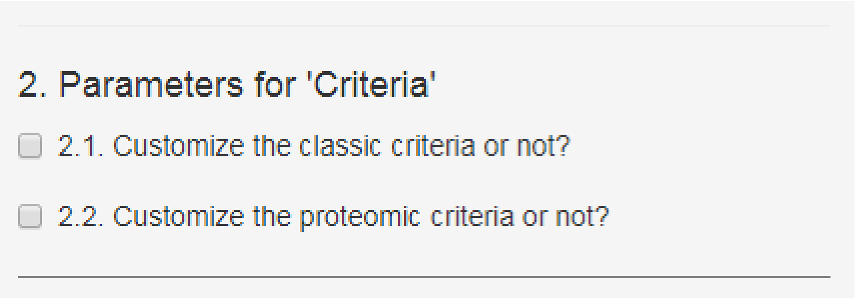

b.1 Customize the classic criteria or not? If true, users can set the classic criteria and relative weight they want, by default, four classic criteria (NRMSE, SOR, ACC_OI, PSS) are chosen and their weights are equal. Please note, the number of criteria and weights should be equal, for example, if users select 'NRMS', 'SOR', and 'PSS', the weights parameter should be type in '1;1;1', which are separated by semicolons, and in this situation, the three criteria weights are all 0.333 (1/3). If users think 'NRMS' should has a higher weight and type in '3;1;1', this means the weight of 'NRMS' is 0.6 (3/5), 'SOR' and 'PSS' is 0.2 (1/5), respectively:

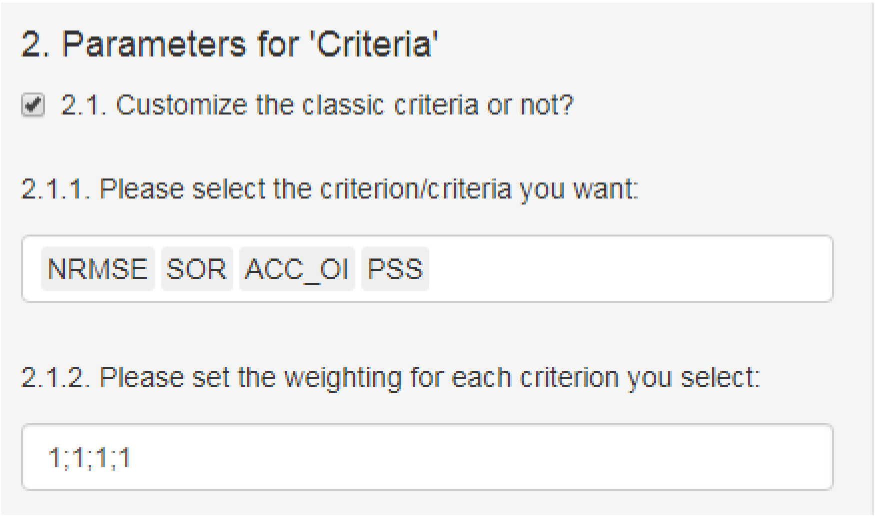

b.2 *Customize the proteomic criteria or not*? If true, users can set the proteomic criteria and relative weight they want, by default, four proteomic criteria (Charge, PepProt, CORUM and PPI) are chosen and their weights are equal. Please also note, the number of criteria and weights should be equal and other descriptions are similar to those for classic criteria as above. Note, the b.1 and b.2 options enable users to customize the criteria and set relative weightings for those specific experimental designs (e.g., a mixture of protein standards being measured in which no in-vivo protein complex formation or interactions expected).

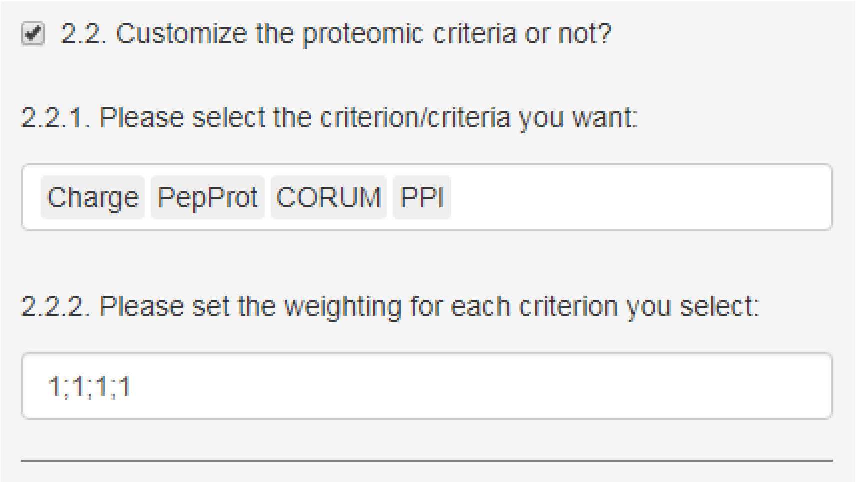

Especially for type ‘Proteins’ dataset (see part 1 above), Charge and PepProt criteria cannot be used (As there are no information about charges and peptides in the data), so users should change the parameters like this if they decide to customize the proteomic criteria:

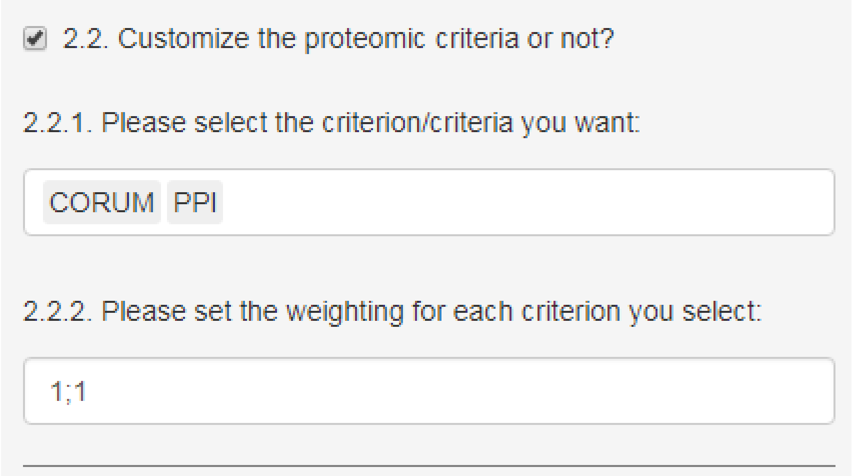

Next, click “Classic criteria” and “Calculate” button. *NAguideR* will assess every method under the four classic criteria:

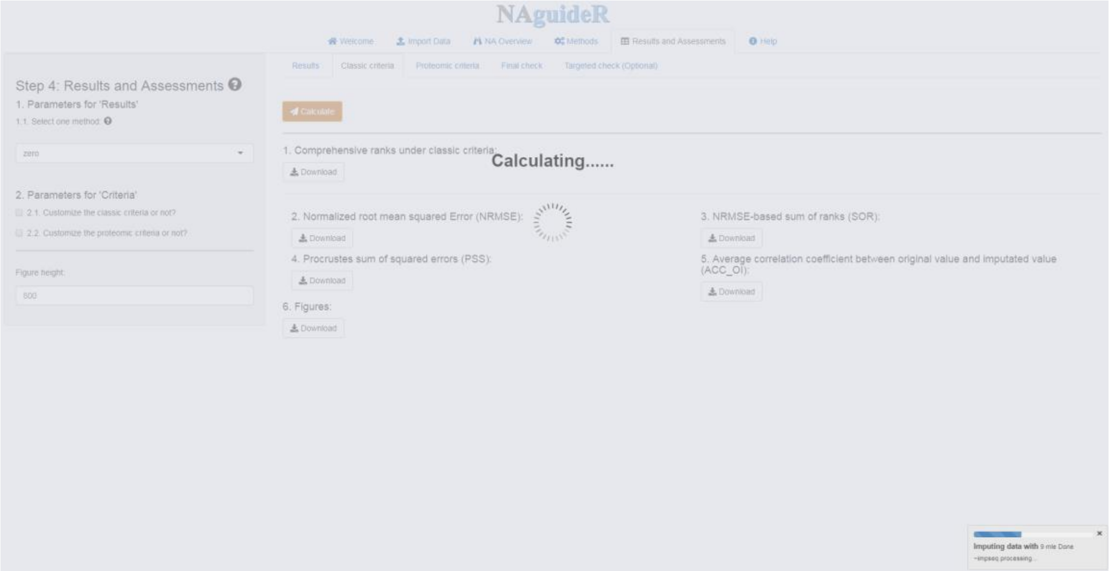

The tables and figures are provided here under the four classic criteria.

1. This table shows the comprehensive ranks of every imputation method. By default, all criteria weights are equal, if users change their weights, and the comprehensive ranks would also change correspondingly based on the new criteria and weights;

2-5, the tables show the scores of every imputation method based on 'Normalized root mean squared Error (NRMSE)', 'NRMSE-based sum of ranks (SOR)', 'Procrustes sum of squared errors (PSS)', and 'Average correlation coefficient between original value and imputed value (ACC_OI)', respectively;

6. Figures here show the normalized scores of every imputation method under the four classic criteria. 'Normalized Values' here means that every score is divided by the corresponding max value.

1. Comprehensive ranks under classic criteria:

Download

Show 20 entries

Search:

	Methods	NRMSE_Rank	SOR_Rank	ACC_OI_Rank	PSS_Rank	Rank_Mean
Method 2	impseq	1	1	1	1	1
Method 3	impseqrob	2	2	2	2	2
Method 13	seqknn	4	3	3	6	4
Method 10	ml	3	6	5	3	4.25
Method 4	knnmethod	5	4	4	5	4.5
Method 5	lls	6	5	6	4	5.25
Method 6	mice-norm	7	7	7	7	7
Method 11	objectmedian	8	8	8	8	8
Method 12	qrilc	9	10	9	11	9.75
Method 14	svdmethod	10	9	10	12	10.25
Method 1	colmedian	11	12	11	10	11
Method 15	zero	12	11	12	9	11
Method 7	mindet	13	13	13	13	13
Method 9	minprob	14	14	14	14	14
Method 8	minimum	15	15	15	15	15

Showing 1 to 15 of 15 entries   Previous 1 Next

2. Normalized root mean squared Error (NRMSE):

Download

Show 20 entries   Search:

	Methods	NRMSE
Method 11	impseq	0.07796
Method 12	impseqrob	0.07814
Method 8	ml	0.10625
Method 15	seqknn	0.11049
Method 6	knnmethod	0.11513
Method 7	lls	0.1237
Method 13	mice-norm	0.16857
Method 4	objectmedian	0.5063
Method 14	qrilc	0.8632
Method 5	svdmethod	0.93162
Method 3	colmedian	1.00393
Method 1	zero	1.08355
Method 10	mindet	2.2209
Method 9	minprob	2.25375
Method 2	minimum	3.28021

Showing 1 to 15 of 15 entries   Previous 1 Next

3. NRMSE-based sum of ranks (SOR):

Download

Show 20 entries   Search:

	Methods	SOR
Method 11	impseq	2122
Method 12	impseqrob	2142
Method 15	seqknn	3536
Method 6	knnmethod	3625
Method 7	lls	3676
Method 8	ml	3696
Method 13	mice-norm	4296
Method 4	objectmedian	6526
Method 5	svdmethod	8030
Method 14	qrilc	8110
Method 1	zero	8406
Method 3	colmedian	8418
Method 10	mindet	10135
Method 9	minprob	10313
Method 2	minimum	11769

Showing 1 to 15 of 15 entries   Previous 1 Next

4. Procrustes sum of squared errors (PSS):

Download

Show 20 entries   Search:

	Methods	PSS
Method 11	impseq	0.00048
Method 12	impseqrob	0.00051
Method 8	ml	0.00064
Method 7	lls	0.00094
Method 6	knnmethod	0.00109
Method 15	seqknn	0.00129
Method 13	mice-norm	0.00556
Method 4	objectmedian	0.02591
Method 1	zero	0.05313
Method 3	colmedian	0.05389
Method 14	qrilc	0.05468
Method 5	svdmethod	0.06779
Method 10	mindet	0.10707
Method 9	minprob	0.10904
Method 2	minimum	0.13141

Showing 1 to 15 of 15 entries   Previous 1 Next

5. Average correlation coefficient between original value and imputated value (ACC_OI):

Download

Show 20 entries   Search:

	Methods	Cor_mean
Method 11	impseq	0.98755
Method 12	impseqrob	0.98748
Method 15	seqknn	0.9757
Method 6	knnmethod	0.975
Method 8	ml	0.97447
Method 7	lls	0.97116
Method 13	mice-norm	0.95947
Method 4	objectmedian	0.8567
Method 14	qrilc	0.69105
Method 5	svdmethod	0.653
Method 3	colmedian	0.63258
Method 1	zero	0.62062
Method 10	mindet	0.37464
Method 9	minprob	0.37038
Method 2	minimum	0.24487

Showing 1 to 15 of 15 entries   Previous 1 Next

6. Figures:

Download

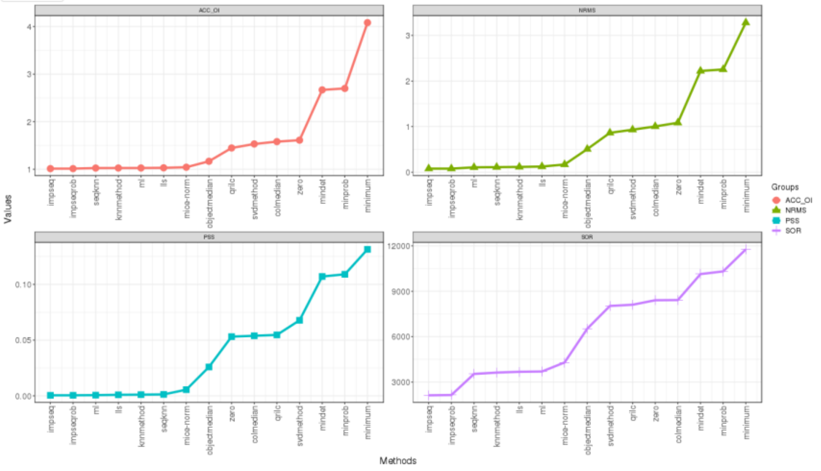

Then click “Proteomic criteria” and “Calculate” button. *NAguideR* will assess every imputation method under the four proteomic criteria:

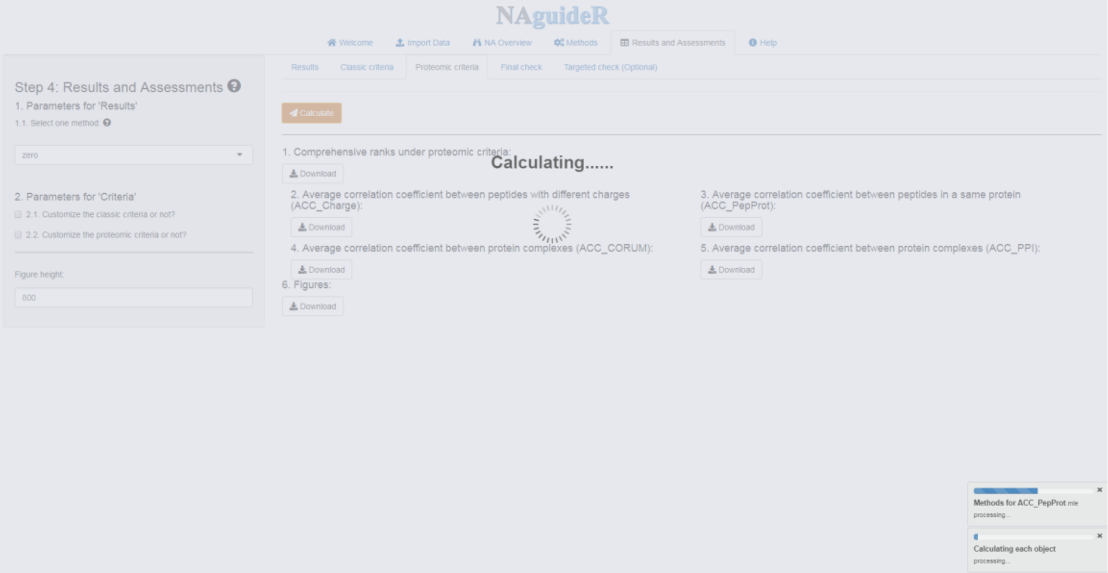

The tables and figures are provided here under the four proteomic criteria.

1. This table shows the comprehensive ranks of every imputation method. By default, all criteria weights are equal, if users change their weights, and the comprehensive ranks would also change correspondingly based on the new criteria and weights;

2-5, the tables show the scores of every imputation method based on 'Average correlation coefficient between peptides with different charges (ACC_Charge)', 'Average correlation coefficient between peptides in a same protein (ACC_PepProt)', 'Average correlation coefficient between protein complexes (ACC_CORUM)', 'Average correlation coefficient between protein complexes (ACC_PPI)', respectively;

6. Figures here show the correlation coefficient distribution of the original values and the imputed values from every imputation method under the four proteomic criteria. Figures will be instantly updated for a particular NA method that can be specified in “*1.1 Select one method*” parameter under Step 4 (left panel). The figure example below shows the results of method “zero”.

1. Comprehensive ranks under proteomic criteria:

Download

Show 20 entries

Search:

	Methods	Charge_Rank	PepProt_Rank	CORUM_Rank	PPI_Rank	Rank_Mean
Method 4	knnmethod	2	1	1	2	1.5
Method 13	seqknn	1	2	4	1	2
Method 2	impseq	3	3	2	3	2.75
Method 3	impseqrob	4	4	3	4	3.75
Method 5	lls	5	5	6	5	5.25
Method 10	ml	6	6	5	6	5.75
Method 6	mice-norm	7	7	7	7	7
Method 11	objectmedian	8	8	8	8	8
Method 12	qrilc	9	9	9	11	9.5
Method 14	svdmethod	10	10	10	9	9.75
Method 1	colmedian	11	11	12	10	11
Method 15	zero	12	12	11	12	11.75
Method 7	mindet	13	13	13	13	13
Method 9	minprob	14	14	14	14	14
Method 8	minimum	15	15	15	15	15

Showing 1 to 15 of 15 entries Previous 1 Next

2. Average correlation coefficient between peptides with different charges (ACC_Charge):

Download

Show 20 entries

Search:

	Methods	ACC_Charge
Method 15	seqknn	0.84803
Method 6	knnmethod	0.84666
Method 11	impseq	0.84525
Method 12	impseqrob	0.84508
Method 7	lls	0.84018
Method 8	ml	0.83723
Method 13	mice-norm	0.82996
Method 4	objectmedian	0.73897
Method 14	qrilc	0.62586
Method 5	svdmethod	0.60933
Method 3	colmedian	0.59157
Method 1	zero	0.58832
Method 10	mindet	0.43458
Method 9	minprob	0.42645
Method 2	minimum	0.35983

Showing 1 to 15 of 15 entries Previous 1 Next

3. Average correlation coefficient between peptides in a same protein (ACC_PepProt):

Download

Show 20 entries

Search:

	Methods	ACC_peppro
Method 6	knnmethod	0.54888
Method 15	seqknn	0.54877
Method 11	impseq	0.54602
Method 12	impseqrob	0.54588
Method 7	lls	0.54151
Method 8	ml	0.54064
Method 13	mice-norm	0.53333
Method 4	objectmedian	0.47951
Method 14	qrilc	0.40258
Method 5	svdmethod	0.38689
Method 3	colmedian	0.37715
Method 1	zero	0.37693
Method 10	mindet	0.27806
Method 9	minprob	0.27274
Method 2	minimum	0.22728

Showing 1 to 15 of 15 entries Previous 1 Next

4. Average correlation coefficient between protein complexes (ACC_CORUM):

Download

Show 20 entries

Search:

	Methods	ACC_CORUM
Method 6	knnmethod	0.30498
Method 11	impseq	0.30475
Method 12	impseqrob	0.30471
Method 15	seqknn	0.30459
Method 8	ml	0.29933
Method 7	lls	0.29666
Method 13	mice-norm	0.29583
Method 4	objectmedian	0.2485
Method 14	qrilc	0.21802
Method 5	svdmethod	0.19725
Method 1	zero	0.19269
Method 3	colmedian	0.18941
Method 10	mindet	0.15264
Method 9	minprob	0.15054
Method 2	minimum	0.127

Showing 1 to 15 of 15 entries Previous 1 Next

5. Average correlation coefficient between protein complexes (ACC_PPI):

Download

Show 20 entries

Search:

	Methods	ACC_PPI
Method 15	seqknn	0.48217
Method 6	knnmethod	0.48201
Method 11	impseq	0.48111
Method 12	impseqrob	0.48108
Method 7	lls	0.4779
Method 8	ml	0.47428
Method 13	mice-norm	0.46884
Method 4	objectmedian	0.41256
Method 5	svdmethod	0.35871
Method 3	colmedian	0.34504
Method 14	qrilc	0.33687
Method 1	zero	0.33539
Method 10	mindet	0.22936
Method 9	minprob	0.22714
Method 2	minimum	0.18582

Showing 1 to 15 of 15 entries Previous 1 Next

6. Figures:

Download

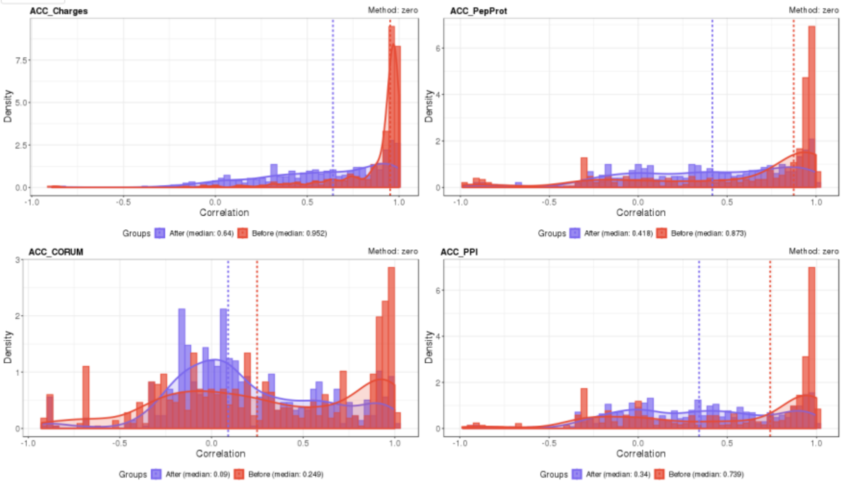

Next, click ‘Final check’ for checking final imputation results as a summary note. *NAguideR* will re-check those scores based on every criterion. If everything is acceptable (see below), *NAguideR* will show a message like:

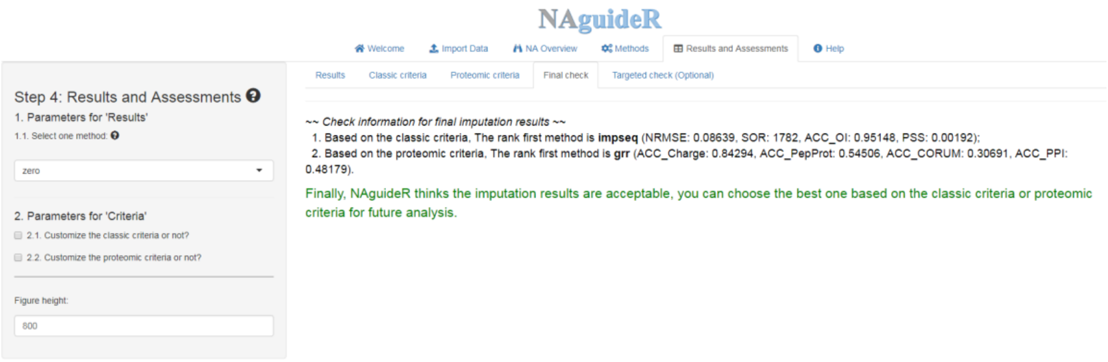

Here, *NAguideR* performs a simple check to report if there is any big difference among these imputation methods under more than half of the criteria (by default, *NAguideR* check the fold change between the maximum score and the minimum score for each criterion, if the fold change is below 2, a fact suggesting that no big difference under the corresponding criterion, i.e., that *NAguideR* cannot provide a significantly discriminant guidance on NA method selection), *NAguideR* will give some warnings and possible solutions for users to review/re-calculate these imputation results:

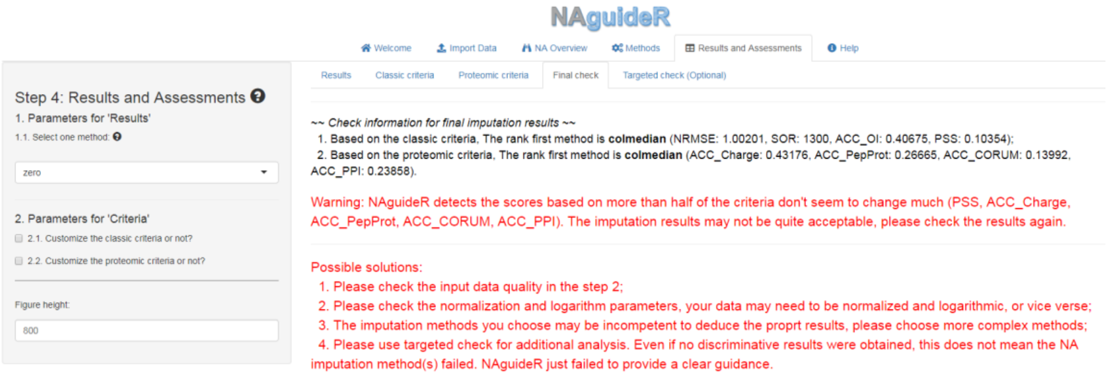

Last but not least, *NaguideR* implements one optional function, ‘Targeted check’, which is designed for many biologists with specific experimental aims. For example, this feature conveniently allows users to directly visualize the results of a particular peptide or protein item (i.e., spiked-in standard peptides, proteins, or known housekeeping proteins like beta-actin, etc.). Therefore, by following their experimental design, they can type in the peptide sequence or protein id in the text area and click the ‘Check’ button.

Then, *NAguideR* will locate this peptide or protein id in the input and resultant matrix (if the peptide/protein is not listed in the user’s input data, it will give a message, “Target protein/peptide not found. Please make sure the item is included in the input table”, example 1 as below). If the peptide/protein is searched, *NAguideR* will show the results before and after imputation by using bar plots and provide a note “Target protein/peptide was missed in N=X samples among all N=Y samples” (example 2 as below). This plot should help the users to inspect results following their particular experimental design. If the target protein/peptide is quantified without the need of NA imputation,

NAguideR will still display the bar plots and provide a note, “Target protein/peptide was not missed in any sample” (example 3 as below).

Example 1 (Target protein/peptide not found. Please make sure the item is included in the input table):

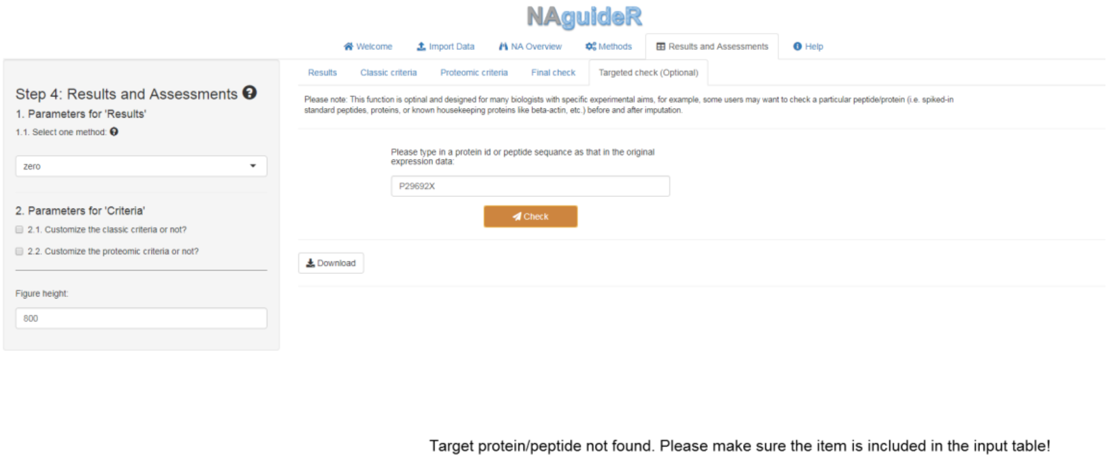

Example 2 (Target protein/peptide was missed in N=10 samples among all N=20 samples):

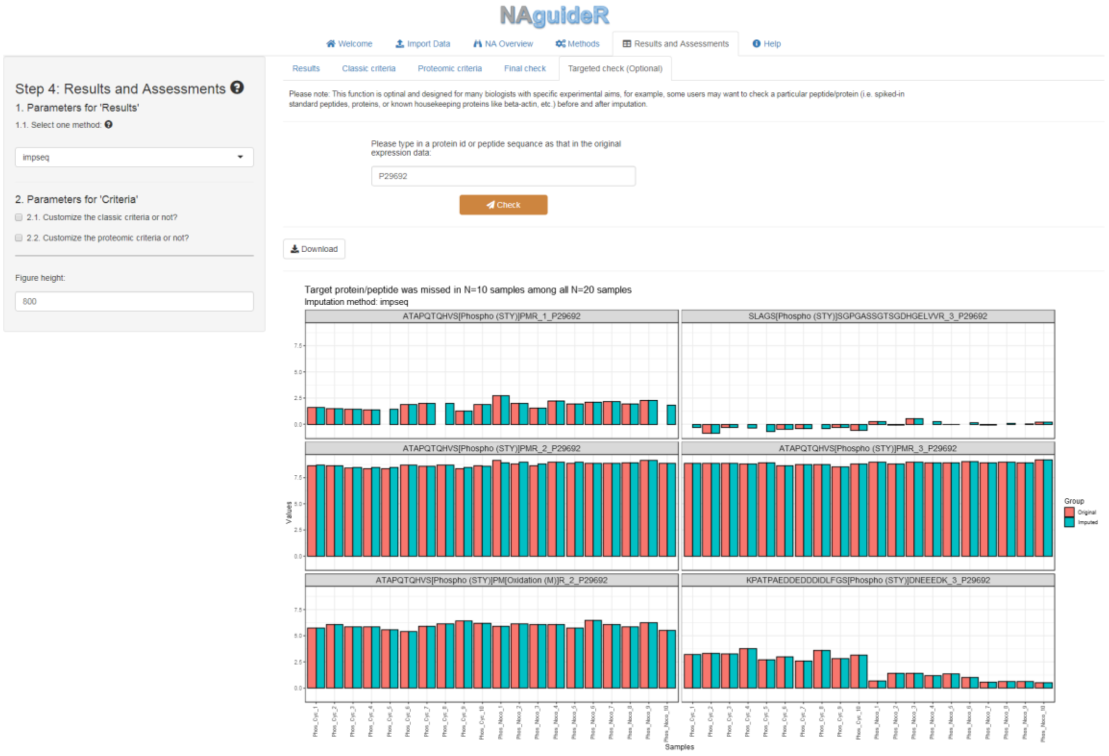

Example 3 (Target protein/peptide was not missed in any sample):

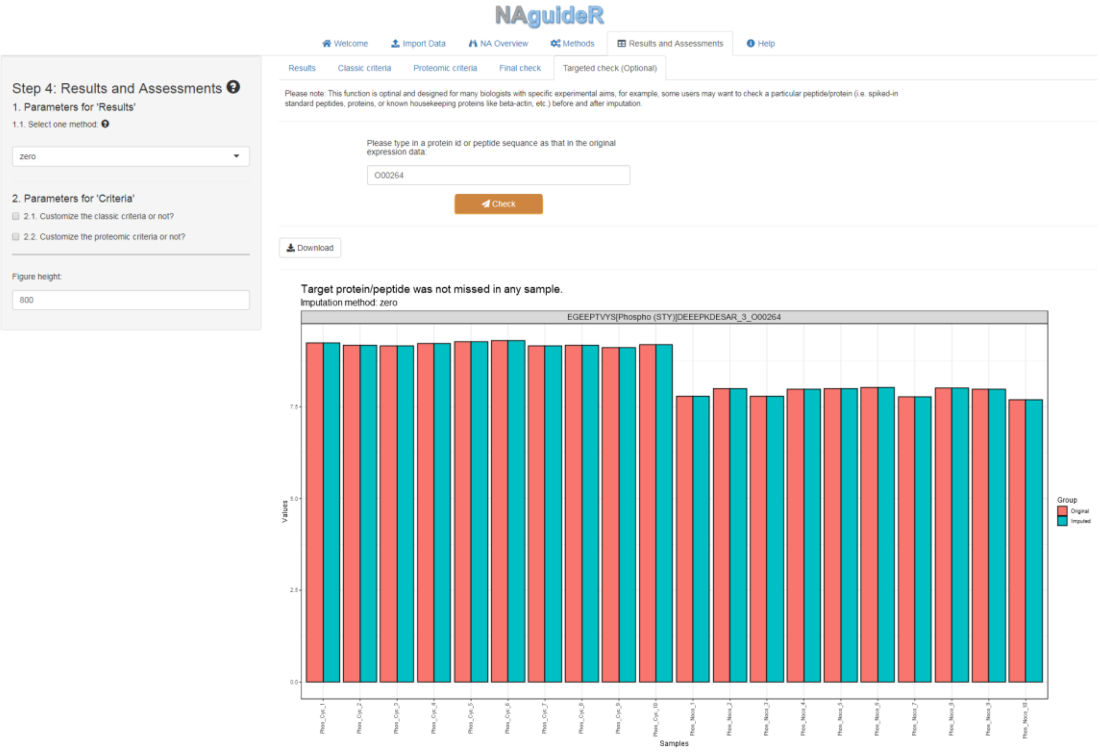
NAguideR
Welcome
Import Data
NA Overview
Methods
Results and Assessments
Help
Step 4: Results and Assessments
1. Parameters for 'Results'
1.1. Select one method:
zero
2. Parameters for 'Criteria'
2.1. Customize the classic criteria or not?
2.2. Customize the proteomic criteria or not?
Figure height:
800
Results
Classic criteria
Proteomic criteria
Final check
Targeted check (Optional)
Please note: This function is optinal and designed for many biologists with specific experimental aims, for example, some users may want to check a particular peptide/protein (i.e. spiked-in standard peptides, proteins, or known housekeeping proteins like beta-actin, etc.) before and after imputation.
Please type in a protein id or peptide sequance as that in the original expression data:
O00264
Check
Download
Target protein/peptide was not missed in any sample.
Imputation method: zero
EGEEPTVYS[Phospho (STY)]DEEEPKDESAR_3_O00264
Values
Samples
Group
Original
Imputed

6. Help

This part provides brief introductions and operation manual about *NAguideR* for users to quickly learn this tool and start to use this tool.

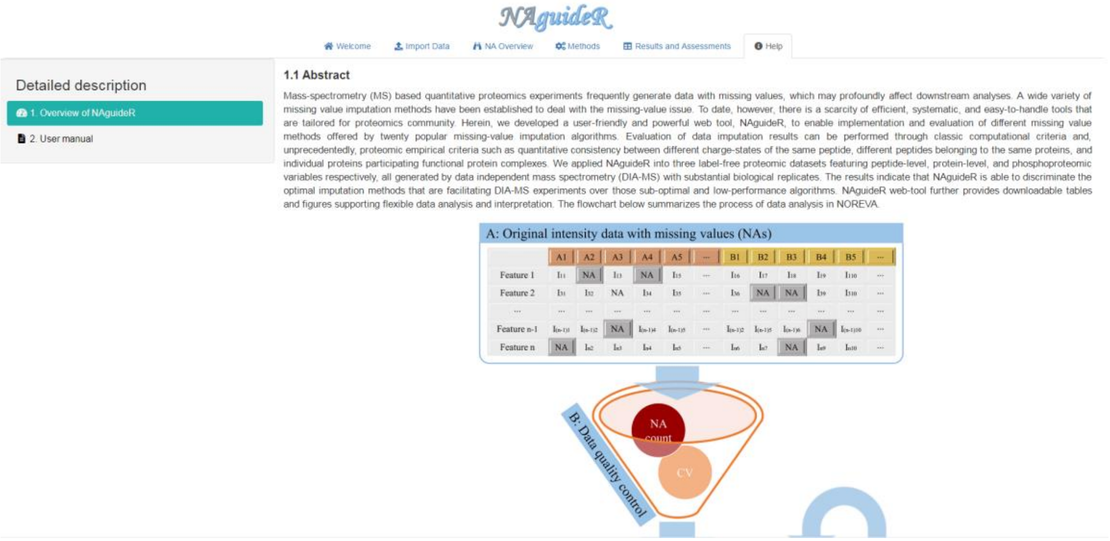

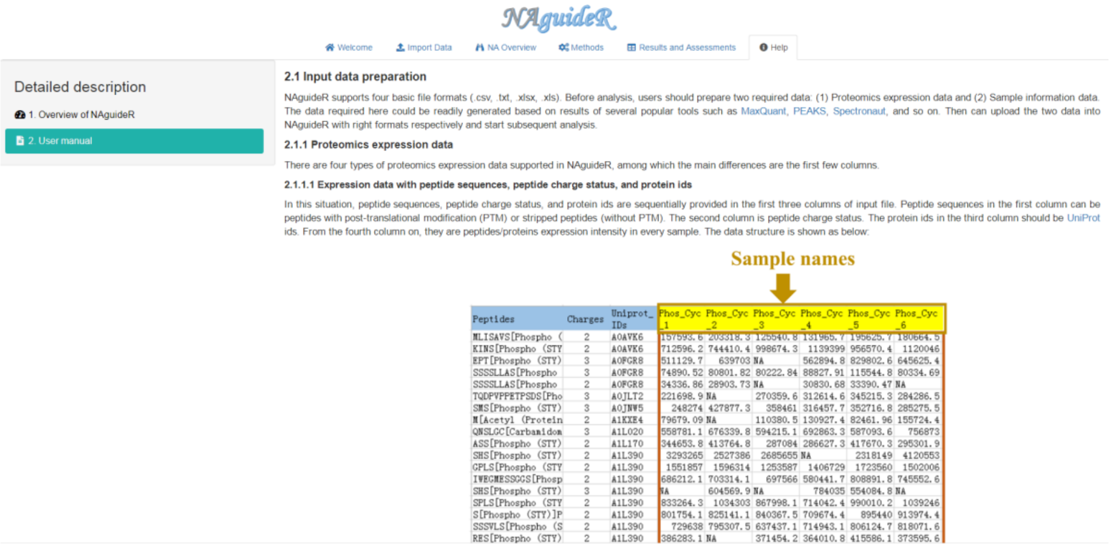

7. How to run this tool locally?

NAguideR is an open source software for non-commercial use and all codes can be obtained on our GitHub: https://github.com/wangshisheng/NAguideR. If users want to run *NAguideR* on their own computer, they should operate as below:

As this tool was developed with R, you may:

a) Install R. You can download R from here: https://www.r-project.org/.

b) Install RStudio. (Recommendatory but not necessary). You can download RStudio from here: https://www.rstudio.com/.

c) Check packages. After installing R and RStudio, you should check whether you have installed these packages (shiny, shinyBS, shinyjs, shinyWidgets, DT, gdata, ggplot2, ggsci, openxlsx, data.table, DT, raster, Metrics, vegan, tidyverse, ggExtra, cowplot, Amelia, e1071, impute, SeqKnn, pcaMethods, norm, imputeLCMD, VIM, rrcovNA, mice, missForest, GMSimpute, DreamAI). You may run the codes below to check them:

```
if(!require(pacman)) install.packages("pacman")
pacman::p_load(shiny, shinyBS, shinyjs, shinyWidgets, DT, gdata, ggplot2, ggsci, openxlsx,
data.table, DT, raster, Metrics, vegan, tidyverse, ggExtra, cowplot, Amelia, e1071, impute,
SeqKnn, pcaMethods, norm, imputeLCMD, VIM, rrcovNA, mice, missForest, GMSimpute,
DreamAI)
```

Please note, you may find the SeqKnn package (https://github.com/cran/SeqKnn) cannot be installed rightly as it has not been updated for a long time. If so, please download this package from here: https://github.com/wangshisheng/NAguideR/blob/master/SeqKnn_1.0.1.tar.gz. Then you can install this separate package locally:

```
setwd('path') #path is where the two packages are.
install.packages("SeqKnn_1.0.1.tar.gz", repos = NULL,type="source")
```

d) Run this tool locally

```
if(!require(NAguideR)) devtools::install_github("wangshisheng/NAguideR")
library(NAguideR)
NAguideR_app()
```

Then NAguideR will be started as below, and the detailed operation about NAguideR can be found in the Supplementary Notes part 1-6:

NAguideR

Welcome | Import Data | NA Overview | Methods | Results and Assessments | Help

~~ Dear Users, Welcome to NAguideR ~~

NAguideR is a web-based tool, which integrates 23 commonly used missing value imputation methods and provides two categories of evaluation criteria (4 classic criteria and 4 proteomic criteria) to assess the imputation performance of various methods. We hope this tool could help scientists impute the missing values systematically and present valuable guidance to select one proper method for their own data. In addition, this tool supports both online access and local installation.

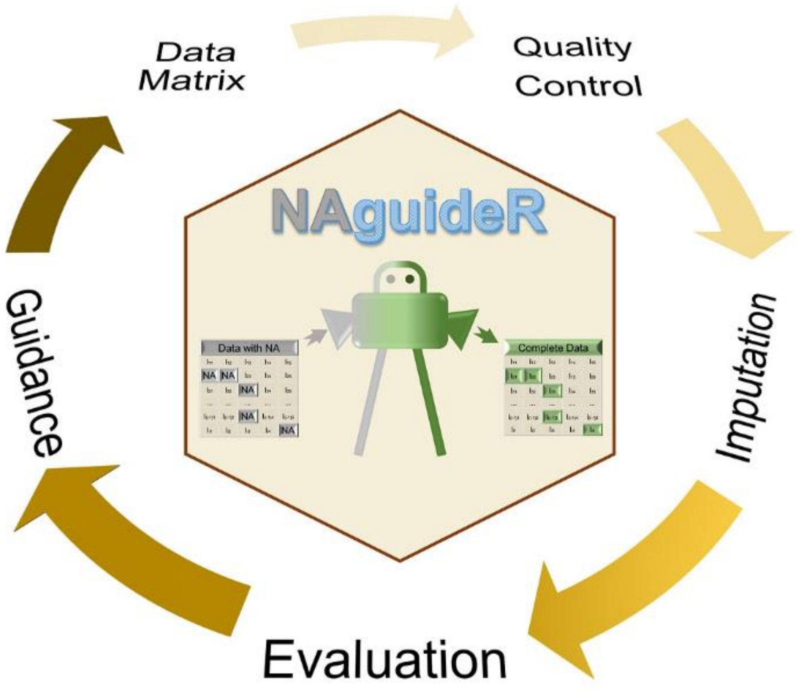

Basically, there are four main steps in NAguideR:

1. Uploading proteomics expression data and sample information data;
2. Data quality control;
3. Missing value imputation;
4. Performance evaluation;

After this, NAguideR can provide valuable guidance for users to select one proper method for their own data based on the evaluation results. Detailed introduction can be found in the ***Help*** part.

Finally, NAguideR is developed by R shiny (Version 1.3.2), and is free and open to all users with no login requirement. It can be readily accessed by all popular web browsers including Google Chrome, Mozilla Firefox, Safari and Internet Explorer 10 (or later), and so on. We would highly appreciate that if you could send your feedback about any bug or feature request to Shisheng Wang at wsslearning@omicsolution.com.

*^_^ Enjoy yourself in NAguideR ^_^*

© 2019 Yansheng Liu's Group and Hao Yang's Group. All Rights Reserved.
Created by Shisheng Wang. E-mail: wsslearning@omicsolution.com.

II. Supplementary tables and figures

Table S1. Description of 23 missing value imputation methods.

Class	Abbreviation	Manipulation Method	Algorithm Description	Remarks & Suggestions	Function	Speed	Package/References
**1. Single value methods (*SV methods*), which mean replacing missing values by a constant or a randomly selected value.**	zero	zero	Replaces the missing values by 0.	These algorithms are relatively simple and fast. However, they may introduce severe bias in data.	0	Fast	base (1)
	minimum	minimum	Replaces the missing values by the smallest non-missing value in the data.		min		base (2)
	colmedian	Column median	Replaces the missing values by the median of non-missing value in each column.		impute		e1071 (3)
	rowmedian	Row median	Replaces the missing values by the median of non-missing value in each row.		impute		e1071 (3)
	Mindet	Deterministic minimum imputation	Perform the imputation of left-censored missing data using a deterministic minimal value approach. Considering an expression data with n samples and p features, for each sample, the missing entries are replaced with a minimal value observed in that sample. The minimal value observed is estimated as being the q-th quantile of the observed values in that sample.		impute.MinDet		imputeLCMD (4)
			Performs the imputation of left-censored missing data by random draws from a Gaussian distribution centred to a		impute.MinProb		imputeLCMD (4)

	Minprob	Probabilistic minimum imputation	minimal value. Considering an expression data matrix with n samples and p features, for each sample, the mean value of the Gaussian distribution is set to a minimal observed value in that sample. The minimal value observed is estimated as being the q-th quantile of the observed values in that sample. The standard deviation is estimated as the median of the feature standard deviations.				
	PI	Perseus imputation	Replace missing values from normal distribution		rnorm		base (5)
**2. Global structure methods (*GS methods*), which decompose the data matrix or minimize the determinant of the covariance and then iteratively reconstruct the**	SVD	Singular value decomposition imputation	Initializes all missing elements with zero then estimate them as a linear combination of the k most signifcant eigen-variables iteratively until reaches certain convergence threshold.	These models assume the existence of a global covariance structure among all samples or objects (i.e., proteins/peptides/genes) in the expression matrix. When this assumption is not appropriate, for example, when the proteins exhibit dominant local similarity structures, their imputation may become less accurate.	svdPca	Fast	pcaMethods (6)
	BPCA	Bayesian PCA missing value estimation	An iterative method using a Bayesian model to handle missing values.		bpca	Slow	pcaMethods (7)
	MLE	Imputation based on maximum likelihood estimation	Maximum likelihood-based imputation method using the EM algorithm.		prelim.norm, em.norm, imp.norm	Fast	norm (8)

**missing values.**	Impseq	Sequential imputation of missing values	Estimates sequentially the missing values in an incomplete observation by minimizing the determinant of the covariance of the augmented data matrix. Then the observation is added to the complete data matrix and the algorithm continues with the next observation with missing values.		impSeq		rrcovNA (9)
	Impseqrob	Robust sequential imputation of missing values	Similar to Impseq, but improved by plugging in robust estimators of location and scatter.		impSeqRob		rrcovNA (10)
	KNN	K Nearest Neighbors imputation	K-nearest neighbors in the space of peptides/proteins to impute missing expression values.	In these models, only a subset of objects (i.e., proteins/peptides/genes) that exhibits high correlation with one object (i.e., protein/peptide/gene) containing the missing values is used to compute the missing values in the object. Therefore, their imputation can be more accurate when a strong local correlation exists between objects in the data, otherwise, they may not perform well.	impute.knn	Fast	impute (6)
	Seq-KNN	Sequential K-nearest neighbor	Imputes the missing values sequentially from the peptide/protein having least missing values based on KNN method, and uses the imputed values for the later imputation.		SeqKNN		SeqKnn (11)
	trKNN	Truncation k-nearest neighbors imputation	Applies a Newton-Raphson (NR) optimization to estimate the truncated mean and standard deviation. Then, Pearson correlation was calculated based on standardized data followed by correlation-based kNN imputation.		sim_trKNN_wrapper	Slow	Imput_funcs.R (12)

**3. Local similarity methods (*LS methods*), which exploit local similarity structure based on the expression profiles of those objects (etc. peptides, proteins) in the data.**	LLS	Local least squares imputation	K variables (peptides/ proteins) are selected by Pearson, spearman or Kendall correlation coefficients. Then missing values are imputed by a linear combination of the k selected variables. The optimal combination is found by LLS regression.		llsImpute	Fast	pcaMethods (13)
	QR	Quantile regression imputation of left-censored data	A missing data imputation method that performs the imputation of left-censored missing data using random draws from a truncated distribution with parameters estimated using quantile regression.		impute.QRILC		imputeLCMD (14)
	IRM	Iterative robust model-based imputation	In each step of the iteration, one variable is used as a response variable and the remaining variables serve as the regressors.		irmi	Slow	VIM (15)
	GRR	Glmnet Ridge Regression	A prediction model is employed for the prediction of missing values by setting a targeted missing variable as outcome and other variables as predictors. Here Glmnet Ridge Regression model is applied as a prediction model.		impute.RegImpute		DreamAI (16)
	GMS	Generalized Mass Spectrum missing peaks	Applies a Lasso model to select subsets of detected peaks to predict the missing values using a two-step procedure, two-step Lasso (TS-Lasso).		GMS.Lasso		GMSimpute (17)

		imputation with Two-Step Lasso					
	Mice-norm	Multivariate Imputation by Chained Equations-Bayesian linear regression	Generates multiple imputations for incomplete multivariate data by Gibbs sampling. Missing data can occur anywhere in the data. The algorithm imputes an incomplete column (the target column) by generating 'plausible' synthetic values given other columns in the data. Each incomplete column must act as a target column, and has its own specific set of predictors. The default set of predictors for a given target consists of all other columns in the data. For predictors that are incomplete themselves, the most recently generated imputations are used to complete the predictors prior to imputation of the target column. The imputation method depends on Bayesian linear regression.		mice (method='norm')	Slow	mice (18)
	Mice-cart	Multivariate Imputation by Chained Equations-classification	Generates multiple imputations for incomplete multivariate data by Gibbs sampling. Missing data can occur anywhere in the data. The algorithm imputes an incomplete column (the target		mice (method='cart')	Slow	mice (18)

		and regression trees	column) by generating 'plausible' synthetic values given other columns in the data. Each incomplete column must act as a target column, and has its own specific set of predictors. The default set of predictors for a given target consists of all other columns in the data. For predictors that are incomplete themselves, the most recently generated imputations are used to complete the predictors prior to imputation of the target column. The imputation method depends on classification and regression trees.				
	RF	Random forest	Imputes missing values particularly in the case of mixed-type data based on a random forest. It can be used to impute continuous and/or categorical data including complex interactions and nonlinear relations. It yields an out-of-bag (OOB) imputation error estimate.		missForest		missForest (19)

Table S2. The summary of *NAguideR* tested on different operation systems and browsers.

Operation System	Version	Chrome	Firefox	Safari
Windows	7	68.0.3440.106	63.0.3	not tested
Linux	CentOS 7	not tested	52.8.0	not tested
MacOS	HighSierra	70.0.3538.110	not tested	12.0.1

Table S3. The number of detected peptides/proteins and the proportion of missing values in each data set.

Level	Peptide level		Protein level
Dataset	PhosDIA	PepSWATH	ProtSWATH
Total number	54,076	57,687	4,797
Missing value number (%)	41,262 (76.3)	31,769 (55.1)	981 (20.4)
Number after filtered	13,946	36,363	3,640

Note: ‘Total number’ here means the identified peptides/proteins number in each dataset. ‘Missing value number’ means the number of quantified peptides/proteins with missing value in at least one sample, the number in parentheses is the rate of missing value corresponding to “Total number”. ‘Number after filtered’ means the number of quantified peptides/proteins after removing those with high proportion of missing values and coefficient of variation (e.g., those peptides/proteins with 50% proportion of missing values or coefficient of variation above 30% will be removed).

Figure S1. Distribution of the time consumption of each imputation method. Results were obtained from the ProtSWATH dataset, only for the demonstration of speed difference between methods. We repeated 100 times for every method Note, the time is just a reference for users because it is also related to data size and internet status (or whether computer hardware configuration if running *NAguideR* locally). Obviously, if the data size is smaller and internet speed is fast, the imputation time will be less.

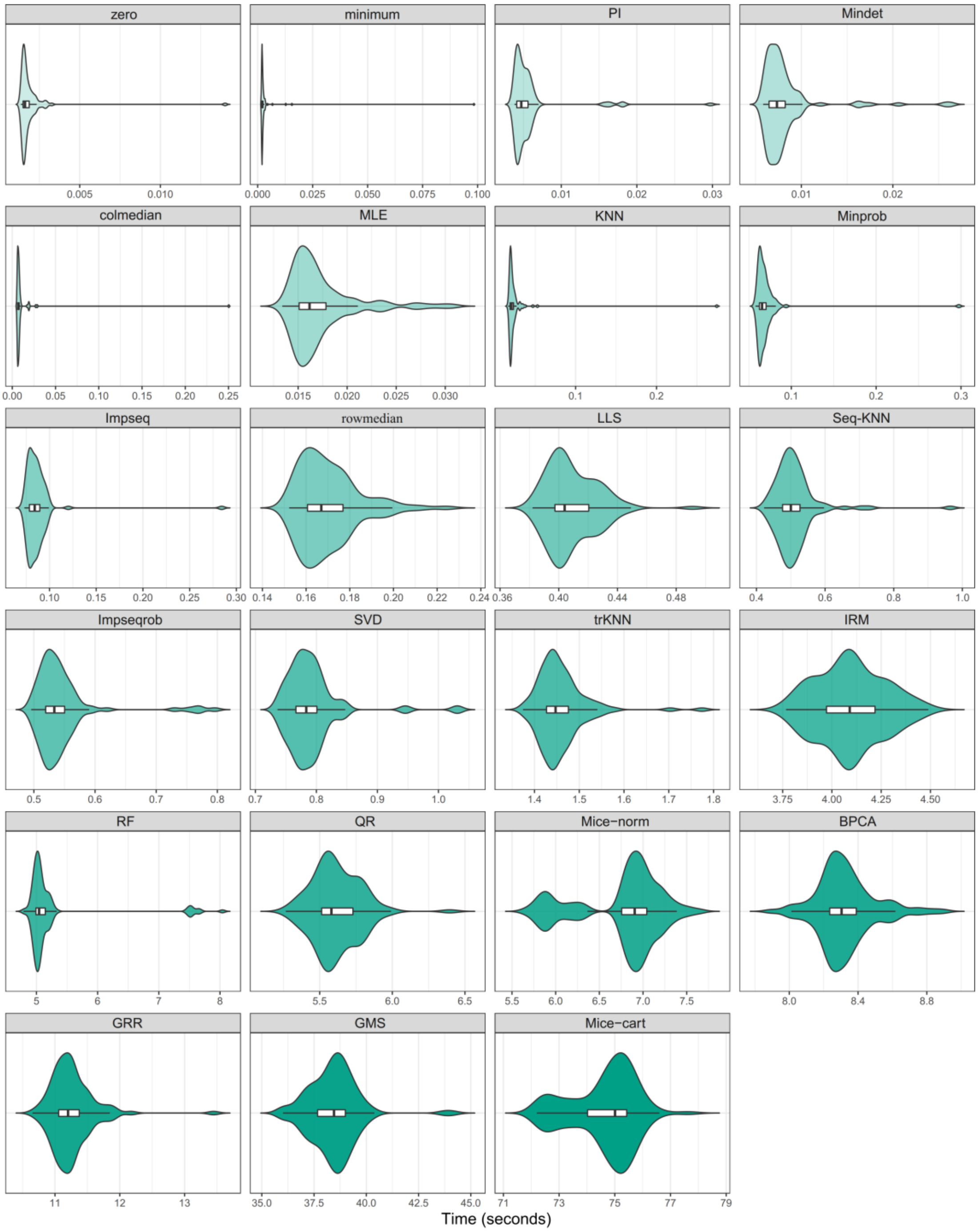

Figure S2. Illustration of major steps of the data analysis process in *NAguideR*. We take two groups of samples (five biological replicates in each group, labeled A1, A2, A3, A4, A5, B1, B2, B3, B4, B5 in the original intensity data), just for the illustrative example. “Feature” here denotes the identified proteins/peptides.

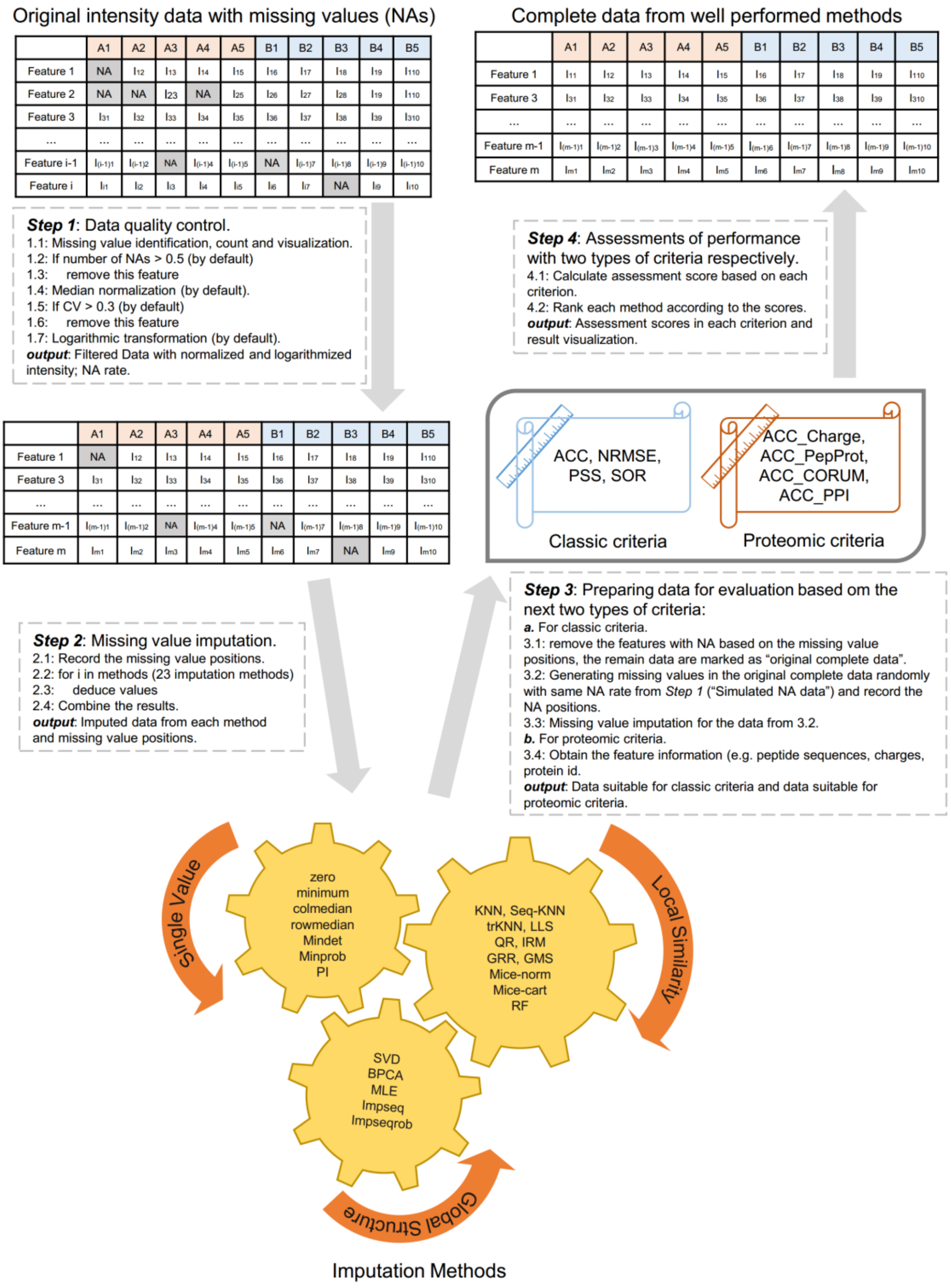

Figure S3. Distribution of missing values in all the three example datasets. (A-B) Missing value distribution of each sample and every feature in PhosDIA dataset. (C-D) Missing value distribution of each sample and every feature in PepSWATH dataset. (E-F) Missing value distribution of each sample and every feature in ProtSWATH dataset. 'Feature' here denotes a peptide or protein.

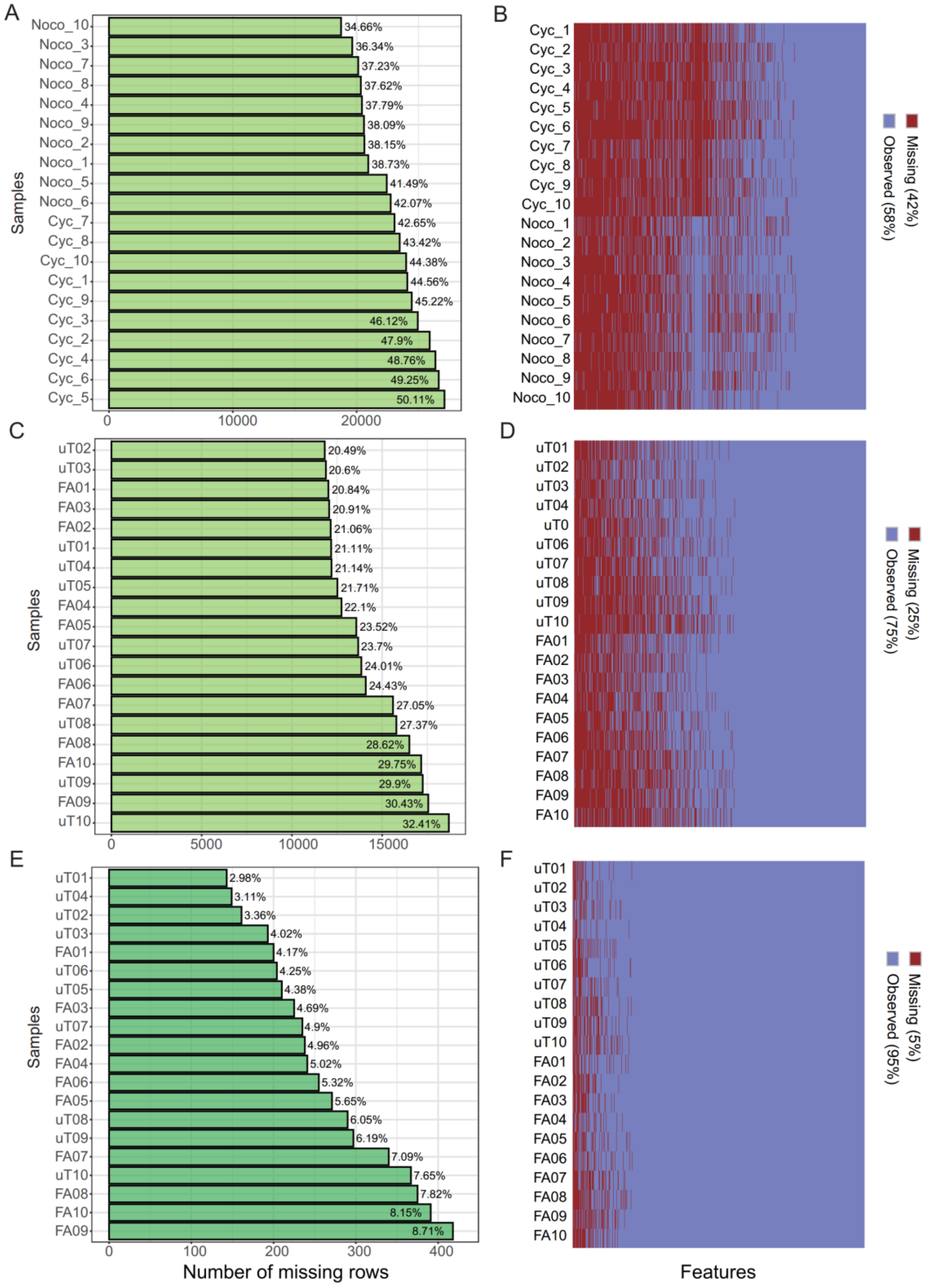

Figure S4. Comparisons of original values and imputed values of every peptide from every imputation method on the extracted complete data matrix from PhosDIA. The adjusted R squared of each result was also obtained by 'lm' function and shown in for each method (except zero and minimum method). We first only extracted the complete data matrix and generated random missing values on it with a similar proportion of missing values existed in the original data matrix. Thus, every imputed data point will have a real reference (i.e., the original value) for correlation analysis.

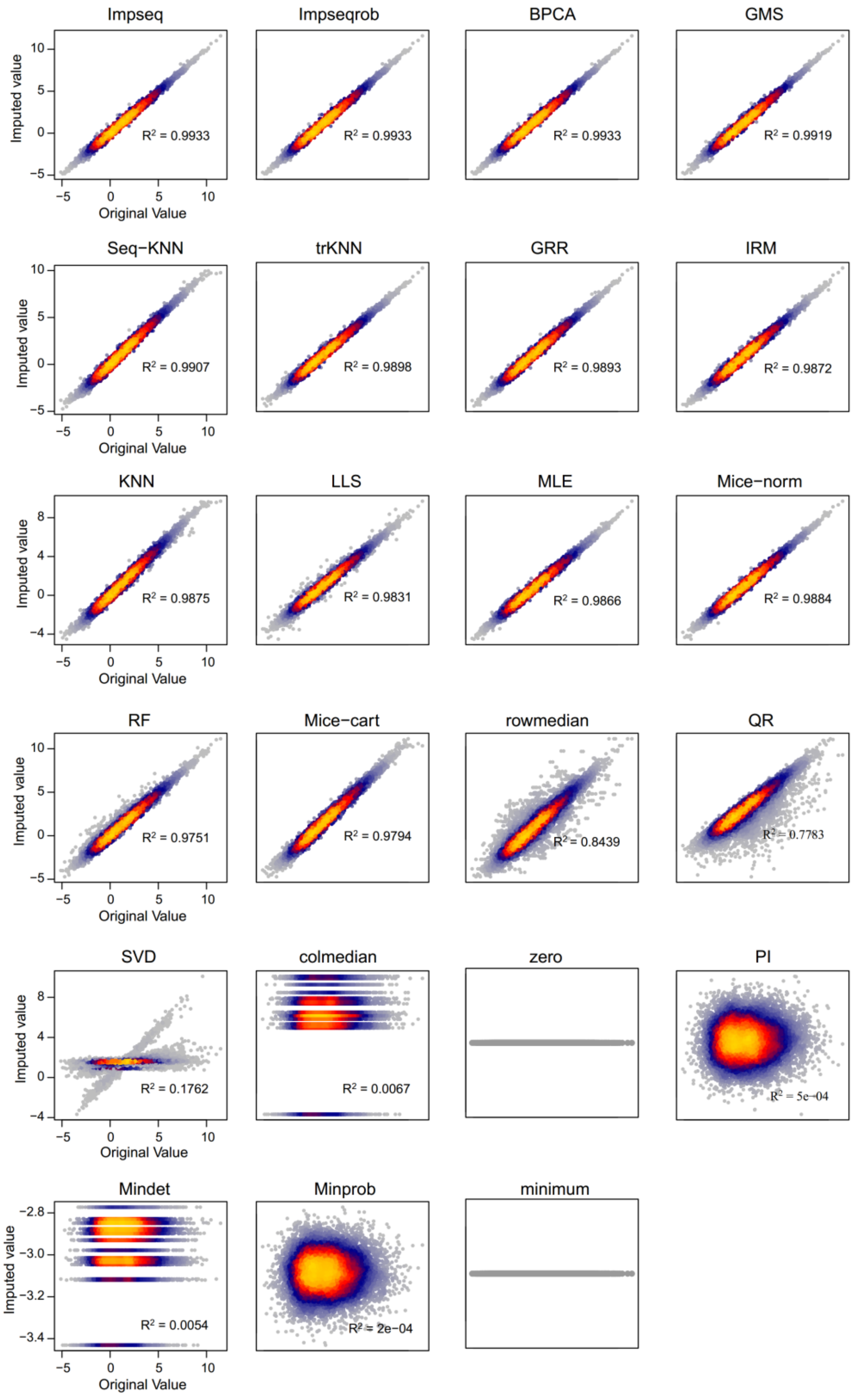

Figure S5. Systematic evaluation analysis of the pepSWATH dataset (Similar to Figure 2). (A) Pearson correlation analysis of the original intensities and imputed intensities based on 23 methods. Density plots illustrate the correlation in detail between the original values and imputed values from minimum, SVD, and Impseqrob respectively. NA here means 'No Result' because the standard deviations of imputed values from zero and minimum method are equal to 0 and hence the cor function returns NA. (B) Comparison of the distribution of the correlation coefficient among original values and 23 imputation methods under the four proteomic criteria. The comprehensive scores distribution of 23 imputation methods under the four classic criteria (C) and four proteomic criteria (D). 'Normalized Values' here means every score is divided by corresponding maximum value.

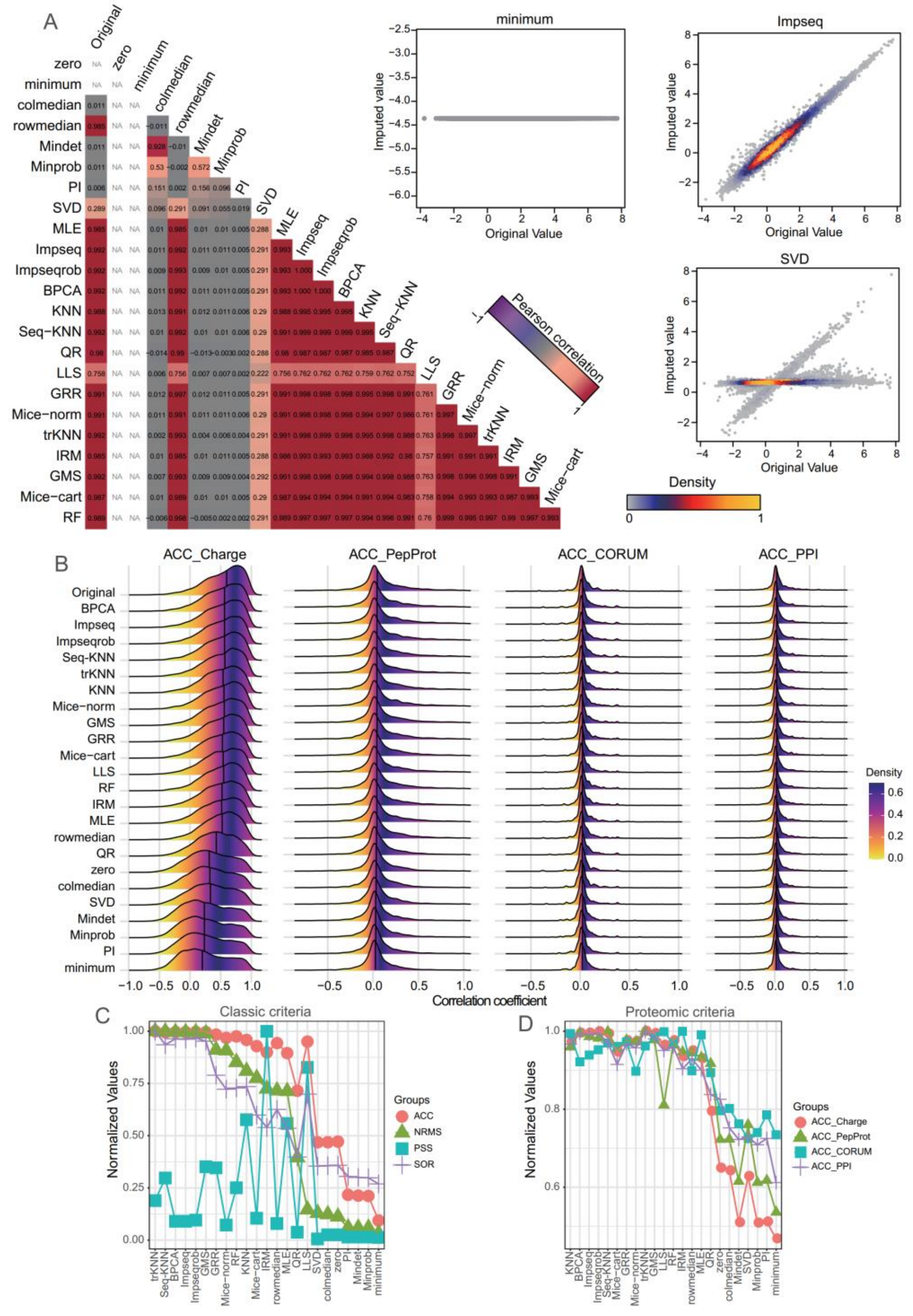

Figure S6. Systematic evaluation analysis of the ProtSWATH dataset (Similar to Figure 2). (A) Pearson correlation analysis of the original intensities and imputed intensities based on 23 methods. Density plots illustrate the correlation in detail between the original values and imputed values from minimum, SVD, and Impseqrob respectively. NA here means ‘No Result’ because the standard deviations of imputed values from zero and minimum method are equal to 0 and hence the cor function returns NA. (B) Comparison of the distribution of the correlation coefficient among original values and 23 imputation methods under the four proteomic criteria. The comprehensive scores distribution of 23 imputation methods under the four classic criteria (C) and four proteomic criteria (D). ‘Normalized Values’ here means every score is divided by corresponding maximum value.

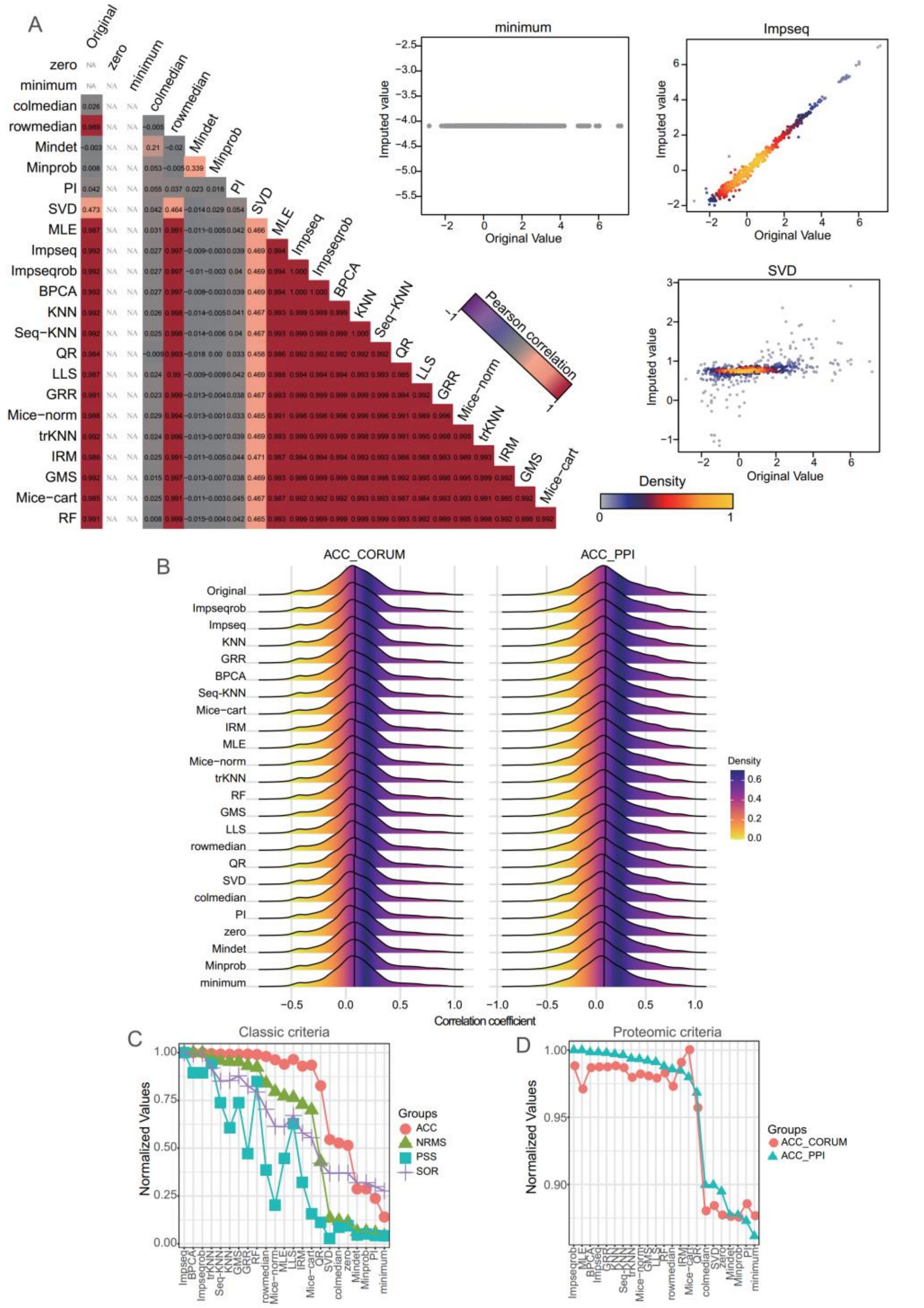

Figure S7. Comparisons of original values and imputed values of the correlation coefficients among peptides that are derived under ACC_Charge criterion across every imputation method that was directly applied on the full PhosDIA dataset. The adjusted R squared of each result was also obtained by ‘lm’ function and shown for each imputation method.

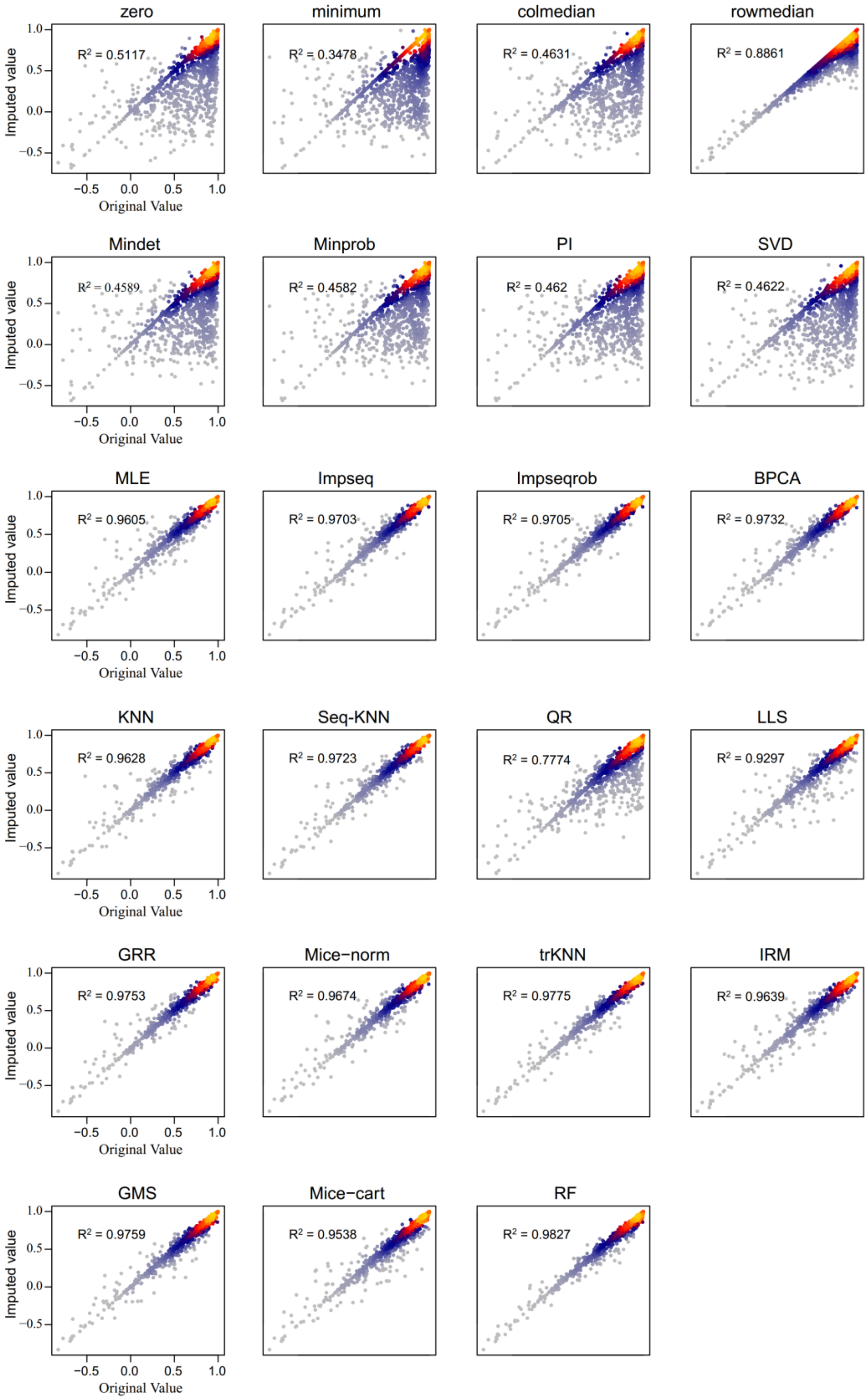

Figure S8. Evaluation of every imputation method across different missing proportions on the three proteomics datasets under the proteomic criteria (A: PhosDIA, B: PepSWATH, C: ProtSWATH). The proportion of missing values is from 5% to 70% in step of 5%. The lower right part shows the imputation method names with relative marked colors and the grey arrow facilitates the reading of the relative rank of every method.

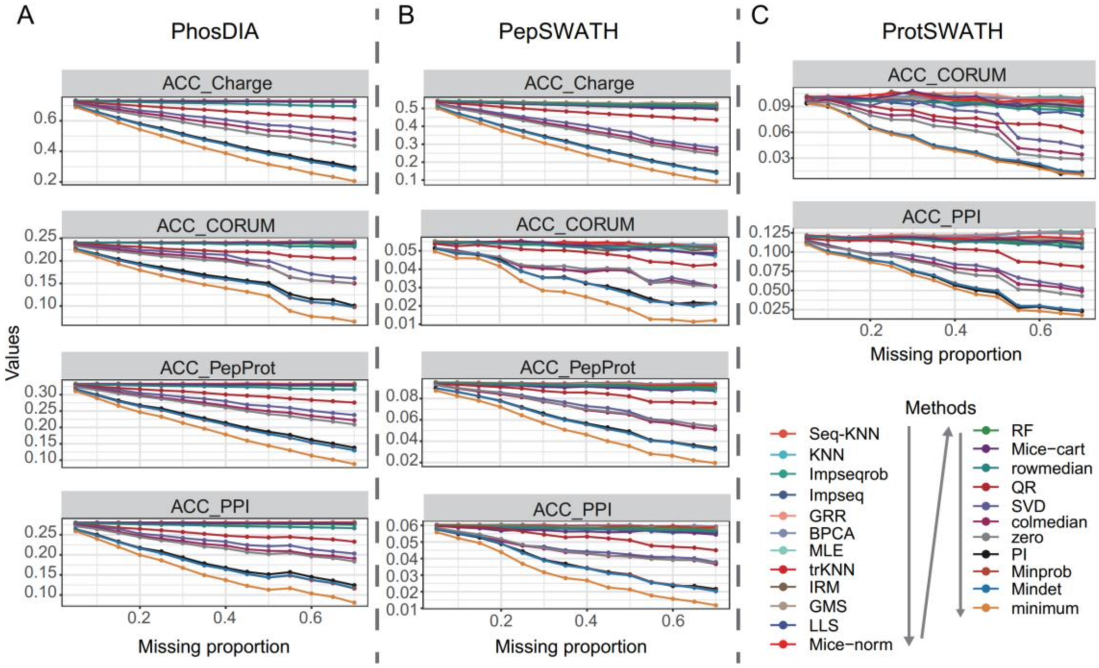

Figure S9. The score distribution of every imputation methods based on the classic criteria in the three proteomics datasets with different biological replicates (Left: PhosDIA, middle: PepSWATH, right: ProtSWATH). 'Normalized Values' here means every score is divided by corresponding maximum value. '10 VS 10' means there are 10 replicates in each group (marked with darkblue color), and '3 VS 3' means there are 3 replicates in each group (marked with red color).

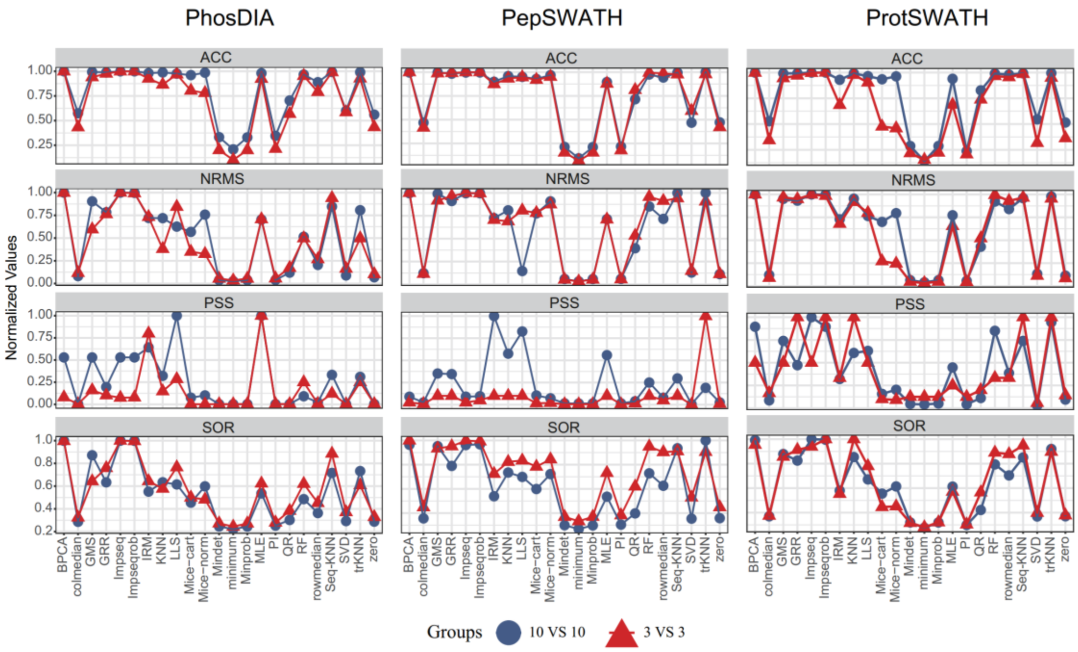

Figure S10. Comparison of the root mean square error (RMSE) of the average correlation coefficients across sample among each method on the pepSWATH data set. (A) The distribution of the across sample correlation coefficient RMSE among original, Requant and 23 imputation methods under the four proteomic criteria. (B) The normalized RMSE distribution of Requant and 23 imputation methods under the four proteomic criteria. 'Normalized Values' here means every RMSE divides by corresponding max value. "Requant" means "Requantification" method in OpenSWATH.

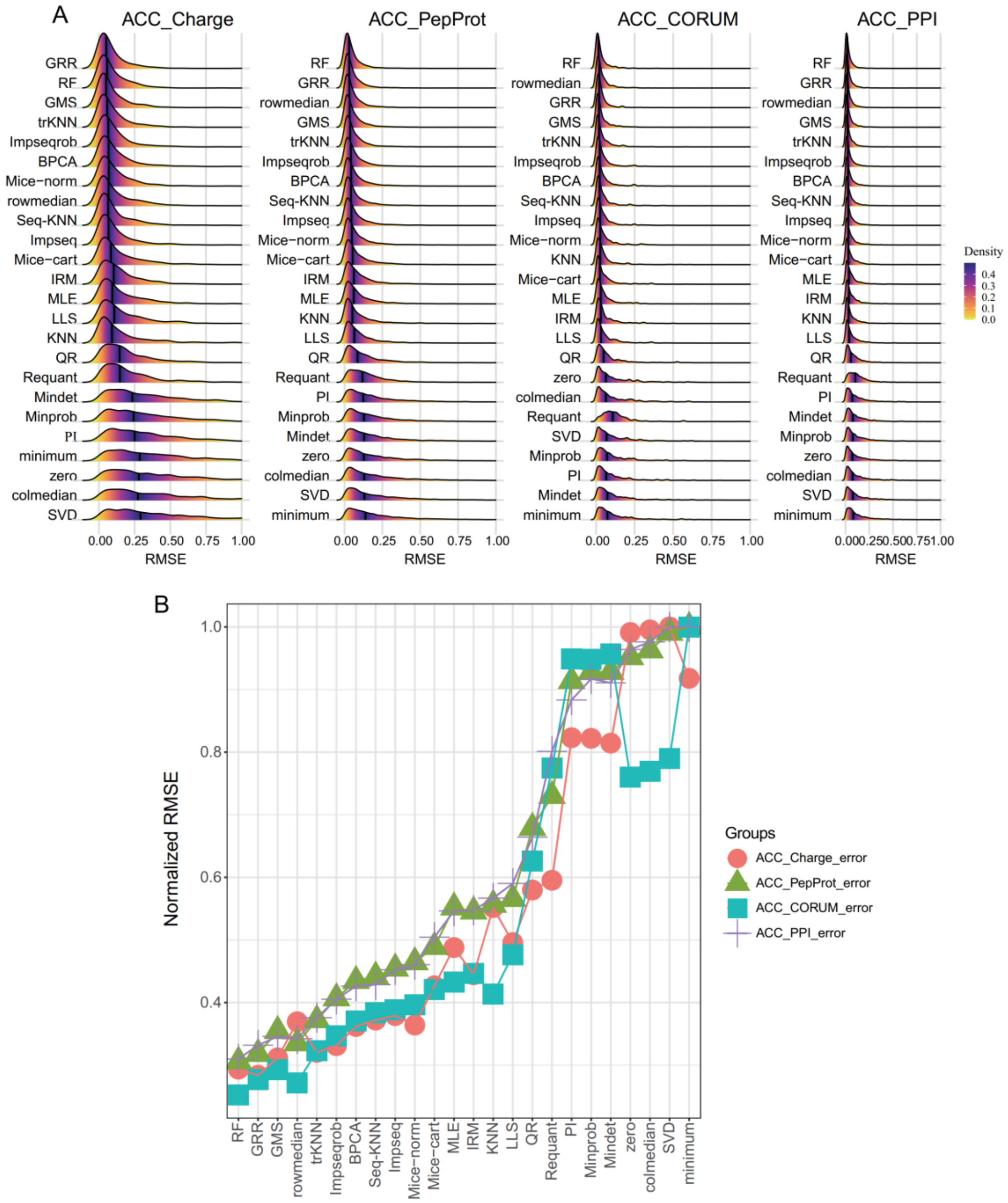

Figure S11. Volcano plots examples for differential expression analysis in PhosDIA (following Figure 5). (A) From original full data (labelled as 'Gold Standard'), imputed data of randomly selected 5 biological replicates (labelled as Random 5) (B-D) and 3 biological replicates (labelled as Random 3) (E-G) in each group from Imseq, Seq-KNN, minimum method, respectively.

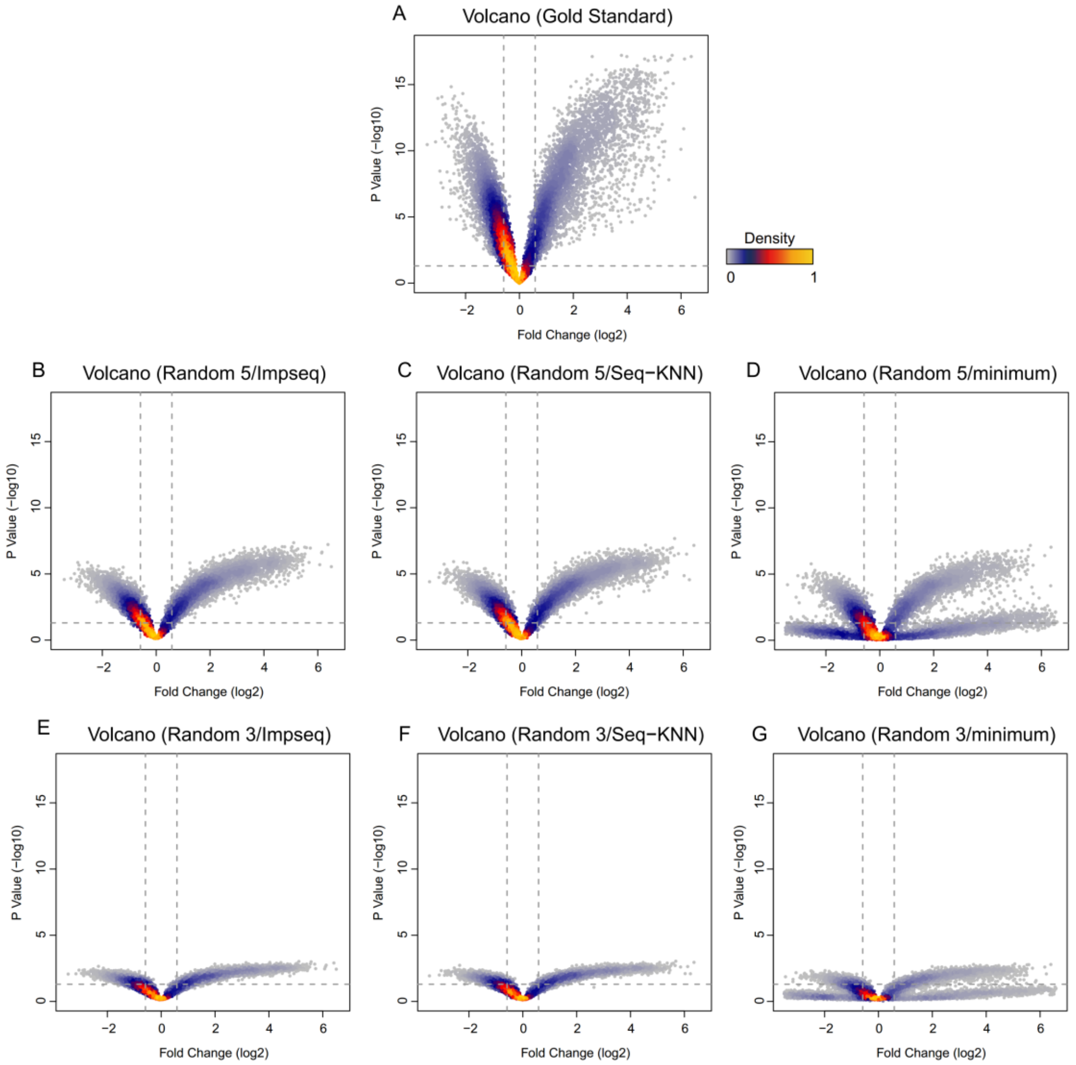

Figure S12. Motif analysis of the differentially expressed peptides in the PhosDIA dataset. (A) Venn diagram of the differential peptides identified in the first 3 biological replicates with Seq-KNN method (First 3.seqknn) and zero method (First 3.zero). (B) Venn diagram of identified motifs from the 'Gold standard' dataset (Gold.standard) and those peptides identified in First 3.seqknn dataset but not in First 3.zero dataset (First 3.seqknn.zero.diff). (C) Detailed motif illustrations. Note that the last motif seems to be newly identified from First 3.zero or First 3.seqknn.zero.diff, whereas it actually can be derived from the inspection of Gold.standard result.

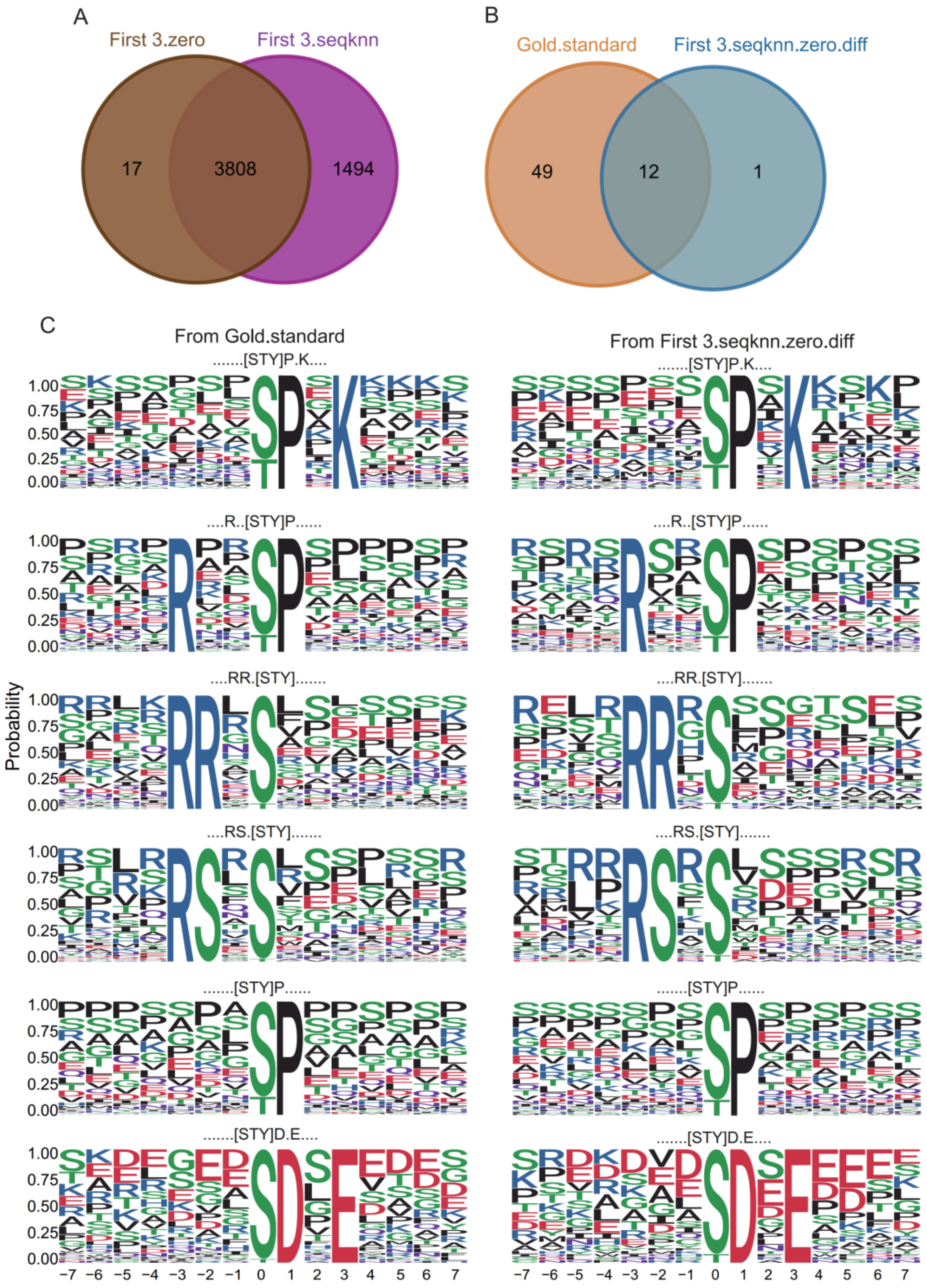

Figure S12C continued.

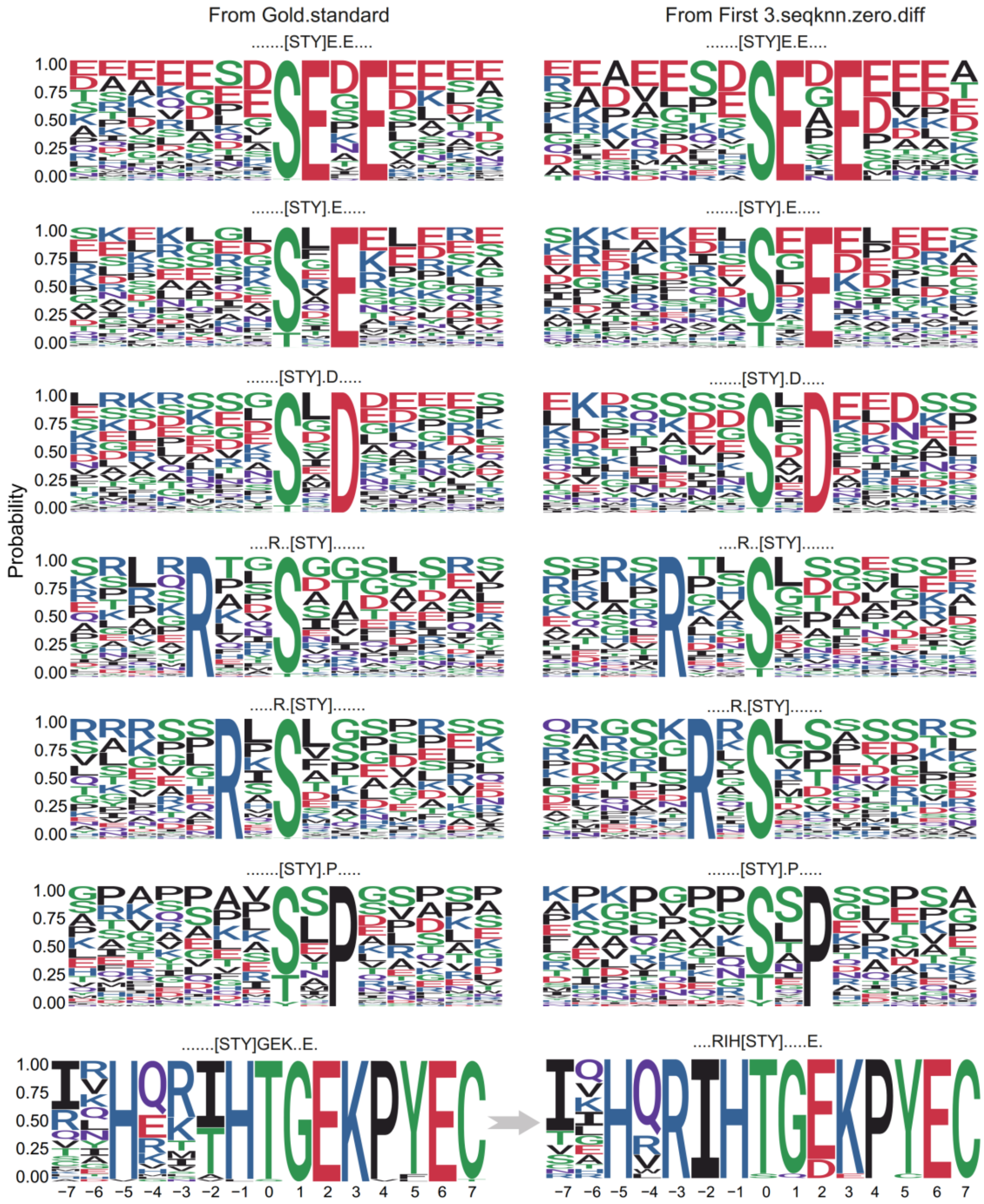

III. References

1. Lazar, C., Gatto, L., Ferro, M., Bruley, C. and Burger, T. (2016) Accounting for the Multiple Natures of Missing Values in Label-Free Quantitative Proteomics Data Sets to Compare Imputation Strategies. *J Proteome Res*, **15**, 1116-1125.
2. Jiang, Y., Sun, A., Zhao, Y., Ying, W., Sun, H., Yang, X., Xing, B., Sun, W., Ren, L., Hu, B. *et al.* (2019) Proteomics identifies new therapeutic targets of early-stage hepatocellular carcinoma. *Nature*, **567**, 257-261.
3. Dimitriadou, E., Hornik, K., Leisch, F., Meyer, D. and Weingessel, A. (2008) Misc functions of the Department of Statistics (e1071), TU Wien. *R package*, **1**, 5-24.
4. Webb-Robertson, B.-J.M., Wiberg, H.K., Matzke, M.M., Brown, J.N., Wang, J., McDermott, J.E., Smith, R.D., Rodland, K.D., Metz, T.O. and Pounds, J.G. (2015) Review, evaluation, and discussion of the challenges of missing value imputation for mass spectrometry-based label-free global proteomics. *J Proteome Res*, **14**, 1993-2001.
5. Tyanova, S., Temu, T., Sinitcyn, P., Carlson, A., Hein, M.Y., Geiger, T., Mann, M. and Cox, J. (2016) The Perseus computational platform for comprehensive analysis of (prote)omics data. *Nature methods*, **13**, 731-740.
6. Troyanskaya, O., Cantor, M., Sherlock, G., Brown, P., Hastie, T., Tibshirani, R., Botstein, D. and Altman, R.B. (2001) Missing value estimation methods for DNA microarrays. *Bioinformatics*, **17**, 520-525.
7. Oba, S., Sato, M.-a., Takemasa, I., Monden, M., Matsubara, K.-i. and Ishii, S. (2003) A Bayesian missing value estimation method for gene expression profile data. *Bioinformatics*, **19**, 2088-2096.
8. Ibrahim, J.G., Chen, M.-H., Lipsitz, S.R. and Herring, A.H. (2005) Missing-data methods for generalized linear models: A comparative review. *Journal of the American Statistical Association*, **100**, 332-346.
9. Verboven, S., Branden, K.V. and Goos, P. (2007) Sequential imputation for missing values. *Computational Biology and Chemistry*, **31**, 320-327.
10. Branden, K.V. and Verboven, S. (2009) Robust data imputation. *Computational Biology and Chemistry*, **33**, 7-13.
11. Kim, K.-Y., Kim, B.-J. and Yi, G.-S. (2004) Reuse of imputed data in microarray analysis increases imputation efficiency. *Bmc Bioinformatics*, **5**, 160.
12. Shah, J.S., Rai, S.N., DeFilippis, A.P., Hill, B.G., Bhatnagar, A. and Brock, G.N. (2017) Distribution based nearest neighbor imputation for truncated high dimensional data with applications to pre-clinical and clinical metabolomics studies. *Bmc Bioinformatics*, **18**, 114.
13. Kim, H., Golub, G.H. and Park, H. (2004) Missing value estimation for DNA microarray gene expression data: local least squares imputation. *Bioinformatics*, **21**, 187-198.
14. Wei, R., Wang, J., Su, M., Jia, E., Chen, S., Chen, T. and Ni, Y. (2018) Missing value imputation approach for mass spectrometry-based metabolomics data. *Scientific reports*, **8**, 663.
15. Templ, M., Kowarik, A. and Filzmoser, P. (2011) Iterative stepwise regression imputation using standard and robust methods. *Computational Statistics & Data Analysis*, **55**, 2793-2806.
16. Wei, R., Wang, J., Jia, E., Chen, T., Ni, Y. and Jia, W. (2018) GSimp: A Gibbs sampler based left-censored missing value imputation approach for metabolomics studies. *Plos Comput*

Biol, **14**, e1005973.

17. Li, Q., Fisher, K., Meng, W., Fang, B., Welsh, E., Haura, E.B., Koomen, J.M., Eschrich, S.A., Fridley, B.L. and Chen, Y.A. (2020) GMSimpute: a generalized two-step Lasso approach to impute missing values in label-free mass spectrum analysis. *Bioinformatics*, **36**, 257-263.
18. Buuren, S.v. and Groothuis-Oudshoorn, K. (2010) mice: Multivariate imputation by chained equations in R. *Journal of statistical software*, 1-68.
19. Kokla, M., Virtanen, J., Kolehmainen, M., Paananen, J. and Hanhineva, K. (2019) Random forest-based imputation outperforms other methods for imputing LC-MS metabolomics data: a comparative study. *Bmc Bioinformatics*, **20**, 1-11.
20. Tyanova, S., Temu, T. and Cox, J. (2016) The MaxQuant computational platform for mass spectrometry-based shotgun proteomics. *Nat Protoc*, **11**, 2301-2319.
21. Ma, B., Zhang, K., Hendrie, C., Liang, C., Li, M., Doherty-Kirby, A. and Lajoie, G. (2003) PEAKS: powerful software for peptide de novo sequencing by tandem mass spectrometry. *Rapid communications in mass spectrometry : RCM*, **17**, 2337-2342.
22. Bruderer, R., Bernhardt, O.M., Gandhi, T., Miladinović, S.M., Cheng, L.-Y., Messner, S., Ehrenberger, T., Zanotelli, V., Butscheid, Y. and Escher, C. (2015) Extending the limits of quantitative proteome profiling with data-independent acquisition and application to acetaminophen-treated three-dimensional liver microtissues. *Mol Cell Proteomics*, **14**, 1400-1410.
23. Demichev, V., Messner, C.B., Vernardis, S.I., Lilley, K.S. and Ralser, M. (2020) DIA-NN: neural networks and interference correction enable deep proteome coverage in high throughput. *Nature methods*, **17**, 41-44.
24. Rost, H.L., Rosenberger, G., Navarro, P., Gillet, L., Miladinovic, S.M., Schubert, O.T., Wolski, W., Collins, B.C., Malmstrom, J., Malmstrom, L. *et al.* (2014) OpenSWATH enables automated, targeted analysis of data-independent acquisition MS data. *Nature biotechnology*, **32**, 219-223.
25. Bruderer, R., Bernhardt, O.M., Gandhi, T., Miladinovic, S.M., Cheng, L.Y., Messner, S., Ehrenberger, T., Zanotelli, V., Butscheid, Y., Escher, C. *et al.* (2015) Extending the limits of quantitative proteome profiling with data-independent acquisition and application to acetaminophen-treated three-dimensional liver microtissues. *Molecular & cellular proteomics : MCP*, **14**, 1400-1410.
26. Bruderer, R., Bernhardt, O.M., Gandhi, T., Xuan, Y., Sondermann, J., Schmidt, M., Gomez-Varela, D. and Reiter, L. (2017) Optimization of Experimental Parameters in Data-Independent Mass Spectrometry Significantly Increases Depth and Reproducibility of Results. *Molecular & cellular proteomics : MCP*, **16**, 2296-2309.
